# Why Biden-era clean energy investment policies had limited political returns

**DOI:** 10.1073/pnas.2526802123

**Published:** 2026-02-26

**Authors:** Alexander F. Gazmararian, Nathan M. Jensen, Dustin Tingley

**Affiliations:** ^a^Department of Political Science, University of Michigan, Ann Arbor, MI 48109; ^b^Department of Government, University of Texas at Austin, Austin, TX 78712; ^c^Department of Government, Harvard University, Cambridge, MA 02138

**Keywords:** climate change, public opinion, firm behavior, green industrial policy, policy feedback

## Abstract

The Biden Administration enacted the largest federal policy framework to incentivize clean energy and decarbonization in U.S. history. We examine whether Biden-era green investments produced political returns by affecting public opinion. Using geolocated survey data linked to investment records and a database of company and politician statements, we assess project visibility and credit attribution. People closer to new renewable energy and green manufacturing facilities are more likely to notice these investments but are not more likely to credit the Biden Administration. Instead, the public sees governors as most responsible. This credit allocation pattern aligns with the political message environment: Governors more frequently claim credit than the White House and companies spread recognition broadly across political actors. This fragmented information environment illustrates the limits of using less traceable forms of green spending to generate electoral gains and public support for climate policy.

## Significance

The Biden Administration enacted over $198 billion in clean energy and manufacturing incentives, with the expectation that delivering material economic benefits could yield political dividends. This nationwide study examines whether these investments affect public opinion. Although proximity to green projects makes them more visible to the public, it does not bestow credit on the Biden Administration which pushed for them. The most substantial political beneficiaries are governors, who more actively claim credit than the White House. For policies to affect politics, voters need to be able to trace them back to the responsible political actors, which is challenging in a complex information environment. Green spending channeled through private firms alone is unlikely to build ground-up coalitions for climate policy.

The Biden Administration enacted legislation to reduce U.S. greenhouse gas emissions through large-scale clean energy investments, most notably the clean electricity and manufacturing tax credits in the 2022 Inflation Reduction Act (IRA), estimated to cost $161 billion and $37 billion over ten years (1). Policymakers intended for investments incentivized by federal legislation not only to accomplish economic goals but also to build durable political support for climate policy (2). The logic is that visible material benefits for businesses and voters, such as new jobs, create identifiable beneficiaries who credit incumbent policymakers, helping insulate those officials and the policies they enact from repeal or retrenchment (3–5).

This paper examines whether Biden-era green investments affect public opinion in ways that could sustain and expand climate reforms. This process is known as policy feedback, where public policy creates supportive constituencies (6, 7). Our focus on public opinion, rather than electoral outcomes, allows us to assess whether the political logic motivating these investments operates at its most basic level, prior to voting or legislative conflict.

There are two necessary conditions for green investments to yield political returns where they are built. Voters must notice projects and link them to the responsible policymakers (8). Visibility and traceability are not guaranteed. While voters can acquire political information from what they see in their communities (9–11), clean energy projects may not be visible initially due to construction delays. Credit attribution could also be muddled, since governors and local officials often play a role and claim responsibility (12), and partisanship clouds how the public receives and interprets information (13). It is not automatic that federal policymakers behind green spending will receive credit—a prerequisite for electoral returns.

Evidence about the effect of Biden-era clean energy investments on public opinion is limited due to the recency of these projects. Few surveys capture whether people notice local investments and, if so, whom they credit. Company and politician statements, which shape the public’s information environment, have also not yet been systematically collected since they are scattered across thousands of announcements.

We collected data to study the mechanisms by which green investments could create positive policy feedbacks. We fielded three geolocated national surveys in 2024, with questions tailored to measure the visibility and traceability of clean energy projects. We also constructed a database of statements covering all green manufacturing investments announced between 2022 and 2024. These data allow us to examine how people, businesses, and politicians respond to these projects.

We find modest evidence that Biden-era green investments are visible, but no sign that people closer to these projects—the intended beneficiaries—are more likely to credit the White House. Even when focusing exclusively on operational manufacturing facilities, residents are more likely to notice them but not to credit federal policymakers who backed green subsidies. Overall, Americans view their governors as more responsible than President Biden.

We posit and test an informational mechanism to explain these credit attribution patterns. Voters form their beliefs in a mixed information environment. State politicians have electoral incentives to claim responsibility (12), businesses strategically diffuse credit across multiple actors to avoid appearing partisan and to maintain broad support, while federal policymakers have limited capacity to claim credit for every project. These conflicting messages could dilute the public’s recognition of the White House’s role.

Our analysis of company and politician statements is consistent with the informational mechanism. Governors are far more active in claiming credit than the Biden Administration, which is most vocal immediately after the IRA’s passage whereas governors sustain their messaging. Companies also issue statements that spread recognition broadly, emphasizing state and local actors. In this fog, green investments are visible but not traceable, limiting their ability to create climate coalitions.

## Research Design

### Project Proximity, Visibility, and Credit Attribution.

We conducted three national online surveys of U.S. adults in 2024 (total N=5,026) to examine whether Biden-era clean energy investments influence public opinion. Respondents reported whether they had seen a new project in their community and rated the responsibility of political actors for causing the investment. *Materials and Methods* describes question wording and validation.

Our aim is to estimate the effect of being near a new investment on an individual’s recognition of the project (visibility) and perception of the Biden Administration’s responsibility (credit attribution). Relying on self-reported information about proximity to green projects would be biased by the survey-taker’s partisanship (14), and crucially could not assess whether new investments are visible. Therefore, the analysis uses an objective measure: the respondent’s distance to new clean energy projects. Respondents are grouped into national distance quintiles, though results are robust to using continuous measures (*SI Appendix*, section S3.5).

**Fig. 1.**
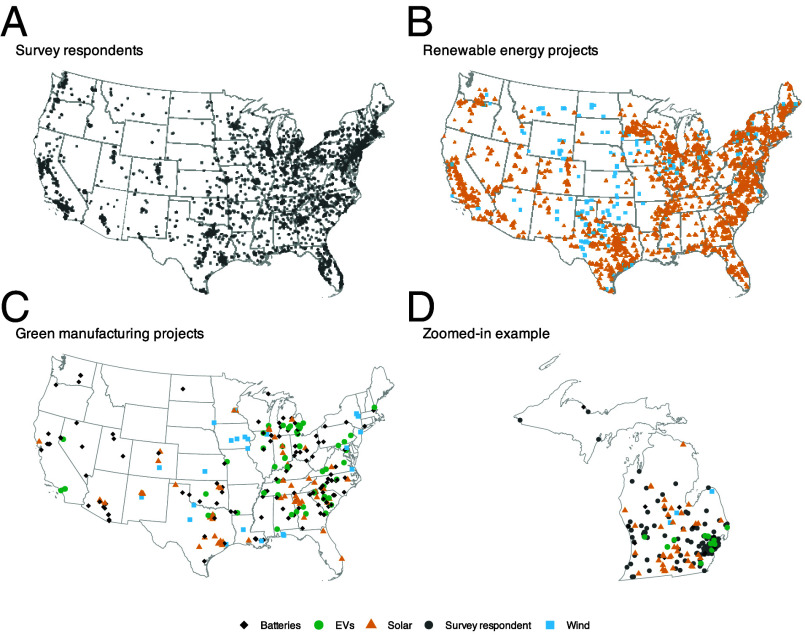
Geographic distribution of survey respondents and post-IRA clean energy investments, 2022-2024 (Alaska and Hawaii not shown). (*A*) Locations of survey respondents. (*B*) Locations of renewable energy projects. (*C*) Locations of green manufacturing projects. (*D*) Zoomed-in example illustrating the spatial relationship between survey respondents and nearby clean energy investments.

The investment data cover 1) utility-scale solar and wind facilities both in preconstruction and under construction, and 2) fully or partly operational clean energy manufacturing sites (Fig. 1). We analyze these projects separately since they may vary in visibility and traceability. While the wind and solar projects are in development, it is plausible that they are visible given media coverage and political credit-claiming even before permanent jobs materialize. Since the manufacturing projects are all operational, they are likely the most visible. To further account for construction time, we also compare operational to nonoperational plants.

The IRA provided substantial federal incentives for green projects, though some also received direct and indirect support through the 2021 Bipartisan Infrastructure Law (BIL) and from state and local policies. While models show that the IRA will accelerate clean energy investments (15), any individual project reflects multiple policy and market forces. Our analysis does not seek to attribute project-level causation to federal policy alone, but rather situates observed investment activity within a policy environment in which the IRA played a central, though not exclusive, role.

While investment takes time, 2023–2024 provides a reasonable window for studying initial policy feedback. Following IRA enactment, there was an immediate increase in planned renewable generation and green manufacturing project announcements, which likely reflects projects receiving incentives with clear statutory language and where Treasury issued partial guidance (*SI Appendix*, section S7). Many IRA programs built on preexisting statutory frameworks, key elements of implementation were already in place, and elected officials had begun claiming credit.

We estimate the effect of proximity by comparing respondents to others in the same state, which holds constant state-level political and economic conditions. Project siting is not random, so we adjust for observable differences, such as infrastructure and local workforce capacity, that could also shape opinions. A causal interpretation relies on the assumption that, after these adjustments, proximity is as-if random within states. Sensitivity and power analyses indicate the design can detect meaningful effects and is robust to plausible unobserved confounding (*SI Appendix*, section S3).

### Company and Politician Statements.

We compiled a comprehensive database of company and politician statements about all clean energy manufacturing projects from August 2022 to December 2024 (327 projects). These large, high-profile manufacturing investments often attract local media coverage and therefore constitute most-likely cases for political credit claiming (12, 16).

The dataset covers statements by companies, governors, U.S. Senators, U.S. Representatives, and the Biden Administration (the president and senior executive-branch officials, e.g., Secretary of Energy). We used a large language model (LLM) to classify whether each statement credited specific actors or policies, capturing explicit claims and implicit actions (e.g., ribbon-cuttings). *Materials and Methods* describes coding procedures and validation.

## Results

### Effect of Proximity on Political Attitudes.

#### Proximity and visibility.

**Fig. 2.**
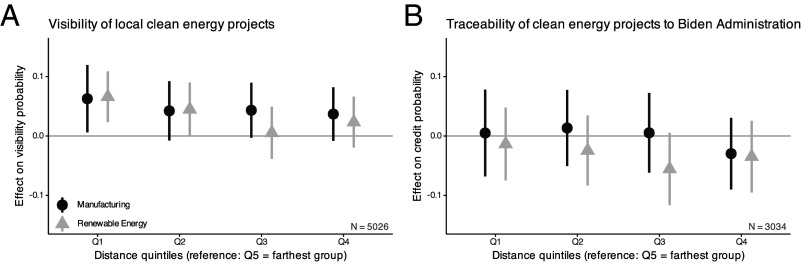
Effect of proximity to post-IRA clean energy projects on public opinion. Points show coefficients from linear regressions of each outcome on proximity quintile indicators, with state and sample fixed effects and respondent covariates. Bars denote 95% confidence intervals based on Conley-adjusted standard errors. Visibility is coded 1 if respondents report seeing a new green project and 0 otherwise; traceability is coded 1 if respondents believe the Biden Administration is responsible for new clean energy projects and 0 otherwise. (*A*) Visibility of local clean energy projects. (*B*) Traceability of clean energy projects to the Biden Administration.

In 2024, 27% of American adults report having seen a new clean energy project in the last year. Proximity increases visibility. Relative to those farthest away from new projects in the same state, respondents in the nearest distance quintile are 6.3 (manufacturing) and 6.6 (renewables) percentage points more likely to say they see a project, with effects extending into the second quintile for renewables (Fig. 2*A*).

The effect of proximity on visibility varies slightly with project phase. For manufacturing, if the sample is expanded to include nonoperational projects, proximity increases recognition only for facilities that are at least partially operational; estimates for the planning/permitting or under construction phases are near zero, although the operational–nonoperational difference is not statistically distinguishable from zero. For renewable projects, proximity only has a detectable positive effect on recognition before construction begins whereas the estimates are positive yet imprecise for already under construction projects, possibly due to publicity around siting decisions or local news coverage (*SI Appendix*,section S3.6).

Respondent partisanship does not consistently moderate proximity’s effect on visibility. Among Republicans, closer proximity to renewable projects causes higher recognition, but the Republican-Democrat contrast is imprecisely estimated, so it is not possible to conclude that proximity has a larger effect on Republicans compared to Democrats. Among Independents, closer proximity to manufacturing projects makes them more visible, yet again the party interaction is only weakly distinguishable from zero. Income and education, which could predict political awareness, also have no consistent moderating effects (*SI Appendix*, section S3.6).

#### Proximity and credit attribution.

There is no evidence that people closer to post-IRA projects are more likely to credit President Biden for these investments (Fig. 2*B*). The point estimates for credit are generally near zero. A post hoc analysis shows that the design has 80% power (α=0.05) to detect a 10-percentage-point effect (*SI Appendix*, section S3.4). Two one-sided equivalence tests further bound any proximity effect to be small; effects larger than 6.7 pp for manufacturing and 6.6 pp for renewables are rejected since the 90% CIs lie entirely within these margins. The analysis cannot rule out more limited political returns, but such a hypothetical effect would be substantively modest relative to the scale and expectations of Biden-era investments.

There is no detectable heterogeneity in proximity’s effects by respondent partisanship. There is also no clear heterogeneity by education, income, project status, or sector (*SI Appendix*, section S3.6).

**Fig. 3.**
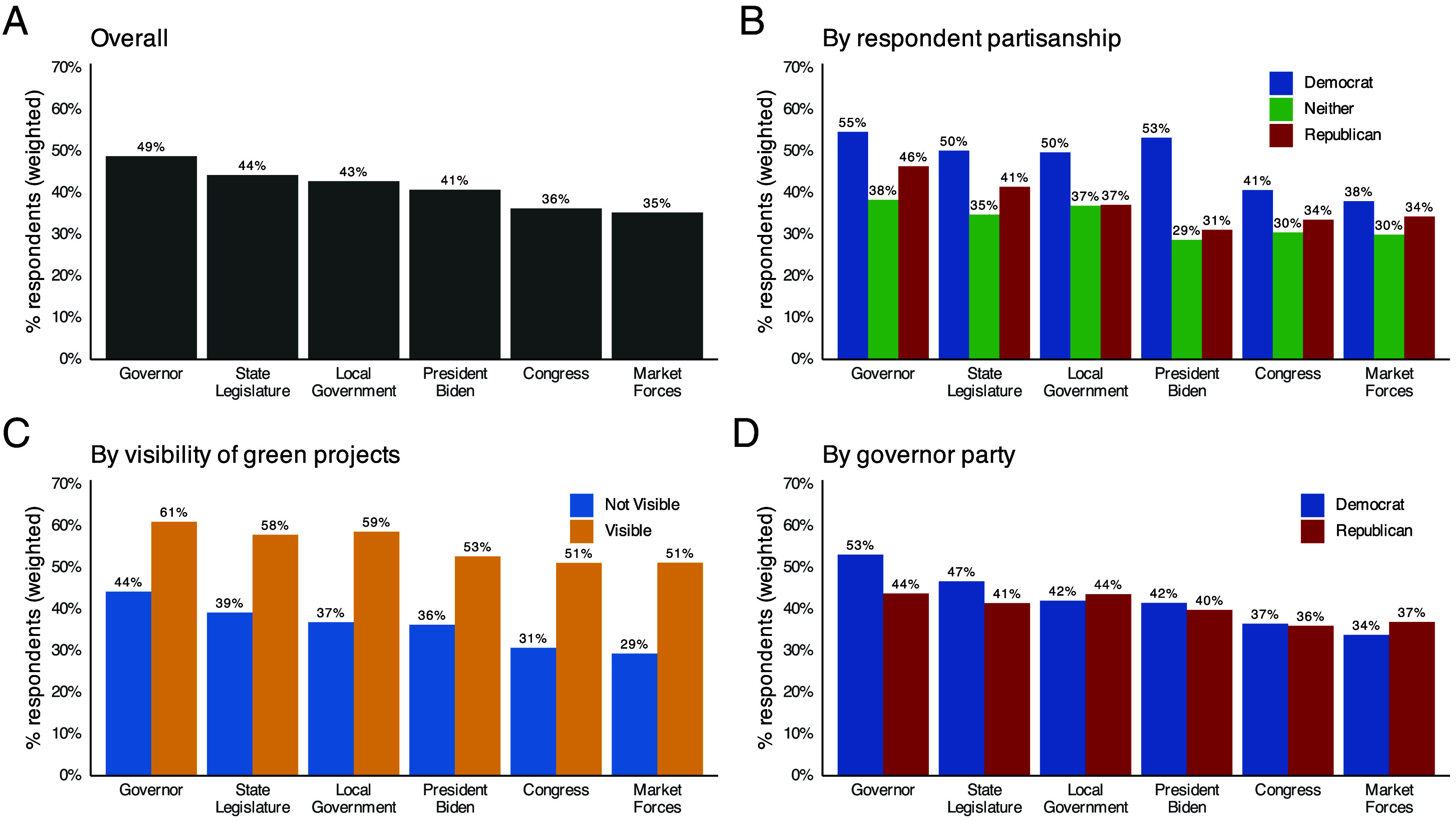
National public beliefs about responsibility for clean energy investments. Plots show the weighted share of respondents who say each actor is responsible for new green investments in their state. Data are from two national samples collected in 2024 (pooled N=3,034). (*A*) Overall. (*B*) By respondent partisanship. (*C*) By self-reported visibility of local green projects. (*D*) By governor party affiliation.

Fig. 3*A* shows that, overall, governors receive the most credit for new clean energy investments. President Biden trails by eight percentage points. Congress and market forces receive the least credit.

Respondents who report having a project in their community are more likely to credit all actors, including Biden, but subjective recognition does not shift responsibility disproportionately toward the Biden Administration (Fig. 3*C*).

The governor’s credit advantage is not driven by Republican respondents who might hesitate to acknowledge Biden’s role. Self-identified Democrats are more likely than Republicans to see their governor as responsible for green projects, although unlike Republicans they also view President Biden as being comparably responsible (Fig. 3*D*). Partisanship still matters: Respondents are more likely to credit their governor when they share the same party (*SI Appendix*, section S5).

### Business and Politician Credit Claiming Patterns.

#### Who “speaks”.

**Fig. 4.**
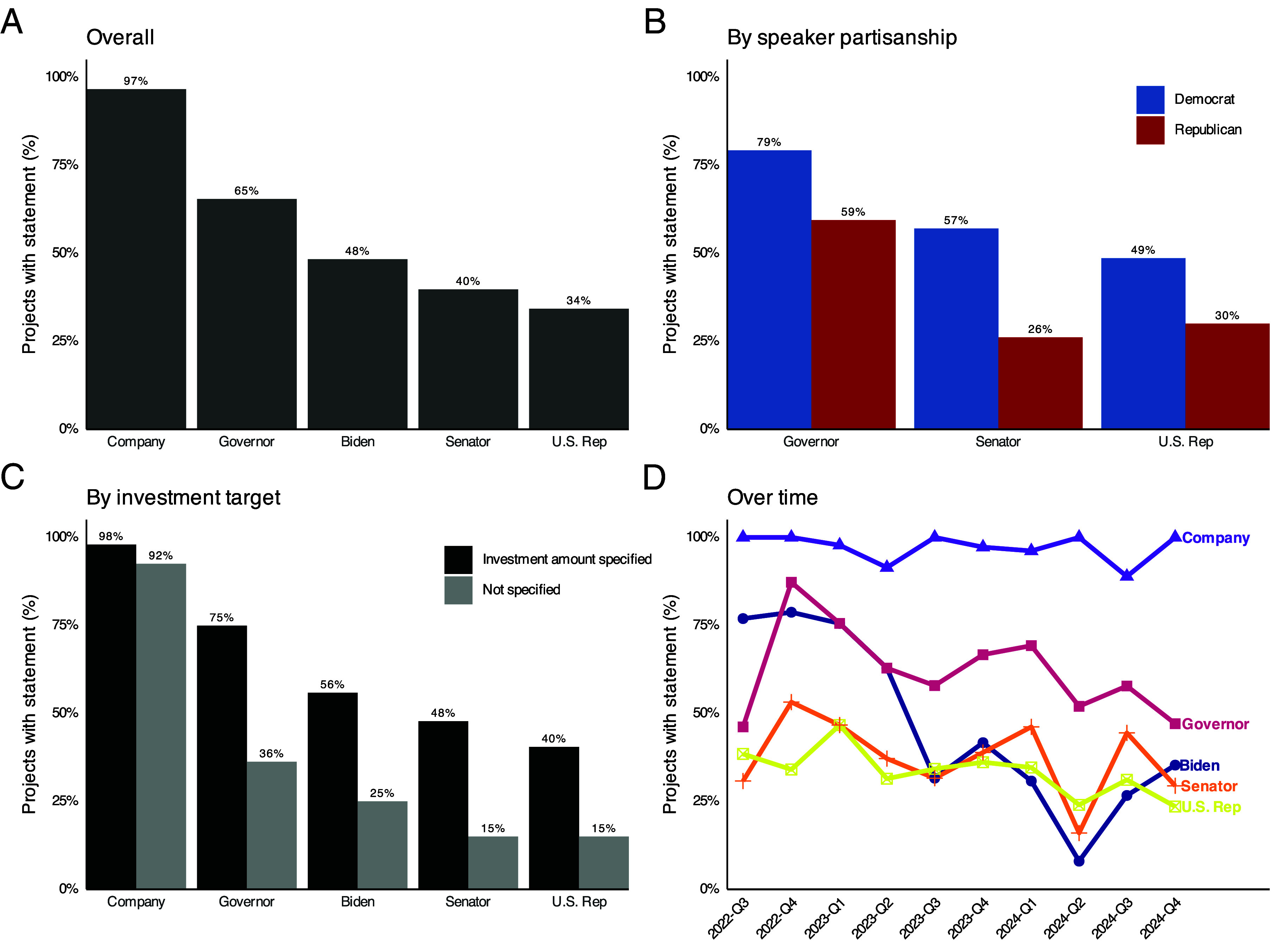
Share of clean energy manufacturing projects with at least one public statement by companies and elected officials after the IRA’s passage (327 projects; Aug. 16, 2022–Dec. 30, 2024). (*A*) Overall. (*B*) By speaker partisanship. (*C*) By investment target. (*D*) Over time.

Companies issue statements for nearly all projects (Fig. 4*A*). Among political actors, governors “speak” most frequently, followed by the Biden Administration, U.S. Senators, and the district’s U.S. Representative. The gap between governors and the White House is 17 percentage points.

Statement rates vary by the speaker’s partisanship (Fig. 4*B*). Democratic governors, senators, and representatives speak more often than Republican counterparts. The partisan gap is largest for members of Congress, although Republican governors comment on 59% of projects in their states. These partisan differences appear even when controlling for project type, construction status, and local economic and political factors (*SI Appendix*, section S6).

Politicians issue more statements about projects that have specific investment amounts (Fig. 4*C*), which is consistent with elected officials having incentives to claim credit for good economic outcomes. These patterns are consistent when examining whether statements contain specific job-creation estimates. For context, about three-quarters of projects report either a capital investment or job-creation target. The association between more concrete economic benefits and politician credit-claiming persists with covariate adjustment for capital investment but not job targets (*SI Appendix*, section S6).

Presidential statements are most frequent immediately after the IRA’s passage in the third quarter of 2022, then decline through mid-2024 and remain below governor levels despite a pre-election uptick (Fig. 4*D*). Governor statement rates are comparatively stable. Politicians claim credit for projects even before the IRA’s full implementation.

Project stage is not statistically associated with the likelihood of statement-giving by any actor (*SI Appendix*, section S6.5). Statement frequency does not differ across planning, construction, operation, or termination stages. Politicians issue statements throughout the project life-cycle, from initial announcement through completion.

#### Who credits whom.

**Fig. 5.**
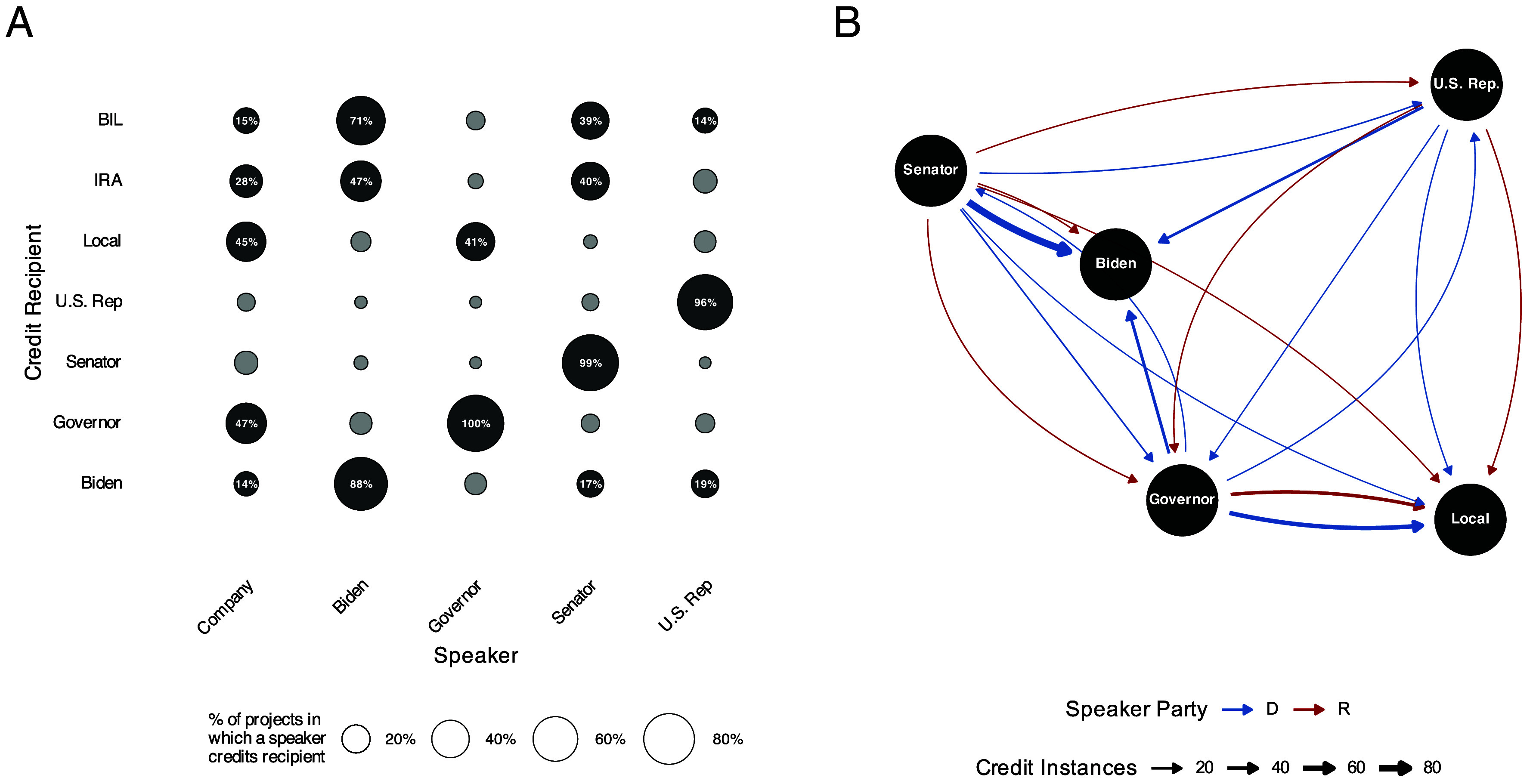
Credit giving for clean energy manufacturing projects after the IRA’s passage (327 projects; Aug. 16, 2022–Dec. 30, 2024). (*A*) Share of projects in which each speaker credited each recipient. (*B*) Project-level credit network by speaker partisanship. Edge thickness denotes the share of projects. Blue lines denote Democratic speakers and red Republicans.

Fig. 5*A* shows the share of projects in which a speaker’s statement credits each recipient. Companies spread credit broadly. Businesses most often credit governors and local actors, followed by the IRA, BIL, and President Biden.

Across elected officials, self-credit is unsurprisingly common. When President Biden and his delegates speak, they reference the BIL in 71% of projects and the IRA in 47%. Biden and his administration’s officials occasionally credit governors and local officials.

Federal legislators do not issue as many statements as governors, but when they do, Senators credit the White House 17% of the time and Representatives credit the Biden Administration in 19% statements. Federal lawmakers also sometimes acknowledge governors and local politicians.

Fig. 5*B* visualizes project-level credit networks varying with the speaker’s partisanship. Governors from both parties recognize local actors. Democratic governors occasionally credit President Biden while Republican governors never do. Republican speakers, compared to Democrats, are less likely to credit the White House, a pattern that holds with covariate adjustment (*SI Appendix*, section S6).

Whether a project is operational is not associated with a speaker’s propensity to credit the Biden Administration (*SI Appendix*, section S6). By contrast, attribution to other political actors varies by project status: Companies are more likely to acknowledge governors, U.S. Senators, and U.S. Representatives during pilot, planning, or construction phases than for projects that are later mothballed.

## Discussion

Federal policymakers intended for clean energy investments not only to address climate change but also to generate political returns from voters in communities receiving new projects. While proximity modestly makes projects more visible, it does not increase credit to the Biden Administration, which enacted policies to support renewable electricity and clean manufacturing. Less than half of Americans (41%) view President Biden as responsible, whereas governors receive the most credit from across the political spectrum. Biden-era green investments are visible, but not traceable to the federal government.

The mixed information environment offers a plausible explanation for why the Biden Administration receives relatively limited credit. Governors from both parties issue more statements about green manufacturing projects than the White House. Presidential messaging is most frequent immediately after the IRA’s passage and declines over time, whereas governors issue more consistent messages. Companies spread credit across multiple actors, highlighting state and local partners more often than President Biden and federal legislation like the IRA and BIL. The supply of competing messages emphasizes subnational actors over federal ones, diluting policy traceability.

While we cannot observe the internal strategies of firms, three considerations may help interpret why companies distribute credit broadly. First, firms manage political risk over long horizons, so spreading credit could preserve relationships with multiple governments (17). Second, state and local governments provide tangible support for projects such as incentives, infrastructure, and permitting, so credit is often warranted (18). Third, companies often need a broad coalition—federal, state, local, labor, and civic leaders—to bring a project to fruition. Threading this needle, especially in red districts, might require remaining silent about blue politics and focusing instead on local benefits, a hypothesis that future research should explore.

While we cannot observe why the Biden Administration did not more actively claim credit, we speculate that the administration was constrained by the concern that presidential branding could polarize projects in purple and red states. Suggestive of this motive is that when the president spoke, his statements more frequently credited the BIL than the IRA, which passed on a party-line vote. The White House was also at a structural messaging disadvantage compared to governors who have more time to visit projects.

The public opinion dynamics we study are a prerequisite for green investments to affect voting behavior. If voters do not credit the responsible policymakers for outcomes they see as positive, they cannot reward politicians’ efforts at the ballot box. Since elections involve multiple competing issues, new clean energy projects may not outweigh the electorate’s other concerns (19). In some cases, however, the material benefits such as new jobs and tax revenue could lead people to vote based on their self-interest (20). Clean energy investments have the potential to deliver tangible benefits: In our survey, 66% of the public view green projects as economically beneficial, including 50% of Republicans and 81% of Democrats, mirroring polls that show bipartisan clean energy support (21, 22). Traceability is a necessary but not sufficient condition for political returns.

Several limitations qualify our conclusions and chart research priorities. Although the national samples had adequate coverage near projects, it is possible that feedback effects would be stronger in places even more proximate to investments and among people receiving jobs. The next wave of work could conduct geotargeted samples.

The samples are also cross-sectional, which carries inherent limitations. More frequent survey waves would allow researchers to better parse the effect of presidential messaging, which was more active immediately after the IRA’s passage, although the true test of feedback effects is whether public opinion durably changes even as messaging fades. Additionally, the samples capture public attitudes two years post-IRA and, despite our analysis of operational projects, some investments may have delayed effects. Researchers should field follow-up surveys, especially in places with project cancellations due to the IRA’s partial repeal.

Another limitation is that the responsibility survey question is framed at the state level, which may favor governors, but this wording improves comparability and matches how projects were presented locally. Future surveys should ask about the role played by additional actors, such as the Democratic and Republican parties and the respondent’s U.S. Representative, in addition to beliefs about responsibility for canceled or delayed green investments.

The research design also cannot estimate the causal effect of message supply on attribution, nor how much of a difference more active presidential messaging could have made, because statements are not random but follow political strategy. Answering these questions will require experiments that randomize politicians’ communication strategies. Previous research shows that such legislator communication is influential in constituent perceptions of credit for positive policy outcomes (23).

The findings align with research on how visibility and traceability shape whether public policy affects mass attitudes (7). Clean energy investments may face particular barriers compared to other government policies. First, the economic benefits from clean energy and manufacturing incentives materialize indirectly through private firms rather than directly through voter contact with government agencies as with Social Security (24). Reliance on businesses also extends the lag between a law’s passage and its visible effects due to such factors as siting delays, obscuring the government’s hand in clean energy projects. Second, national, state, and local policies influence clean energy projects, complicating attributions in federal systems (25). The public has limited knowledge about what goes on in Washington, further complicating voter judgments of responsibility (26). Politicians cannot count on “good” policy alone to deliver political returns.

Biden-era green investments due to their scale may be a most-likely policy feedback case compared to previous research examining earlier wind turbine construction (27). The IRA also contained other types of green spending that could be more traceable, e.g. credits targeted at individuals to install heat pumps or purchase electric vehicles. There is mixed evidence from carbon pricing research about whether such direct benefits are visible to consumers (28). Yet heat pumps and EVs are more concrete than end-of-year tax rebates. Researchers should investigate how different types of green spending and ways of delivering material benefits affect whether climate policy feedbacks take root.

If green investments can create feedback effects, our findings suggest that they are more likely to operate through organized interest groups than voters (4, 6, 29). Businesses can better link policy changes to their economic interests and have strong incentives to lobby when benefits are concentrated (30). Scholars should study how green investments affect both lawmakers and interest groups, whose strategies may also be constrained by public opinion (19, 31).

Challenges to federal climate policy will evolve over time. The limited ability of federally backed green investments to create public supporters poses a serious hurdle. The IRA’s partial repeal in 2025 resulted from multiple forces beyond public opinion, including partisan polarization (32, 33). Still, Republican legislators might have faced stronger pressure to resist cutbacks had constituents been more aware of the IRA’s role in creating local benefits. At the same time, cuts to green investments could carry political costs, as canceled projects may prove more effective at mobilizing beneficiaries (34). Yet the core constraint remains: For climate spending to create political returns, the public must be able to trace material benefits back to federal policymakers.

## Materials and Methods

### Survey Data and Measurement.

#### Sampling.

Three nonprobability national online surveys of U.S. adults were administered via Qualtrics in 2024. Surveys were available in English. Respondents consented to participate at the survey’s start. The study was approved by Harvard University IRB (#17-1328). Fieldwork periods were March 14–April 9 (N=1,500), May 13–June 6 (N=1,992), and August 6–November 11 (N=1,534). After applying the data quality protocol (attention checks; speeding; duplicate IP/device; invisible reCAPTCHA), the combined sample includes 5,026 respondents. Samples used quotas to approximate the U.S. adult population by age, sex, race/ethnicity, education, income, and region, based on the 2023 5-y ACS.

#### Measures.

All samples included a visibility item: “In the last year, have there been any clean energy investments in your community? Examples include wind and solar farms, and plants to build electric cars or batteries.” Response options were “Yes,” “No,” or “Not sure.” Analyses coded recognition as a binary indicator 1 for yes, and 0 otherwise.

Samples 1 and 3 included an attribution battery: “Thinking about your state, who or what has played a significant role in bringing clean energy investments? For each option, please rate how responsible you believe they are.” Respondents rated President Biden, the U.S. Congress, their governor, state legislature, community leaders, and market forces (randomized order) on a five-point scale: extremely, very, moderately, not too, or not at all responsible. We used the term “responsibility” rather than “credit” to maximize construct validity, since “credit” can have a positive connotation. Analyses use a binary indicator coded 1 for “Extremely” or “Very” and 0 otherwise. Diagnostic checks show that the question captured the principal perceived sources of responsibility and engaged respondents similarly across partisan groups (*SI Appendix*, section S2.4). The state-level frame ensured that the item was meaningful for all respondents and reflected how the IRA’s design emphasized place-based benefits.

The visibility item always preceded the attribution battery to minimize priming of recognition by political responsibility. The location of other survey content, such as demographics, varied by sample.

#### Geolocation and linkage.

Respondents are geocoded to ZIP Code centroids, the most granular geographic identifier available, and linked to the nearest project in the two years before the survey date. The two-year window captures the relevant post-IRA period when attribution is most likely and provides more variation in proximity to projects to test the hypotheses; results are robust to a one-year window (*SI Appendix*, section S3.5). ZIP Codes are self-reported and mapped to longitude and latitude coordinates using the Google Maps API. IP-based geolocation is used when a reliable ZIP Code is unavailable (<1%). Results are robust to restricting the sample to respondents whose ZIP Code coordinates match those implied by IP addresses (*SI Appendix*, section S3.5).

#### Weights.

Descriptive estimates use survey weights. Proximity regressions do not, but weighted regressions are similar (*SI Appendix*, section S3.5). Although the raw samples generally approximated the national population, validation checks show that the weights improve representativeness (*SI Appendix*, section S2.2).

### Clean Energy Project Data.

Utility-scale solar and wind generation projects were identified from the U.S. Energy Information Administration’s EIA-860M monthly generator updates. The IRA directly incentivized these technologies through the §48E Investment Tax Credit (ITC) and §45Y Production Tax Credit (PTC) for clean electricity; the BIL primarily had an indirect effect on these investments through investments in transmission and grid resilience. Project locations were defined using longitude/latitude coordinates. A plant is included if at least one solar or wind generator at the facility was reported as preconstruction or under construction, with a start time within the two years preceding the respondent’s survey date. Records with invalid coordinates were excluded.

Green manufacturing plant information comes from Jay Turner’s Big Green Machine dataset (April 19, 2025, version), which is compiled from public sources and tracks EVs, batteries, solar, and wind. The IRA incentivized these projects through the §45X Advanced Manufacturing Production Credit, the §30D clean-vehicle credit, domestic-content bonus for the ITC and PTC, and DOE Loan Program financing; BIL §40207 established grants and demonstration projects for battery materials processing, manufacturing, and recycling. The proximity analysis sample excludes rumored, closed, or canceled projects; records lacking an announcement date or valid geocoordinates; and it includes only facilities operational or partly operational within two years before the respondent’s interview date and after the IRA’s passage. The statement analysis considers all post-IRA manufacturing projects regardless of operational status.

### Company and Politician Statements.

#### Collection.

The statement dataset covers 327 manufacturing projects. It tracks public statements by companies, governors, U.S. Senators, U.S. Representatives, and President Biden. The collection window spans August 16, 2022, to December 31, 2024. The research team located 992 statements out of 1962 potential statements.

A statement is defined broadly to minimize false negatives. It includes i) official communications (press releases, newsletters, transcripts, reports) published on government or corporate websites; ii) posts on verified social media accounts including Facebook, X/Twitter, Instagram, and LinkedIn; and iii) direct quotes attributable to the actor in credible news articles or in another actor’s press release. Multiple distinct statements by one actor about the same project were consolidated into one record. When a company press release contained a politician’s quote and no separate official statement existed, that quote was used as the politician’s statement and the company release is cited.

#### Annotation.

Statements were annotated to identify i) whether they contained a credit claim and ii) the recipient(s) of credit. Potential recipients included President Biden, the state’s U.S. Senator(s), the district’s U.S. Representative, the governor, local officials, the IRA, and the BIL; party brands (e.g., Democratic Party) were also checked but were almost never credited.

The annotation protocol used LLMs. GPT models can match or exceed crowd workers on common text-as-data tasks, often with higher intercoder agreement and lower cost (35), including political science applications (36, 37). The annotation protocol followed emerging best-practices, including codebook prompts, temperature control, model disclosure, human calibration, and postprocessing, which increase reproducibility (38).

A two-stage LLM-assisted procedure was used. Stage 1 (policy targeting) applied gpt-3.5-turbo-0125 at temperature 0 to classify whether the statement explicitly indicated that the IRA or BIL funded, financed, or enabled the specific project. Stage 2 (general credit) applied gpt-4o-mini at temperature 0 using the full codebook to identify credit claims and assign recipients. The Stage-2 prompt included: a) the statement text; b) statement metadata (speaker/company, role, state/district, channel, release type); and c) Stage-1 outputs as features.

The codebook captured explicit credit (e.g., causal verbs, attributions of decision-making, financial involvement) and implicit credit (e.g., attending or hosting a project ceremony, framing an announcement as an achievement, public association with a specific project using active language). Post-processing ensured that a statement could be coded as crediting the IRA/BIL/President only if a corresponding synonym appeared in text. Human coders and the LLMs jointly annotated a prompt development subset of two statements for every actor to refine instructions. Results are qualitatively similar with an alternative codebook with a more permissive credit definition.

We validated LLM annotations against an independent human coder using a blinded RA and a stratified random sample of 100 statements (39). Performance was high for credit-claiming (F1 = 0.92), crediting President Biden (F1 = 0.96), and crediting governors (F1 = 0.74), with agreement rates of 88 to 98%. While precision is lower for governor credit, validation tests show no statistically significant bias, and even upper-bound corrections do not affect our substantive conclusions. Full diagnostics and bias-adjusted estimates for credit-claiming regression models are reported in *SI Appendix* section S6.8.

### Analyses.

#### Causal identification.

The analysis estimates the effect of project proximity on visibility and credit attribution. Since project location could be confounded by political and economic factors, the research design leverages within-state variation in proximity. The assumption is that the within-state deviation in distance to projects is as-if random after controlling for individual and county-level covariates that predict site selection within a state. The centrality of state-level factors for project site selection, which the state fixed effects address, bolsters the credibility of this assumption.

The analysis includes pretreatment county and individual-level covariates. County-level controls include the unemployment rate, labor force size, county domestic product, median income per capita, highway access, share of college-educated residents, share of residents under the federal poverty line, share of foreign-born residents, median housing costs, population density, broadband access, and 2020 Biden vote share. Controls are lagged by a year where applicable. Individual-level controls include age, sex, race, education, labor force participation, income, party identification, and global warming beliefs.

#### Estimation.

The analysis uses a linear probability model with the following specification:

$$\begin{array}{c} Y_{i}=\sum_{q=1}^{4}1\left\{Distance_{i}\in Q_{q}\right\}\beta_{q}+X_{i}^{⊤}\gamma +State_{i}+Sample_{i}+\epsilon_{i}, \end{array}$$

where $Q_{5}$ (farthest quintile) is the reference. The primary analysis operationalizes distance with quintiles to ensure that it captures nonlinear effects. Outcomes are indicators for visibility and credit to President Biden. $X_{i}$ includes individual- and county-level covariates; State and Sample denote state and sample fixed effects.

An omnibus Wald test assesses the joint null that the first two distance quintile indicators equal zero. For the visibility outcome, the test rejects the null of no proximity effect for renewables (P=0.01), whereas the pooled contrast for manufacturing is smaller and not distinguishable from zero (P=0.095).

#### Inference.

Spatial HAC (Conley) SEs were computed using respondents’ ZIP-centroid latitude/longitude, a uniform kernel with a 50 km cutoff. Results are robust to computing great-circle distances, to alternative cutoffs, and to clustering by state (*SI Appendix*, section S3.5).

#### Sensitivity.

The sensitivity analysis quantifies the strength of a hypothetical unobserved confounder required to reduce the Q1 proximity coefficient to insignificance at the 5% level (40). A hypothetical omitted variable would need to be much larger than the correlations of strong observed predictors of both proximity and the visibility outcome (*SI Appendix*, section S3.5).

## Supplementary Material

Appendix 01 (PDF)

## Data Availability

All nonidentifying data, code, and materials required to reproduce the results in this article are archived with the Harvard Dataverse (41); precise geographic coordinates are excluded from the public archive to protect respondent privacy and are available from the authors under a data sharing agreement.
