# Supplementary material for: Why Biden-era clean energy investment policies had limited political returns
Source: Proc Natl Acad Sci U S A. 2026 Feb 26;123(9):e2526802123. doi: 10.1073/pnas.2526802123 (PMC12956879; doi:10.1073/pnas.2526802123)
Supplement: Supplementary file 1 — Appendix 01 (PDF) [file pnas.2526802123.sapp.pdf]

SI Appendix

Why Biden-Era Clean Energy Investment Policies Had Limited Political Returns

S1 Data Sources **S3**

S2 Survey **S4**
S2.1 Sample Summary Statistics . . . S4
S2.2 Weight Diagnostics . . . S5
S2.3 Survey Instrument . . . S6
S2.3.1 Background Characteristics . . . S6
S2.3.2 Climate Change Beliefs . . . S6
S2.3.3 Political Background . . . S7
S2.4 Credit Attribution Item Internal Validity . . . S8

S3 Proximity Effect Analyses **S9**
S3.1 Summary Statistics . . . S9
S3.2 Within-State Variation in Distance . . . S10
S3.3 Main Regression Table . . . S11
S3.4 Power Analyses . . . S12
S3.5 Robustness Checks . . . S14
S3.5.1 Sensitivity to Omitted Variable Bias . . . S14
S3.5.2 Alternative Standard Errors . . . S15
S3.5.3 Alternative Geocoordinates . . . S17
S3.5.4 Continuous Distance Measure . . . S19
S3.5.5 Weighted Regressions . . . S20
S3.5.6 Media Market Fixed Effects . . . S21
S3.5.7 Additional Credit Recipients . . . S22
S3.5.8 One-Year Temporal Window . . . S23
S3.6 Treatment Effect Heterogeneity . . . S24
S3.6.1 Manufacturing Operational Status . . . S25
S3.6.2 Renewable Energy Operational Status . . . S27
S3.6.3 Renewable Energy Technology . . . S29
S3.6.4 Manufacturing Sector . . . S31
S3.6.5 Partisanship . . . S34
S3.6.6 Education . . . S37
S3.6.7 Household Income . . . S39
S3.6.8 Biden Election Exit . . . S41

S4 Regression of Perceived Benefits on Covariates **S44**

S5 Regression of Credit Attribution on Covariates **S47**

S6 Statement Analyses **S49**
S6.1 Statement Type Description . . . S49
S6.2 Summary Statistics . . . S50
S6.3 LLM Annotation . . . S51
S6.3.1 Stage 1 Prompt . . . S51

S6.3.2 Stage 2 Prompt S51
S6.4 Robustness to Alternative Codebook S54
S6.5 Regression Models of Statement Giving S55
S6.6 Regression Models of Statement Credit Attribution S56
S6.7 By Target Jobs S60
S6.8 Annotation Quality Analysis and Robustness S61

S7 Investment Timing **S65**
S7.1 Clean Electricity Incentives S65
S7.2 Clean Manufacturing Incentives S66

S1 Data Sources

Table S1: Data Sources

Variable	Source and Description	Access Link
Clean Energy Data		
Clean energy: generation	EIA-860M (monthly). Reports existing and proposed generating units ≥1 MW; required reporting for new generators scheduled within 12 months of operation.	EIA-860M
Clean energy: manufacturing	The Big Green Machine dataset (Wellesley College) covering North American clean-energy supply chains from extraction to manufacturing.	Big Green Machine
Electricity prices (industrial)	State-level average industrial electricity price, 2023 (EIA Table 5C), cents per kWh.	EIA: Sales/Revenue/Price
Political Actors & Elections		
Democratic vote share (2020)	David Leip's Atlas of U.S. Presidential Elections. Alaska reports by state house district; converted to counties via population-weighted harmonization using district and county shapefiles.	US Election Atlas
Governor party	Ballotpedia state executive data (incumbent party at survey reference date).	Ballotpedia
Lawmaker parties	Official rosters from the Senate and House Clerks (used to assign party of state's federal delegation).	Senate Clerk<br>House Clerk
Congressional elections	MIT Election Data + Science Lab.	Dataverse
Economic Context		
Unionization rates (private sector)	State-level union coverage/intensity, 2023; series based on Hirsch and MacPherson (2003).	UnionStats
Broadband access	FCC Form 477 county-level Internet Access Services (Tier 4: residential fixed ≥100 Mbps downstream).	FCC Form 477
Unemployment rate	Annual average county-level unemployment (BLS Local Area Unemployment Statistics).	BLS LAU Tables
Labor force size	Annual average county-level labor force (BLS LAU).	BLS LAU Tables
Gross domestic product	County real GDP, chained dollars, all industries (BEA CAGDP9).	BEA: GDP by County
Per capita income	County personal income per capita (BEA CAINC30).	BEA Regional Data
Highway access	TIGER/Line shapefiles (U.S. Primary Roads, 2023). Interstate access coded as a binary based on county–interstate intersection.	TIGER/Line: Primary Roads
College degree share	ACS 2023 5-year estimate, share of residents with BA+ (table B06009_005).	Census API (ACS 5-year)
Poverty rate	ACS 2023 5-year estimate, below poverty (table B06012_002).	Census API (ACS 5-year)
Median housing costs	ACS 2023 5-year estimate, median monthly housing costs (table B25105_001).	Census API (ACS 5-year)
Foreign-born share	ACS 2023 5-year estimate, foreign-born (table B06012_017).	Census API (ACS 5-year)
Population density	Derived from 1 km WorldPop raster aggregated to 25 km circles around each respondent's lat–lon.	WorldPop Hub

S2 Survey

S2.1 Sample Summary Statistics

Table S2: Survey sample summaries

	2024 Field Date		
	3/14–4/9	5/13–6/6	8/6–11/11
Age	47	49	49
Female	0.54	0.52	0.52
Black	0.14	0.14	0.13
Asian	0.043	0.057	0.055
Other race	0.072	0.087	0.067
Hispanic/Latino	0.19	0.18	0.18
College	0.37	0.35	0.35
Employed	0.58	0.53	0.53
Income Q1	0.22	0.22	0.22
Income Q2	0.26	0.24	0.24
Income Q3	0.27	0.28	0.27
Income Q4	0.16	0.16	0.17
Income Q5	0.091	0.098	0.093
Democrat	0.44	0.45	0.47
Republican	0.39	0.37	0.37
Global Warming Index	0.76	0.75	0.76
N	1500	1992	1534

Notes: Prefer not to say income answers imputed with median household income. Column header shows month/day of survey wave start and end dates. Global warming index ranges from 0 to 1, where larger values indicate greater concern.

S2.2 Weight Diagnostics

Survey weights were constructed for the pooled sample and separately for questions only on specific samples. Iterative proportional fitting (raking) was used to align the sample to population benchmarks from the 2023 ACS 5-year release. The raking targets included the joint distribution of gender × age × education, and the marginal distributions of race/ethnicity, household income, and Census region (4 categories). Weights were trimmed to the interval [0.3, 3.0] to limit the influence of extreme values and were normalized to have mean 1 within each analysis sample.

Table S3: Comparison of Survey Distributions with ACS Population Benchmarks

Demographic Category	Unweighted	Weighted	ACS Target	Abs Diff (W-ACS)	Abs Diff (U-ACS)
Race: Asian Alone	0.05	0.06	0.06	0.00	0.01
Race: Black or African American Alone	0.13	0.12	0.12	0.00	0.01
Race: Other	0.08	0.15	0.16	0.02	0.08
Race: White Alone	0.74	0.67	0.66	0.02	0.08
Income: Q1	0.22	0.19	0.18	0.01	0.04
Income: Q2	0.25	0.21	0.20	0.01	0.04
Income: Q3	0.27	0.23	0.22	0.01	0.05
Income: Q4	0.16	0.18	0.17	0.00	0.01
Income: Q5	0.09	0.19	0.22	0.02	0.12
Region: Midwest	0.21	0.21	0.20	0.00	0.00
Region: Northeast	0.18	0.17	0.17	0.00	0.01
Region: South	0.38	0.39	0.39	0.00	0.01
Region: West	0.23	0.23	0.23	0.00	0.00
18-24 × No College × Female	0.05	0.05	0.05	0.00	0.00
25-34 × No College × Female	0.08	0.05	0.05	0.00	0.03
35-44 × No College × Female	0.04	0.05	0.05	0.00	0.00
45-64 × No College × Female	0.10	0.11	0.11	0.00	0.00
65+ × No College × Female	0.09	0.09	0.09	0.00	0.00
18-24 × College × Female	0.01	0.01	0.01	0.00	0.00
25-34 × College × Female	0.03	0.04	0.04	0.00	0.01
35-44 × College × Female	0.02	0.03	0.04	0.00	0.01
45-64 × College × Female	0.04	0.06	0.06	0.00	0.01
65+ × College × Female	0.06	0.03	0.03	0.00	0.03
18-24 × No College × Male	0.03	0.05	0.05	0.00	0.02
25-34 × No College × Male	0.06	0.06	0.06	0.00	0.00
35-44 × No College × Male	0.05	0.05	0.05	0.00	0.00
45-64 × No College × Male	0.07	0.10	0.11	0.00	0.04
65+ × No College × Male	0.06	0.06	0.06	0.00	0.01
18-24 × College × Male	0.00	0.01	0.01	0.00	0.00
25-34 × College × Male	0.04	0.03	0.03	0.00	0.01
35-44 × College × Male	0.05	0.03	0.03	0.00	0.02
45-64 × College × Male	0.04	0.05	0.05	0.00	0.01
65+ × College × Male	0.06	0.03	0.03	0.00	0.02

S2.3 Survey Instrument

The questions below were used in the analysis and were not already described in Materials and Methods. The question order varies slightly across the samples.

S2.3.1 Background Characteristics

1. Are you male or female?

   Male; Female

2. Are you Spanish, Hispanic, or Latino or none of these?

   Yes; None of these

3. Choose one or more races that you consider yourself to be:

   White; Black or African American; American Indian or Alaska Native; Asian; Native Hawaiian or Pacific Islander; Other

4. In what year were you born? (text entry)

5. What is your state? (drop-down list)

6. What is the highest level of education you have completed?

   No high school; Some high school; High school diploma or GED; Some college course work but non-degree certificate; Technical certificate; Associate degree; Bachelor's degree; Advanced degree (post college, such as JD or MBA)

7. What is your 5 digit ZIP code? (text entry)

S2.3.2 Climate Change Beliefs

8. Climate change refers to the claim that the world's average temperature has been increasing over the past 150 years, may be increasing more in the future, and that the world's climate may change as a result.

   What do you think? Do you think that climate change is happening?

   Climate change is happening; Climate change is not happening

9. How sure are you that [pipe in answer from the previous question]?

   Very sure; Somewhat sure; Not sure

10. Which of the following statements comes closest to your own opinion?

    Humans are causing climate change; Humans are not causing climate change

11. How sure are you that [pipe in answer from the previous question]?

    Very sure; Somewhat sure; Not sure

12. Which of the following do you think best describes your view about global warming?

    This is not a serious problem; More research is needed before action is taken; We should take some action now; Immediate and drastic action is necessary

13. How would you describe your current employment status?

    Employed full-time; Employed part-time; Work in the home (not paid); Not employed, but looking for work; Not employed, and not looking for work

14. Thinking back over the last year, what was your family's annual income?

    Less than $10,000; $10,000 - $19,999; $20,000 - $29,999; $30,000 - $39,999; $40,000 - $49,999; $50,000 - $59,999; $60,000 - $69,999; $70,000 - $79,999; $80,000 - $99,999; $100,000 - $119,999; $120,000 - $149,999; $150,000 - $199,999; $200,000 - $249,999; $250,000 - $349,999; $350,000 - $499,999; $500,000 or more; Prefer not to say

S2.3.3 Political Background

15. Generally speaking, do you think of yourself as a...?

    Democrat; Republican; Independent; Other (text entry)

16. (If Democrat/Republican) Would you call yourself a strong [Democrat/Republican] or not so strong [Democrat/Republican]?

    Strong [Democrat/Republican]; Not so strong [Democrat/Republican]

17. (If Independent or Other) Do you think of yourself as closer to the Democratic or Republican party?

    The Democratic Party; The Republican Party; Neither; Not sure

S2.4 Credit Attribution Item Internal Validity

We took three steps to validate the credit attribution item's accuracy and reliability. First, to minimize partisan differences in response patterns, the question used neutral language to describe green investments; it didn't presume that projects were good or bad. Partisan expressive responding is an inherent risk. As a test, we assess whether the wording disengaged or primed partisans differently. Response times do not differ across partisan identification or ideology (Table S4), suggesting the question was equally engaging across groups.

Table S4: Linear regression model of credit attribution question time latency

	(1)	(2)	(3)	(4)
Intercept	36.4***	37.1***	33.17***	33.9***
	(2.6)	(3.4)	(0.99)	(1.6)
Republican	-2.0	-2.0		
	(2.8)	(2.9)		
Neither party	-3.3	-3.3		
	(3.1)	(3.2)		
Ideology: Conservative			3.1	3.2
			(2.3)	(2.3)
Ideology: Not sure			-3.6	-3.7
			(2.2)	(2.2)
Ideology: Liberal			4.0	4.1
			(4.1)	(4.1)
N	3034	3034	3034	3034
Sample Fixed Effects	No	Yes	No	Yes

Notes: Unit of analysis is the individual survey respondent. Dependent variable: time (seconds) spent on credit attribution survey question. Estimates are OLS with heteroskedasticity-robust standard errors in parentheses. * $p < 0.05$, ** $p < 0.01$, *** $p < 0.001$.

Second, the question asked respondents to evaluate multiple actors independently. This approach avoids forcing trade-offs, as in a bipolar scale (e.g., Biden vs. governor), or imposing unrealistic quantitative judgments, as in a "divide-the-dollar" measure. Nearly all respondents found at least one actor responsible: only 1.2% rated every listed factor as "not at all responsible," suggesting good coverage of perceived sources of responsibility.

Third, we examined potential satisficing through straight-lining (e.g., rating all actors as "extremely responsible"). Such patterns were rare: only 3.2% of respondents did so. This indicates that indiscriminate responding was infrequent.

Taken together, these checks provide evidence consistent with the internal validity and reliability of the credit attribution battery.

S3 Proximity Effect Analyses

S3.1 Summary Statistics

Table S5: Proximity analysis summary statistics

	Mean	SD	Min	Max	NA
Sees clean energy project	0.26	0.44	0	1	0
Credits Biden†	0.42	0.49	0	1	0
Credits State†	0.44	0.5	0	1	0
Credits Congress†	0.36	0.48	0	1	0
Credits Local Officials†	0.42	0.49	0	1	0
Credits Markets†	0.35	0.48	0	1	0
Age	48	18	18	97	0
Female	0.53	0.5	0	1	0
Black	0.14	0.35	0	1	0
Asian	0.052	0.22	0	1	0
Other race	0.077	0.27	0	1	0
Hispanic/Latino	0.18	0.39	0	1	0
College	0.36	0.48	0	1	0
Employed	0.55	0.5	0	1	0
Income Q1	0.22	0.41	0	1	0
Income Q2	0.25	0.43	0	1	0
Income Q3	0.27	0.45	0	1	0
Income Q4	0.16	0.37	0	1	0
Income Q5	0.094	0.29	0	1	0
Democrat	0.46	0.5	0	1	0
Republican	0.38	0.48	0	1	0
Global warming index	0.75	0.3	0	1	0
Unemployment rate	3.8	1	1.7	18	0
Labor force (log) $(t-1)$	12	1.6	6.8	15	0
County GDP (log) $(t-1)$	17	1.8	11	21	0
County income pc $(t-1)$	42349	17735	12744	131902	0
Highway access	0.87	0.34	0	1	0
County college share $(t-1)$	0.34	0.11	0.057	0.66	0
County poverty share $(t-1)$	0.18	0.066	0.03	0.6	0
Median county housing costs $(t-1)$	1414	478	393	3049	0
County foreign-born share $(t-1)$	0.2	0.15	0	0.75	0
Population density	737	1079	0.21	5677	0
Faster broadband access	0.74	0.44	0	1	0
County 2020 Biden vote share	52	17	8.6	92	0

Notes: Summary statistics across all survey samples ($N = 5026$). Analyses standardize continuous county-level measures with the within-state variance. †Credit attribution questions asked only in two of three survey waves ($N = 3034$).

S3.2 Within-State Variation in Distance

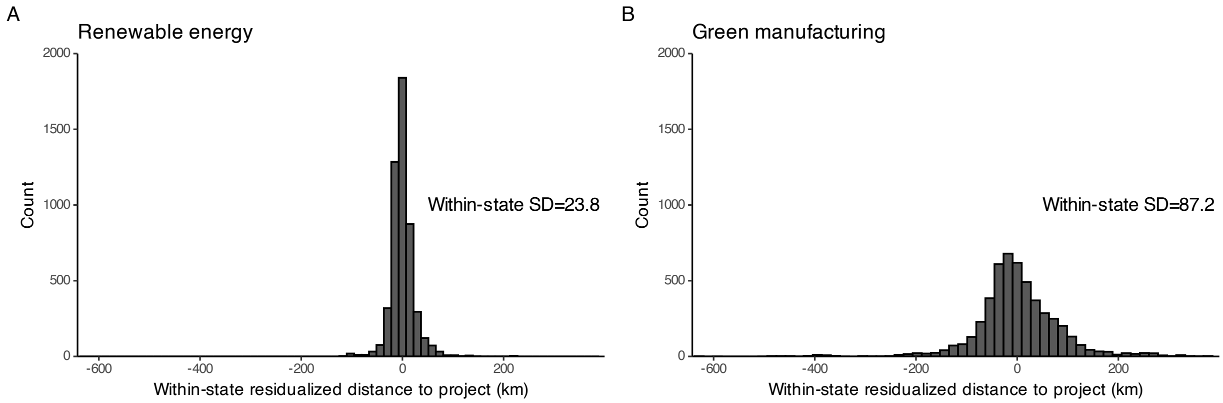

Fig. S1: Within-state variation in survey respondent proximity to clean energy investments. Histograms plot the residual variation in continuous (logged) distance to renewable energy and green manufacturing projects after regressing the distance measures on the state fixed effects, sample fixed effects, and covariates in the main model specification.

S3.3 Main Regression Table

Table S6: Linear probability models of proximity's effect on project visibility, credit attribution, and perceived benefits

	Visibility (=1)		Credit Biden (=1)		Benefits (=1)	
	Renewable Energy	Manufacturing	Renewable Energy	Manufacturing	Renewable Energy	Manufacturing
Q1 proximity	0.066**	0.063*	-0.013	0.005	0.0026	-0.013
	(0.022)	(0.029)	(0.031)	(0.037)	(0.0441)	(0.046)
Q2 proximity	0.045	0.042	-0.024	0.013	0.057	-0.041
	(0.023)	(0.025)	(0.030)	(0.033)	(0.043)	(0.048)
Q3 proximity	0.0053	0.043	-0.055	0.0054	0.064	0.0048
	(0.0225)	(0.024)	(0.031)	(0.0342)	(0.043)	(0.0417)
Q4 proximity	0.023	0.037	-0.035	-0.030	0.059	-0.017
	(0.022)	(0.023)	(0.031)	(0.031)	(0.043)	(0.035)
Age	-0.00123**	-0.00126**	0.00059	0.00056	0.00029	0.00026
	(0.00042)	(0.00042)	(0.00061)	(0.00065)	(0.00076)	(0.00076)
Female	-0.063***	-0.062***	-0.060***	-0.060***	-0.036	-0.036
	(0.013)	(0.013)	(0.018)	(0.018)	(0.022)	(0.022)
Black	0.023	0.022	0.042	0.041	-0.044	-0.045
	(0.022)	(0.022)	(0.028)	(0.028)	(0.037)	(0.038)
Asian	-0.043	-0.041	0.027	0.026	-0.034	-0.039
	(0.032)	(0.032)	(0.050)	(0.050)	(0.058)	(0.058)
Other race	-0.023	-0.026	-0.036	-0.035	0.065	0.064
	(0.024)	(0.024)	(0.035)	(0.035)	(0.052)	(0.053)
Hispanic/Latino	0.025	0.028	0.0077	0.0077	-0.090*	-0.085*
	(0.020)	(0.020)	(0.0249)	(0.0248)	(0.036)	(0.036)
College	0.072***	0.070***	0.050*	0.049*	0.0016	0.0011
	(0.013)	(0.014)	(0.023)	(0.023)	(0.0247)	(0.0251)
Employed	0.066***	0.067***	0.035	0.034	0.050	0.049
	(0.014)	(0.014)	(0.021)	(0.021)	(0.029)	(0.029)
Income Q2	0.017	0.016	-0.014	-0.012	0.031	0.028
	(0.016)	(0.016)	(0.028)	(0.028)	(0.035)	(0.035)
Income Q3	0.007	0.0067	-0.045	-0.044	0.013	0.014
	(0.018)	(0.0175)	(0.030)	(0.030)	(0.036)	(0.036)
Income Q4	0.056*	0.058**	-0.021	-0.020	0.048	0.048
	(0.022)	(0.022)	(0.036)	(0.036)	(0.037)	(0.037)
Income Q5	0.074**	0.076**	-0.034	-0.031	0.077	0.075
	(0.026)	(0.026)	(0.041)	(0.041)	(0.049)	(0.049)
Republican	-0.009	-0.0093	-0.169***	-0.170***	-0.15***	-0.15***
	(0.015)	(0.0151)	(0.022)	(0.022)	(0.03)	(0.03)
Neither party	-0.069***	-0.070***	-0.221***	-0.223***	-0.105**	-0.105**
	(0.017)	(0.017)	(0.025)	(0.025)	(0.038)	(0.038)
Global warming index	0.128***	0.128***	0.074*	0.074*	0.566***	0.566***
	(0.021)	(0.021)	(0.035)	(0.035)	(0.044)	(0.044)
Population density	-0.0048	-0.0097	0.0150	0.0151	0.013	0.019
	(0.0072)	(0.0076)	(0.0092)	(0.0092)	(0.015)	(0.015)
County college share $(t-1)$	0.0045	0.004	-0.00032	-0.0015	0.015	0.018
	(0.0139)	(0.014)	(0.01979)	(0.0202)	(0.025)	(0.025)
County poverty share $(t-1)$	0.0085	0.0097	-0.0041	-0.0031	-0.0072	-0.0035
	(0.0104)	(0.0102)	(0.0145)	(0.0145)	(0.0185)	(0.0184)
County foreign-born share $(t-1)$	-0.0004	-0.0034	-0.00038	-0.0012	-0.012	-0.013
	(0.0093)	(0.0093)	(0.01211)	(0.0123)	(0.019)	(0.019)
Median county housing costs $(t-1)$	-0.038**	-0.041**	0.006	0.0069	0.00041	0.0075
	(0.014)	(0.014)	(0.018)	(0.0181)	(0.02192)	(0.0214)
Faster broadband access $(t-1)$	0.013	0.017	-0.043	-0.044	0.053	0.054
	(0.021)	(0.021)	(0.028)	(0.028)	(0.030)	(0.030)
County GDP (log) $(t-1)$	0.059	0.051	0.049	0.051	0.040	0.039
	(0.039)	(0.039)	(0.050)	(0.050)	(0.063)	(0.063)
Labor force (log) $(t-1)$	-0.071	-0.063	-0.045	-0.048	-0.051	-0.052
	(0.038)	(0.038)	(0.048)	(0.048)	(0.059)	(0.059)
County unemployment rate $(t-1)$	-0.0086	-0.0061	0.0243*	0.0259**	0.022	0.025*
	(0.0079)	(0.0078)	(0.0095)	(0.0099)	(0.012)	(0.012)
Highway access	0.015	0.016	0.076*	0.074*	0.0021	0.011
	(0.020)	(0.020)	(0.033)	(0.032)	(0.0412)	(0.041)
County income pc $(t-1)$	0.024	0.028	-0.0027	-0.0012	-0.0099	-0.012
	(0.015)	(0.016)	(0.0159)	(0.0159)	(0.0234)	(0.023)
N	5026	5026	3034	3034	1487	1487
Adjusted R^2	0.075	0.074	0.069	0.068	0.182	0.180
Covariates	Yes	Yes	Yes	Yes	Yes	Yes
Sample Fixed Effects	Yes	Yes	Yes	Yes	Yes	Yes
State Fixed Effects	Yes	Yes	Yes	Yes	Yes	Yes

Notes: Each column reports a separate linear probability model. Unit of analysis is the individual survey respondent. Models 1–2: outcome = 1 if the respondent reports a local green project, 0 otherwise. Models 3–4: outcome = 1 if the respondent credits the Biden Administration for local green investments. Models 5–6: outcome = 1 if the respondent perceives a benefit from local green projects. Estimates are OLS with Conley standard errors (50 km threshold). Continuous covariates are standardized. * $p < 0.05$, ** $p < 0.01$, *** $p < 0.001$.

S3.4 Power Analyses

The main text analysis relies on a categorical measure of distance quintiles from new clean energy projects. To assess statistical power, we focus on the contrast between individuals in the nearest quintile and those in the farthest quintile. This contrast is the most likely case for an effect of proximity on credit attribution or recognition, so any other comparisons necessarily have less statistical power for the same minimally detectable effect.

Three binary outcomes were considered: (i) whether respondents indicated that President Biden was "extremely" or "very" responsible for new clean energy investments in their state, (ii) whether respondents recognized the presence of a new clean energy project in their community, and (iii) whether respondents thought green investments were economically beneficial.

Power was calculated analytically using the following procedure. First, for each proximity measure, the outcome mean was estimated from the control group (respondents in the farthest quintile). A minimum detectable effect (MDE) was then specified. Next, 1,000 datasets were simulated, each with the same number of respondent–state observations as in the observed data. In each simulation, the outcome variable was drawn from a binomial distribution with the probability parameter determined by the control group mean and the assumed MDE. The treatment effect was modeled as decaying with distance for the intermediate quintiles of the categorical proximity measure.

For each assumed MDE, the simulated outcome was regressed on the treatment indicator, including the same state fixed effects and covariates as in the main specification. The proportion of estimates that were correctly signed and statistically significant at the 5% level was recorded as the analytical power.

Figures S2–S4 present power analyses for the three main outcomes. The horizontal blue line indicates the MDE for which the design has 80% power ($\alpha = 0.05$). Since each outcome ranges from 0-1, multiply the MDE by 100 for interpretation in percentage point shifts.

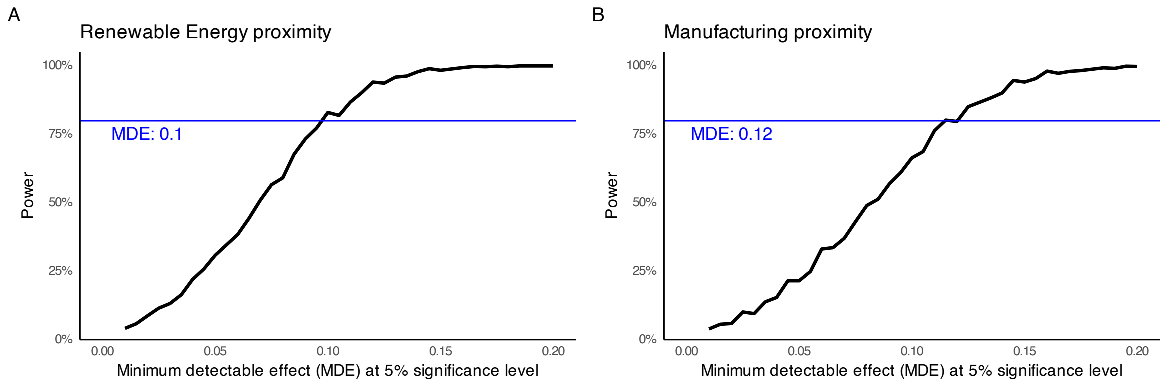

Fig. S2: Analytical power analysis, credit attribution outcome.

It is reasonable to imagine that reformers behind the IRA had anticipated that people living near projects would be at least 10 percentage points more likely to notice them and, in turn, credit the Biden Administration. While partisan polarization may constrain belief change among Democrats and Republicans, a substantial share of the public identifies as independent, so at least some of these respondents could update their views if benefiting from investments. We therefore treat shifts of this magnitude as substantively meaningful benchmarks. Although our design cannot reliably detect smaller effects, such effects would be more difficult to interpret as politically consequential if

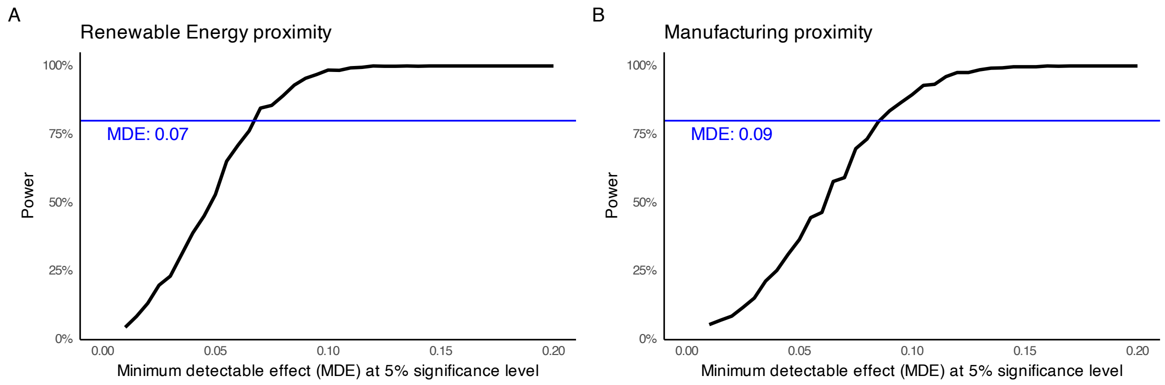

Fig. S3: Analytical power analysis, recognition outcome.

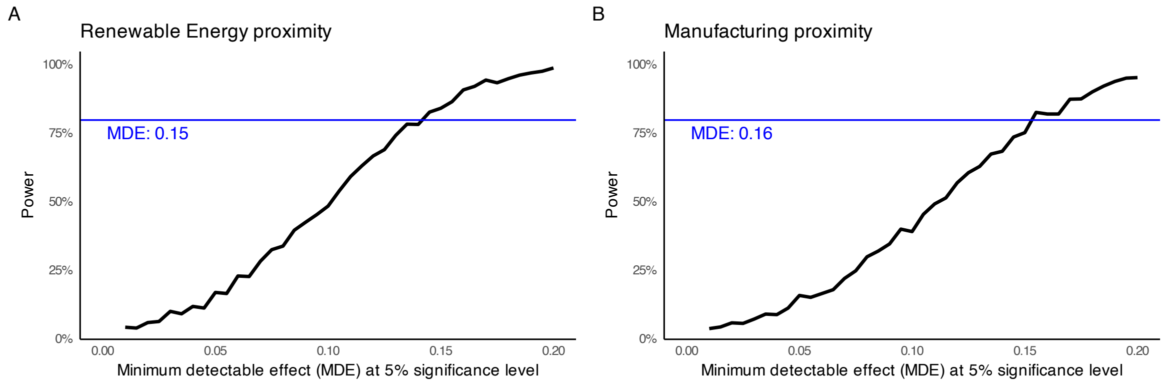

Fig. S4: Analytical power analysis, benefit outcome.

they existed.

There are limits to the analytic power calculation. First, the procedure assumes independent binomial draws and a decay of treatment effects across distance bins, which may not fully reflect real-world correlation structures or alternative functional forms. Second, because simulated outcomes are generated without reference to covariates, the role of covariate adjustment is limited to variance reduction, which could result in under-estimating power.

S3.5 Robustness Checks

S3.5.1 Sensitivity to Omitted Variable Bias

Tables S7 and S8 report sensitivity analysis diagnostics following Cinelli and Hazlett (2020). For the first proximity quintile, the robustness value $RV_{q=1}$ is 3.0% for manufacturing and 4.2% for renewables. In other words, an unobserved confounder would need to explain at least 3.0% and 4.2% of the residual variance of both the treatment and the outcome to fully eliminate the estimated effects.

The robustness values for statistical significance, $RV_{q=1,\alpha=0.05}$, are 0.3% for manufacturing and 1.5% for renewables. These values indicate the strength an unobserved confounder would need to reduce the lower bound of the 95% confidence interval to zero.

Finally, $R^2_{Y\sim D|\mathbf{X}}$ shows that even if we assume an extreme scenario where unobserved confounders explain all of the remaining variance in the outcome, such a confounder would need to account for at least 0.1% (manufacturing) or 0.2% (renewables) of the residual variance in the treatment to eliminate the observed effect.

Interpreting these values requires domain knowledge. Any hypothetical unobserved confounder would need to be more than the strongest observed benchmark covariates—labor force participation for manufacturing and household income for renewables—to change our conclusions.

Table S7: Sensitivity analysis for visibility outcome, renewable energy proximity (q1) model

Outcome: *Visibility (=1)*						
Treatment:	Est.	S.E.	t-value	$R^2_{Y\sim D\|\mathbf{X}}$	$RV_{q=1}$	$RV_{q=1,\alpha=0.05}$
Renewable energy proximity (Q1)	0.066	0.022	3.024	0.200%	4.200%	1.500%
df = 4942	*Bound (1x Income Q4)*: $R^2_{Y\sim Z\|\mathbf{X},D} = 0.100\%$, $R^2_{D\sim Z\|\mathbf{X}} = 0.400\%$					

Table S8: Sensitivity analysis for visibility outcome, manufacturing proximity (q1) model

Outcome: *Visibility (=1)*						
Treatment:	Est.	S.E.	t-value	$R^2_{Y\sim D\|\mathbf{X}}$	$RV_{q=1}$	$RV_{q=1,\alpha=0.05}$
Manufacturing proximity (Q1)	0.063	0.029	2.170	0.100%	3.000%	0.300%
df = 4942	*Bound (1x Labor force (log) $(t-1)$)*: $R^2_{Y\sim Z\|\mathbf{X},D} = 0.300\%$, $R^2_{D\sim Z\|\mathbf{X}} = 0.000\%$					

S3.5.2 Alternative Standard Errors

Table S9: Robustness to 100 km Conley standard error cutoff: Linear probability models of project visibility and credit attribution.

	Visibility (=1)		Credit Biden (=1)		Benefits (=1)	
	Renewables	Manufacturing	Renewables	Manufacturing	Renewables	Manufacturing
Q1 proximity	0.066**	0.063*	-0.013	0.005	0.0026	-0.013
	(0.021)	(0.029)	(0.032)	(0.037)	(0.0462)	(0.044)
Q2 proximity	0.045	0.042	-0.024	0.013	0.057	-0.041
	(0.024)	(0.024)	(0.032)	(0.035)	(0.042)	(0.042)
Q3 proximity	0.0053	0.043	-0.055	0.0054	0.064	0.0048
	(0.0227)	(0.022)	(0.032)	(0.0329)	(0.042)	(0.0388)
Q4 proximity	0.023	0.037	-0.035	-0.03	0.059	-0.017
	(0.021)	(0.021)	(0.035)	(0.03)	(0.043)	(0.035)
N	5026	5026	3034	3034	1487	1487
Adjusted R^2	0.075	0.074	0.069	0.068	0.182	0.180
Covariates	Yes	Yes	Yes	Yes	Yes	Yes
Sample Fixed Effects	Yes	Yes	Yes	Yes	Yes	Yes
State Fixed Effects	Yes	Yes	Yes	Yes	Yes	Yes

Notes: Each column reports a separate linear probability model. Unit of analysis is the individual survey respondent. Models 1–2: outcome = 1 if the respondent reports a local green project, 0 otherwise. Models 3–4: outcome = 1 if the respondent credits the Biden Administration for local green investments. Models 5–6: outcome = 1 if the respondent perceives a benefit from local green projects. Estimates are OLS with Conley standard errors (100 km threshold). Continuous covariates are standardized. * $p < 0.05$, ** $p < 0.01$, *** $p < 0.001$.

Table S10: Robustness to 200 km Conley standard error cutoff: Linear probability models of project visibility and credit attribution.

	Visibility (=1)		Credit Biden (=1)		Benefits (=1)	
	Renewables	Manufacturing	Renewables	Manufacturing	Renewables	Manufacturing
Q1 proximity	0.066**	0.063*	-0.013	0.005	0.0026	-0.013
	(0.022)	(0.028)	(0.035)	(0.030)	(0.0404)	(0.043)
Q2 proximity	0.045	0.042	-0.024	0.013	0.057	-0.041
	(0.025)	(0.024)	(0.038)	(0.030)	(0.035)	(0.037)
Q3 proximity	0.0053	0.043	-0.055	0.0054	0.064	0.0048
	(0.0238)	(0.023)	(0.036)	(0.0280)	(0.041)	(0.0351)
Q4 proximity	0.023	0.037	-0.035	-0.030	0.059	-0.017
	(0.021)	(0.019)	(0.040)	(0.026)	(0.036)	(0.038)
N	5026	5026	3034	3034	1487	1487
Adjusted R^2	0.075	0.074	0.069	0.068	0.182	0.180
Covariates	Yes	Yes	Yes	Yes	Yes	Yes
Sample Fixed Effects	Yes	Yes	Yes	Yes	Yes	Yes
State Fixed Effects	Yes	Yes	Yes	Yes	Yes	Yes

Notes: Each column reports a separate linear probability model. Unit of analysis is the individual survey respondent. Models 1–2: outcome = 1 if the respondent reports a local green project, 0 otherwise. Models 3–4: outcome = 1 if the respondent credits the Biden Administration for local green investments. Models 5–6: outcome = 1 if the respondent perceives a benefit from local green projects. Estimates are OLS with Conley standard errors (200 km threshold). Continuous covariates are standardized. * $p < 0.05$, ** $p < 0.01$, *** $p < 0.001$.

Table S11: Robustness to state-clustered standard errors: Linear probability models of project visibility and credit attribution.

	Visibility (=1)		Credit Biden (=1)		Benefits (=1)	
	Renewables	Manufacturing	Renewables	Manufacturing	Renewables	Manufacturing
Q1 proximity	0.066**	0.063*	-0.013	0.005	0.0026	-0.013
	(0.020)	(0.030)	(0.026)	(0.038)	(0.0382)	(0.037)
Q2 proximity	0.045	0.042	-0.024	0.013	0.057	-0.041
	(0.026)	(0.024)	(0.036)	(0.031)	(0.030)	(0.036)
Q3 proximity	0.0053	0.043	-0.055	0.0054	0.064	0.0048
	(0.0205)	(0.023)	(0.030)	(0.0361)	(0.037)	(0.0404)
Q4 proximity	0.023	0.037	-0.035	-0.030	0.059	-0.017
	(0.018)	(0.020)	(0.032)	(0.028)	(0.034)	(0.048)
N	5026	5026	3034	3034	1487	1487
Adjusted R^2	0.075	0.074	0.069	0.068	0.182	0.180
Covariates	Yes	Yes	Yes	Yes	Yes	Yes
Sample Fixed Effects	Yes	Yes	Yes	Yes	Yes	Yes
State Fixed Effects	Yes	Yes	Yes	Yes	Yes	Yes

Notes: Each column reports a separate linear probability model. Unit of analysis is the individual survey respondent. Models 1–2: outcome = 1 if the respondent reports a local green project, 0 otherwise. Models 3–4: outcome = 1 if the respondent credits the Biden Administration for local green investments. Models 5–6: outcome = 1 if the respondent perceives a benefit from local green projects. Estimates are OLS with cluster-robust standard errors by state. Continuous covariates are standardized. * $p < 0.05$, ** $p < 0.01$, *** $p < 0.001$.

S3.5.3 Alternative Geocoordinates

Table S12: Robustness to alternative geocoordinates: Linear probability models of project visibility and credit attribution.

	Visibility (=1)		Credit Biden (=1)		Benefits (=1)	
	Renewables	Manufacturing	Renewables	Manufacturing	Renewables	Manufacturing
Q1 proximity	0.056*	0.060*	-0.012	-0.00097	0.00016	-0.018
	(0.022)	(0.029)	(0.032)	(0.03866)	(0.04503)	(0.046)
Q2 proximity	0.040	0.039	-0.036	0.016	0.054	-0.042
	(0.023)	(0.026)	(0.031)	(0.035)	(0.043)	(0.048)
Q3 proximity	-0.002	0.044	-0.060	-0.0028	0.058	0.0059
	(0.023)	(0.024)	(0.032)	(0.0359)	(0.044)	(0.0424)
Q4 proximity	0.018	0.038	-0.041	-0.029	0.064	-0.0074
	(0.022)	(0.023)	(0.031)	(0.032)	(0.042)	(0.0346)
N	4856	4856	2931	2931	1451	1451
Adjusted R^2	0.065	0.064	0.062	0.061	0.176	0.174
Covariates	Yes	Yes	Yes	Yes	Yes	Yes
Sample Fixed Effects	Yes	Yes	Yes	Yes	Yes	Yes
State Fixed Effects	Yes	Yes	Yes	Yes	Yes	Yes

Notes: This analysis is performed on the subset of respondents whose IP addresses and ZIP codes imply similar longitude-latitude geo-coordinates. Each column reports a separate linear probability model. Unit of analysis is the individual survey respondent. Models 1–2: outcome = 1 if the respondent reports a local green project, 0 otherwise. Models 3–4: outcome = 1 if the respondent credits the Biden Administration for local green investments. Models 5–6: outcome = 1 if the respondent perceives a benefit from local green projects. Estimates are OLS with Conley standard errors (50 km threshold). Continuous covariates are standardized. * $p < 0.05$, ** $p < 0.01$, *** $p < 0.001$.

Table S13: Robustness to spherical distance metric: Linear probability models of project visibility and credit attribution.

	Visibility (=1)		Credit Biden (=1)		Benefits (=1)	
	Renewables	Manufacturing	Renewables	Manufacturing	Renewables	Manufacturing
Q1 proximity	0.066**	0.063*	-0.013	0.005	0.0026	-0.013
	(0.022)	(0.029)	(0.031)	(0.038)	(0.0442)	(0.046)
Q2 proximity	0.045	0.042	-0.024	0.013	0.057	-0.041
	(0.023)	(0.025)	(0.030)	(0.033)	(0.043)	(0.047)
Q3 proximity	0.0053	0.043	-0.055	0.0054	0.064	0.0048
	(0.0224)	(0.024)	(0.031)	(0.0345)	(0.043)	(0.0413)
Q4 proximity	0.023	0.037	-0.035	-0.030	0.059	-0.017
	(0.022)	(0.023)	(0.031)	(0.031)	(0.043)	(0.034)
N	5026	5026	3034	3034	1487	1487
Adjusted R^2	0.075	0.074	0.069	0.068	0.182	0.180
Covariates	Yes	Yes	Yes	Yes	Yes	Yes
Sample Fixed Effects	Yes	Yes	Yes	Yes	Yes	Yes
State Fixed Effects	Yes	Yes	Yes	Yes	Yes	Yes

Notes: Each column reports a separate linear probability model. Unit of analysis is the individual survey respondent. Models 1–2: outcome = 1 if the respondent reports a local green project, 0 otherwise. Models 3–4: outcome = 1 if the respondent credits the Biden Administration for local green investments. Models 5–6: outcome = 1 if the respondent perceives a benefit from local green projects. Estimates are OLS with Conley standard errors (50 km threshold, spherical distance). Continuous covariates are standardized. * $p < 0.05$, ** $p < 0.01$, *** $p < 0.001$.

S3.5.4 Continuous Distance Measure

Table S14: Robustness to continuous distance specification: Linear probability models of project visibility, credit attribution, and perceived benefits.

	Visibility (=1)		Credit Biden (=1)		Benefits (=1)	
	Renewables	Manufacturing	Renewables	Manufacturing	Renewables	Manufacturing
Distance to renewables (log)	-0.0309***		0.0013		0.0034	
	(0.0081)		(0.0120)		(0.0170)	
Distance to manufacturing (log)		-0.0171*		0.00017		-0.0064
		(0.0074)		(0.01105)		(0.0151)
N	5026	5026	3034	3034	1487	1487
Adjusted R^2	0.076	0.074	0.068	0.068	0.181	0.181
Covariates	Yes	Yes	Yes	Yes	Yes	Yes
Sample Fixed Effects	Yes	Yes	Yes	Yes	Yes	Yes
State Fixed Effects	Yes	Yes	Yes	Yes	Yes	Yes

Notes: Each column reports a separate linear probability model. Unit of analysis is the individual survey respondent. Models 1–2: outcome = 1 if the respondent reports a local green project, 0 otherwise. Models 3–4: outcome = 1 if the respondent credits the Biden Administration for local green investments. Models 5–6: outcome = 1 if the respondent perceives a benefit from local green projects. Distance is log-transformed (continuous). Estimates are OLS with Conley standard errors (50 km threshold). Continuous covariates are standardized. * $p < 0.05$, ** $p < 0.01$, *** $p < 0.001$.

S3.5.5 Weighted Regressions

Table S15: Robustness to survey weights: Linear probability models of project visibility, credit attribution, and perceived benefits.

	Visibility (=1)		Credit Biden (=1)		Benefits (=1)	
	Renewables	Manufacturing	Renewables	Manufacturing	Renewables	Manufacturing
Q1 proximity	0.064**	0.082**	-0.034	0.00067	0.0026	-0.013
	(0.024)	(0.031)	(0.035)	(0.04199)	(0.0441)	(0.046)
Q2 proximity	0.039	0.061*	-0.0084	0.0023	0.057	-0.041
	(0.026)	(0.028)	(0.0341)	(0.0372)	(0.043)	(0.048)
Q3 proximity	-0.0017	0.054*	-0.065	-0.0042	0.064	0.0048
	(0.0251)	(0.026)	(0.035)	(0.0352)	(0.043)	(0.0417)
Q4 proximity	0.022	0.068**	-0.029	-0.058	0.059	-0.017
	(0.023)	(0.026)	(0.033)	(0.033)	(0.043)	(0.035)
N	5026	5026	3034	3034	1487	1487
Adjusted R^2	0.074	0.073	0.075	0.074	0.182	0.180
Covariates	Yes	Yes	Yes	Yes	Yes	Yes
Sample Fixed Effects	Yes	Yes	Yes	Yes	Yes	Yes
State Fixed Effects	Yes	Yes	Yes	Yes	Yes	Yes

Notes: Each column reports a separate linear probability model. Unit of analysis is the individual survey respondent. Models 1–2: outcome = 1 if the respondent reports a local green project, 0 otherwise. Models 3–4: outcome = 1 if the respondent credits the Biden Administration for local green investments. Models 5–6: outcome = 1 if the respondent perceives a benefit from local green projects. Estimates are weighted OLS (ACS-raked weights) with Conley standard errors (50 km threshold). Continuous covariates are standardized. * $p < 0.05$, ** $p < 0.01$, *** $p < 0.001$.

S3.5.6 Media Market Fixed Effects

To account for spatial spillovers, such as from shared local news markets, a robustness check re-estimates the models including fixed effects for Nielsen Designated Market Areas (DMAs). These fixed effects hold constant any unobserved shocks or common information environments at the media-market level.

Table S16: Robustness to DMA fixed effects: Linear probability models of project visibility and credit attribution.

	Visibility (=1)		Credit Biden (=1)		Benefits (=1)	
	Renewables	Manufacturing	Renewables	Manufacturing	Renewables	Manufacturing
Q1 proximity	0.088***	0.018	-0.045	0.0057	-0.035	-0.13
	(0.025)	(0.046)	(0.035)	(0.0508)	(0.057)	(0.10)
Q2 proximity	0.057*	0.014	-0.043	0.063	0.017	-0.125
	(0.026)	(0.041)	(0.035)	(0.050)	(0.056)	(0.096)
Q3 proximity	0.021	0.055	-0.076*	0.091*	0.019	-0.013
	(0.025)	(0.037)	(0.036)	(0.042)	(0.056)	(0.068)
Q4 proximity	0.043	0.035	-0.045	0.0058	0.038	-0.014
	(0.024)	(0.027)	(0.036)	(0.0352)	(0.053)	(0.043)
N	5012	5012	3017	3017	1455	1455
Adjusted R^2	0.074	0.072	0.071	0.072	0.178	0.178
Covariates	Yes	Yes	Yes	Yes	Yes	Yes
Sample Fixed Effects	Yes	Yes	Yes	Yes	Yes	Yes
DMA Fixed Effects	Yes	Yes	Yes	Yes	Yes	Yes

Notes: Each column reports a separate linear probability model. Unit of analysis is the individual survey respondent. Models 1–2: outcome = 1 if the respondent reports a local green project, 0 otherwise. Models 3–4: outcome = 1 if the respondent credits the Biden Administration for local green investments. Models 5–6: outcome = 1 if the respondent perceives a benefit from local green projects. Estimates are OLS with Conley standard errors (50 km threshold). Continuous covariates are standardized. * $p < 0.05$, ** $p < 0.01$, *** $p < 0.001$.

S3.5.7 Additional Credit Recipients

Table S17: Robustness to different credit attribution outcomes: Linear probability models of credit attribution.

	Governor (=1)		State lawmakers (=1)		Congress (=1)		Local officials (=1)		Markets (=1)	
	Renewables	Manufacturing	Renewables	Manufacturing	Renewables	Manufacturing	Renewables	Manufacturing	Renewables	Manufacturing
Q1 proximity	0.011	-0.032	-0.020	0.018	-0.018	0.048	-0.050	0.056	-0.017	-0.0056
	(0.034)	(0.044)	(0.033)	(0.044)	(0.029)	(0.036)	(0.033)	(0.037)	(0.033)	(0.0367)
Q2 proximity	-0.023	-0.046	0.0011	0.035	-0.017	0.036	-0.0065	0.014	-0.023	-0.042
	(0.034)	(0.036)	(0.0311)	(0.037)	(0.030)	(0.032)	(0.0306)	(0.033)	(0.032)	(0.033)
Q3 proximity	-0.023	-0.029	-0.052	0.036	-0.045	0.015	-0.052	0.051	-0.039	0.025
	(0.033)	(0.039)	(0.032)	(0.037)	(0.029)	(0.032)	(0.031)	(0.034)	(0.029)	(0.035)
Q4 proximity	-0.053	-0.030	-0.048	0.014	-0.011	8e-04	-0.074*	0.011	-0.05	-0.038
	(0.035)	(0.031)	(0.030)	(0.033)	(0.027)	(3e-02)	(0.031)	(0.032)	(0.03)	(0.032)
N	3034	3034	3034	3034	3034	3034	3034	3034	3034	3034
Adjusted R^2	0.042	0.041	0.047	0.045	0.079	0.079	0.048	0.046	0.055	0.056
Covariates	Yes	Yes	Yes	Yes	Yes	Yes	Yes	Yes	Yes	Yes
Sample Fixed Effects	Yes	Yes	Yes	Yes	Yes	Yes	Yes	Yes	Yes	Yes
State Fixed Effects	Yes	Yes	Yes	Yes	Yes	Yes	Yes	Yes	Yes	Yes

Notes: Each column reports a separate linear probability model. Unit of analysis is the individual survey respondent. Outcome = 1 if the respondent credits the column header actor for local green investments. Estimates are OLS with Conley standard errors (50 km threshold). Continuous covariates are standardized. * $p < 0.05$, ** $p < 0.01$, *** $p < 0.001$.

S3.5.8 One-Year Temporal Window

Table S18: Robustness to 1-year temporal window: Linear probability models of project visibility, credit attribution, and perceived benefits

	Visibility (=1)		Credit Biden (=1)		Benefits (=1)	
	Renewables	Manufacturing	Renewables	Manufacturing	Renewables	Manufacturing
Q1 proximity	0.066**	0.017	-0.013	0.021	0.0026	-0.042
	(0.022)	(0.027)	(0.031)	(0.035)	(0.0441)	(0.062)
Q2 proximity	0.045	0.011	-0.024	-0.023	0.057	-0.074
	(0.023)	(0.028)	(0.030)	(0.034)	(0.043)	(0.062)
Q3 proximity	0.0053	-0.0043	-0.055	-0.048	0.064	-0.023
	(0.0225)	(0.0293)	(0.031)	(0.035)	(0.043)	(0.062)
Q4 proximity	0.023	-0.007	-0.035	-0.0079	0.059	-0.020
	(0.022)	(0.025)	(0.031)	(0.0321)	(0.043)	(0.059)
N	5026	5026	3034	3034	1487	1487
Adjusted R^2	0.075	0.073	0.069	0.069	0.182	0.180
Covariates	Yes	Yes	Yes	Yes	Yes	Yes
Sample Fixed Effects	Yes	Yes	Yes	Yes	Yes	Yes
State Fixed Effects	Yes	Yes	Yes	Yes	Yes	Yes

Notes: This analysis uses projects that opened within 1 year of survey completion instead of the main 2-year window. Each column reports a separate linear probability model. Unit of analysis is the individual survey respondent. Models 1–2: outcome = 1 if the respondent reports a local green project, 0 otherwise. Models 3–4: outcome = 1 if the respondent credits the Biden Administration for local green investments. Models 5–6: outcome = 1 if the respondent perceives a benefit from local green projects. Estimates are OLS with Conley standard errors (50 km threshold). Continuous covariates are standardized. * $p < 0.05$, ** $p < 0.01$, *** $p < 0.001$.

S3.6 Treatment Effect Heterogeneity

We assess whether the effect of proximity varies with a moderator M_i. The specification extends the equation in Materials and Methods by interacting M_i with proximity quintile indicators:

$$Y_i = M_i\lambda + M_i \times \sum_{q=1}^{4} \mathbb{1}[Distance_i \in Q_q]\beta_q + X_i^{\top}\gamma + State_i + Sample_i + \epsilon_i, \tag{S1}$$

where Q_5 (farthest quintile) is the omitted category. Models are estimated using OLS on the full sample for which the outcome is observed. Standard errors are clustered by state since diagnostic tests found these to be more reliable than the Conley estimator given the more saturated model specification. No weights are applied. All subsequent subsections apply this specification to a given moderator.

S3.6.1 Manufacturing Operational Status

We assess whether the effect of proximity varies by project status. For manufacturing projects, the moderator distinguishes between "operating" (projects categorized by Big Green Machine as "Operating" or Operating Partially; Under Construction") and "other" (projects categorized as "Paused," "Pilot," "Planned," "Sold," or "Under Construction"). The analysis in the main text examines the subset of projects in the former category, operating or operating partially/under construction; the heterogeneity analysis adds additional projects beyond that subset.

Fig. S5 reports the average marginal effect of proximity (relative to Q5) for each status category. The visibility effect at Q1 appears only for projects that are fully or partially operational. The interaction term is negative but imprecisely estimated, so we cannot conclude that the visibility effect differs by project status (Table S19).

There is no moderating effect of manufacturing project status on proximity for the credit attribution and perceived benefit outcomes.

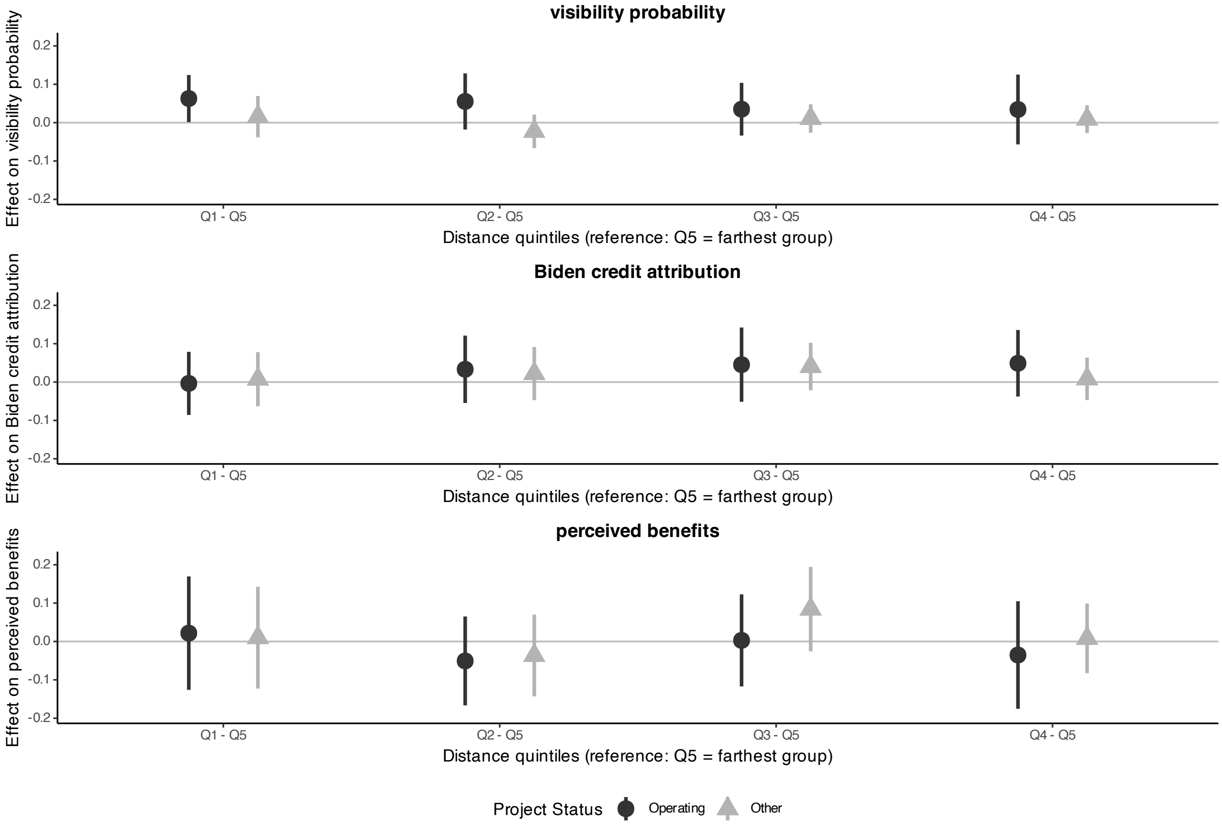

Fig. S5: Heterogeneous effects of clean energy manufacturing proximity on recognition by project status. Plot depicts the average marginal effect of a proximity quintile (relative to Q5) across moderator values. Bars denote 95% confidence intervals with cluster-robust standard errors by state. Equation S1 describes the model specification. Table S19 reports interaction terms and sample sizes.

Table S19: Heterogeneous proximity effects by manufacturing project status

	Manufacturing		
	Visibility	Credit	Benefit
Q1 proximity	0.063	-0.0035	0.022
	(0.031)	(0.0419)	(0.075)
Q2 proximity	0.055	0.033	-0.051
	(0.037)	(0.045)	(0.059)
Q3 proximity	0.035	0.045	0.0029
	(0.035)	(0.049)	(0.0612)
Q4 proximity	0.034	0.049	-0.035
	(0.046)	(0.044)	(0.072)
Other	0.017	-0.0028	-0.053
	(0.030)	(0.0374)	(0.053)
Q1 × Other	-0.047	0.011	-0.012
	(0.046)	(0.050)	(0.090)
Q2 × Other	-0.078	-0.011	0.014
	(0.043)	(0.052)	(0.067)
Q3 × Other	-0.024	-0.0048	0.081
	(0.045)	(0.0670)	(0.089)
Q4 × Other	-0.025	-0.041	0.043
	(0.051)	(0.050)	(0.071)
N	5026	3034	1487
Adjusted R^2	0.073	0.067	0.182
Covariates	Yes	Yes	Yes
State Fixed Effects	Yes	Yes	Yes

Notes: Each column reports a separate linear probability model with interactions between proximity and manufacturing project status. Unit of analysis is the individual survey respondent. Visibility = 1 if respondent reports a local green project, 0 otherwise. Credit = 1 if respondent credits the Biden Administration for local green investments. Benefit = 1 if respondent perceives a benefit from local green projects. Estimates are OLS with cluster-robust standard errors by state in parentheses. $^{*}p < 0.05$, $^{**}p < 0.01$, $^{***}p < 0.001$.

S3.6.2 Renewable Energy Operational Status

For renewable energy projects, the status moderator has two levels: "construction" and "pre-construction." Construction refers to plants that are not yet fully operational but have active building underway. Pre-construction refers to projects that are planned or have received approvals but where building has not yet begun, although site preparation may be underway.

Figure S6 shows the average marginal effects (AME) of proximity across these categories. Patterns vary by outcome. The only statistically significant ($\alpha = .05$) difference is for the credit outcome, where proximity has a more negative effect for pre-construction projects than under construction projects (Table S20). For the recognition outcome, proximity may have a slightly stronger effect on visibility for pre-construction projects compared to those already under construction.

Although the mechanism behind the negative attribution effect near pre-construction projects is unclear, the result is consistent with our broader finding that the intended beneficiaries of IRA investments are not systematically more likely to credit federal policymakers.

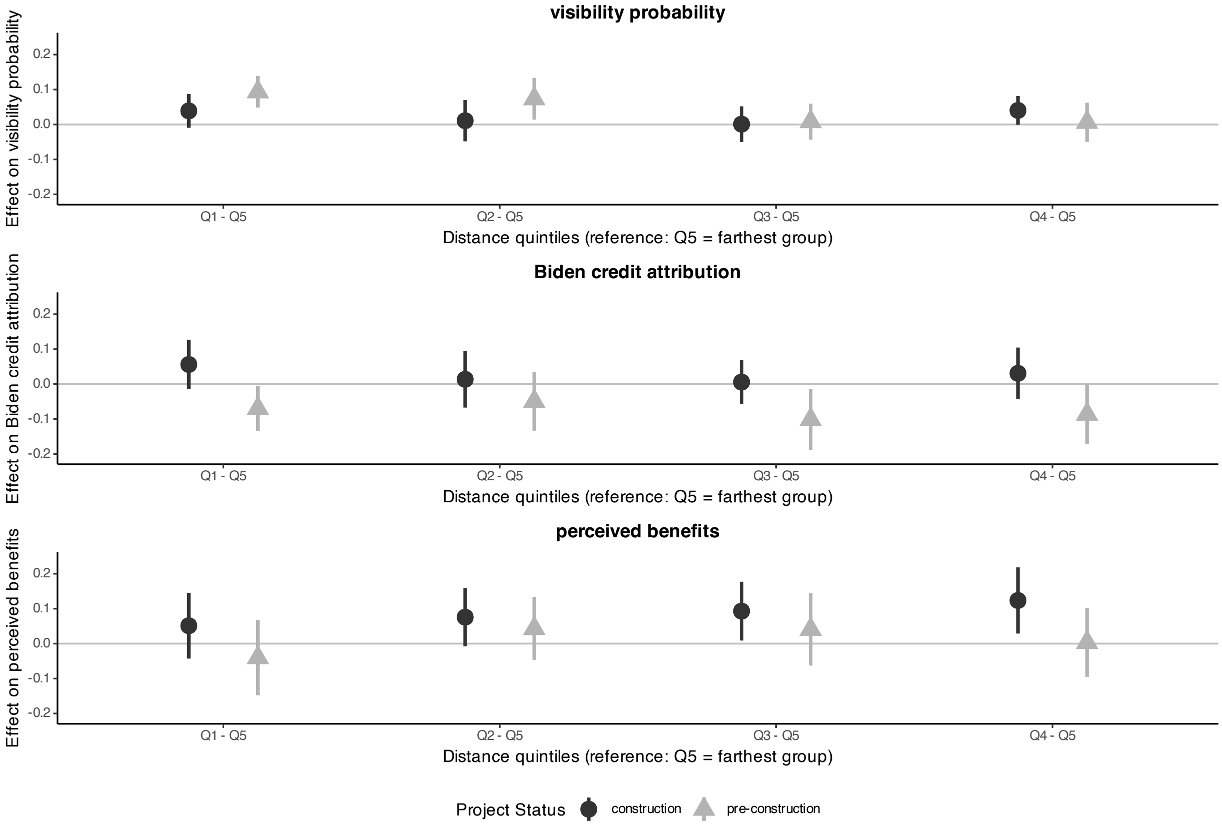

Fig. S6: Heterogeneous effects of renewable generation proximity by project status. Plot depicts the average marginal effect of a proximity quintile (relative to Q5) across moderator values. Bars denote 95% confidence intervals with cluster-robust standard errors by state. Equation S1 describes the model specification. Table S20 reports interaction terms and sample sizes.

Table S20: Heterogeneous proximity effects by renewable energy project status

	Renewable Energy		
	Visibility	Credit	Benefit
Q1 proximity	0.039	0.056	0.051
	(0.025)	(0.036)	(0.048)
Q2 proximity	0.011	0.014	0.076
	(0.030)	(0.041)	(0.043)
Q3 proximity	0.00083	0.0055	0.093*
	(0.02608)	(0.0321)	(0.043)
Q4 proximity	0.040	0.031	0.123*
	(0.021)	(0.038)	(0.048)
pre-construction	-0.012	0.105**	0.052
	(0.024)	(0.033)	(0.050)
Q1 × pre-construction	0.055	-0.126**	-0.091
	(0.029)	(0.046)	(0.072)
Q2 × pre-construction	0.063	-0.063	-0.032
	(0.033)	(0.043)	(0.068)
Q3 × pre-construction	0.0072	-0.107*	-0.052
	(0.0336)	(0.052)	(0.064)
Q4 × pre-construction	-0.034	-0.117*	-0.12
	(0.036)	(0.052)	(0.07)
N	5026	3034	1487
Adjusted R^2	0.076	0.069	0.181
Covariates	Yes	Yes	Yes
State Fixed Effects	Yes	Yes	Yes

Notes: Each column reports a separate linear probability model with interactions between proximity and renewable energy project status. Unit of analysis is the individual survey respondent. Visibility = 1 if respondent reports a local green project, 0 otherwise. Credit = 1 if respondent credits the Biden Administration for local green investments. Benefit = 1 if respondent perceives a benefit from local green projects. Estimates are OLS with cluster-robust standard errors by state in parentheses. $^{*}p < 0.05$, $^{**}p < 0.01$, $^{***}p < 0.001$.

S3.6.3 Renewable Energy Technology

Fig. S7 and Table S21 report the effect of proximity to renewable energy projects conditional on whether the project is wind or solar generation. The range of the y-axis makes it difficult to discern the confidence intervals for the average marginal effect of proximity to solar on the outcomes, but Table S21 shows that proximity to solar increases the probability that respondents report seeing a new green energy project in their communities; there is no statistically distinguishable difference for wind given the null interaction term. People closer to new wind projects (Q2, Q3, and Q4 vs. Q5) may be less likely to credit Biden, and to perceive benefits from the project.

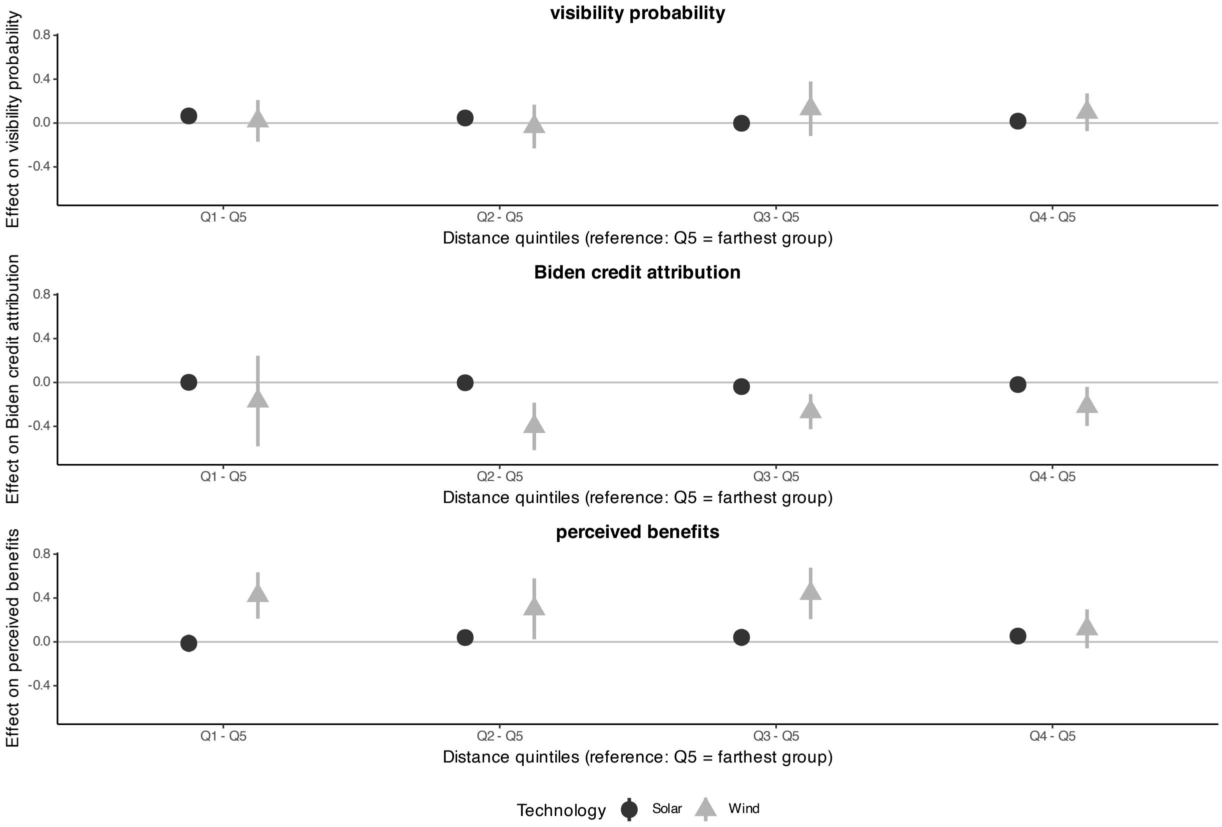

Fig. S7: Heterogeneous effects of renewable generation proximity by technology (solar or wind). Plot depicts the average marginal effect of a proximity quintile (relative to Q5) across moderator values. Bars denote 95% confidence intervals with cluster-robust standard errors by state. Equation S1 describes the model specification. Table S21 reports interaction terms and sample sizes.

Table S21: Heterogeneous proximity effects by renewable energy technology

	Renewable Energy		
	Visibility	Credit	Benefit
Q1 proximity	0.065***	0.0012	-0.013
	(0.019)	(0.0261)	(0.040)
Q2 proximity	0.046	-0.0028	0.039
	(0.026)	(0.0344)	(0.031)
Q3 proximity	-0.002	-0.038	0.041
	(0.019)	(0.031)	(0.039)
Q4 proximity	0.017	-0.019	0.053
	(0.017)	(0.032)	(0.037)
Wind	-0.027	0.172*	-0.187
	(0.063)	(0.066)	(0.098)
Q1 × Wind	-0.045	-0.17	0.44***
	(0.100)	(0.22)	(0.11)
Q2 × Wind	-0.078	-0.40***	0.26
	(0.108)	(0.11)	(0.14)
Q3 × Wind	0.13	-0.229*	0.40**
	(0.13)	(0.093)	(0.13)
Q4 × Wind	0.081	-0.199*	0.066
	(0.090)	(0.091)	(0.100)
N	5026	3034	1487
Adjusted R^2	0.075	0.070	0.184
Covariates	Yes	Yes	Yes
State Fixed Effects	Yes	Yes	Yes

Notes: Each column reports a separate linear probability model with interactions between proximity and renewable energy technology. Unit of analysis is the individual survey respondent. Visibility = 1 if respondent reports a local green project, 0 otherwise. Credit = 1 if respondent credits the Biden Administration for local green investments. Benefit = 1 if respondent perceives a benefit from local green projects. Estimates are OLS with cluster-robust standard errors by state in parentheses. $^{*}p < 0.05$, $^{**}p < 0.01$, $^{***}p < 0.001$.

S3.6.4 Manufacturing Sector

Fig. S8 plots AMEs of proximity to manufacturing projects by sector: batteries, EVs, solar, and wind. For recognition, Q1 proximity is positive for EV and wind, but only the wind estimate is statistically distinguishable from the battery baseline (Table S22).

For credit attribution, proximity effects do not differ consistently across sectors, and none are statistically distinguishable.

For perceived benefits, people nearer to EV facilities (Q1–Q3 vs. Q5) are less likely than those farther away in the same state to view green investments as economically beneficial.

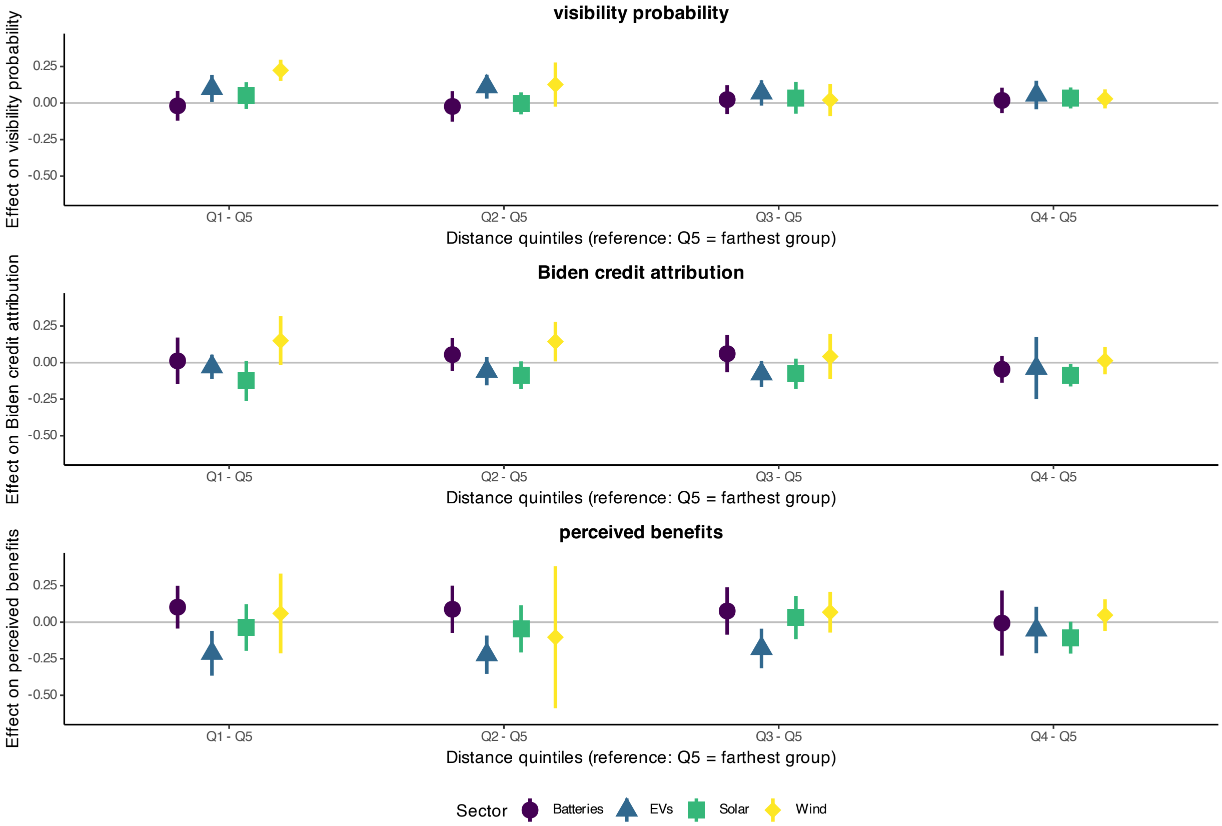

Fig. S8: Heterogeneous effects of clean energy manufacturing proximity by project sector. Plot depicts the average marginal effect of a proximity quintile (relative to Q5) across moderator values. Bars denote 95% confidence intervals with cluster-robust standard errors by state. Equation S1 describes the model specification. Table S22 reports interaction terms and sample sizes.

Table S22: Heterogeneous proximity effects by manufacturing sector

	Manufacturing		
	Visibility	Credit	Benefit
Q1 proximity	-0.019	0.012	0.103
	(0.052)	(0.082)	(0.075)
Q2 proximity	-0.023	0.055	0.088
	(0.053)	(0.058)	(0.082)
Q3 proximity	0.023	0.061	0.077
	(0.051)	(0.065)	(0.083)
Q4 proximity	0.018	-0.046	-0.0062
	(0.044)	(0.047)	(0.1137)
EVs	-0.081	0.047	0.166
	(0.052)	(0.051)	(0.096)
Solar	-0.043	0.056	-0.021
	(0.052)	(0.071)	(0.096)
Wind	-0.037	-0.013	0.015
	(0.057)	(0.065)	(0.071)
Q1 × EVs	0.119	-0.041	-0.315**
	(0.068)	(0.086)	(0.099)
Q1 × Solar	0.070	-0.14	-0.14
	(0.064)	(0.10)	(0.10)
Q1 × Wind	0.243**	0.14	-0.043
	(0.072)	(0.12)	(0.153)
Q2 × EVs	0.135	-0.114	-0.31*
	(0.074)	(0.073)	(0.12)
Q2 × Solar	0.021	-0.142	-0.13
	(0.068)	(0.078)	(0.11)
Q2 × Wind	0.150	0.089	-0.19
	(0.088)	(0.092)	(0.25)
Q3 × EVs	0.046	-0.138	-0.26*
	(0.061)	(0.077)	(0.11)
Q3 × Solar	0.012	-0.14	-0.045
	(0.068)	(0.09)	(0.100)
Q3 × Wind	-0.0031	-0.019	-0.0085
	(0.0898)	(0.101)	(0.1113)
Q4 × EVs	0.037	0.0078	-0.047
	(0.066)	(0.1203)	(0.126)
Q4 × Solar	0.017	-0.041	-0.10
	(0.057)	(0.066)	(0.11)
Q4 × Wind	0.011	0.059	0.055
	(0.062)	(0.063)	(0.125)
N	5026	3034	1487
Adjusted R^2	0.074	0.067	0.182
Covariates	Yes	Yes	Yes
State Fixed Effects	Yes	Yes	Yes

Notes: Each column reports a separate linear probability model with interactions between proximity and manufacturing sector. Unit of analysis is the individual survey respondent. Visibility = 1 if respondent reports a local green project, 0 otherwise. Credit = 1 if respondent credits the Biden Administration for local green investments. Benefit = 1 if respondent perceives a benefit from local green projects. Estimates are OLS with cluster-robust standard errors by state in parentheses. $^{*}p < 0.05$, $^{**}p < 0.01$, $^{***}p < 0.001$.

S3.6.5 Partisanship

Fig. S9 plots AMEs of proximity quintiles by partisanship. For recognition, Republicans show a positive Q1 effect for renewable energy projects but not for manufacturing, whereas Independents show a positive Q1 effect for manufacturing with null effects for renewables. Table S23 reports the underlying estimates. These subgroup AMEs are positive in the cases noted, but cross-party differences are imprecisely estimated and cannot rule out zero difference.

For credit attribution to President Biden, there is no consistent proximity effect within any partisan group.

For perceived economic benefits, Republicans living nearer to projects report lower benefits than Democrats at similar distances. Within Republicans, however, proximity itself shows no detectable gradient; the Q1–Q4 contrasts relative to Q5 are near zero and not precisely estimated.

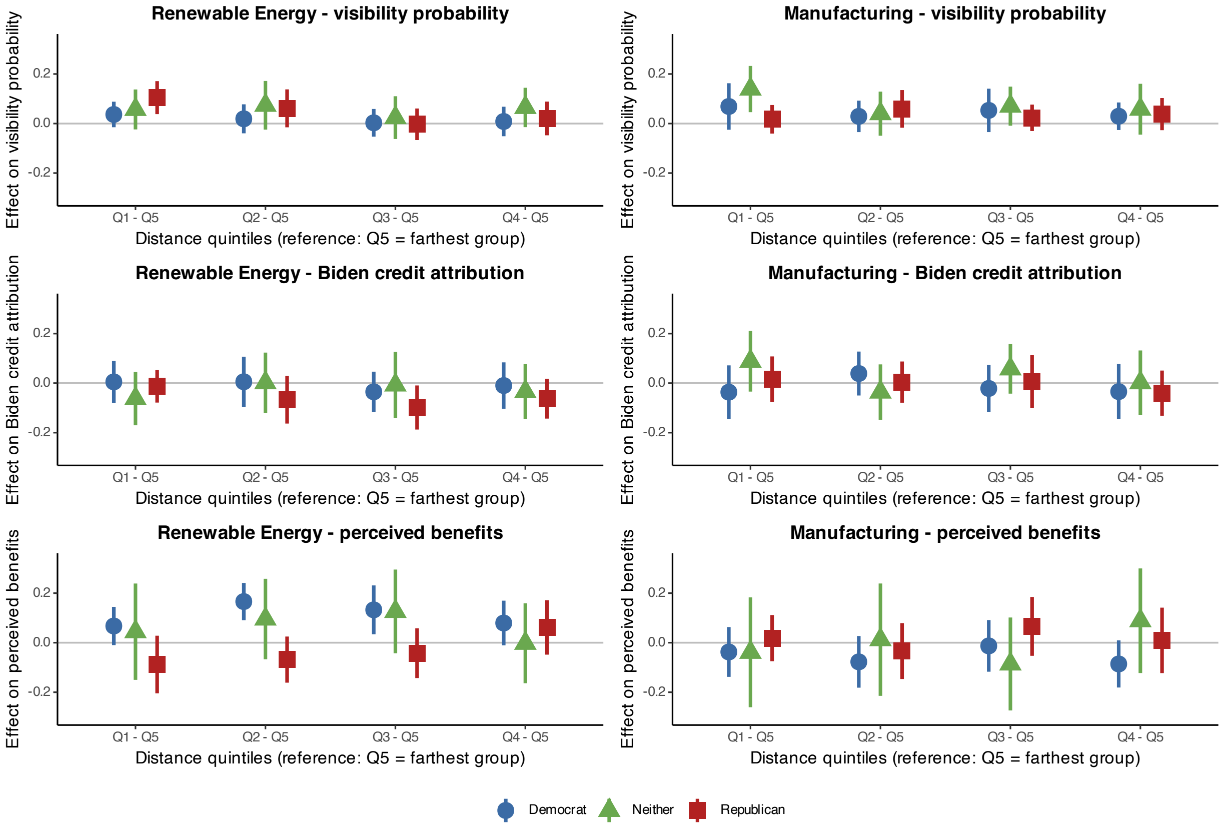

Fig. S9: Heterogeneous effects of proximity by respondent partisan identification. Plot depicts the average marginal effect of a proximity quintile (relative to Q5) across moderator values. Bars denote 95% confidence intervals with cluster-robust standard errors by state. Equation S1 describes the model specification. Table S23 reports interaction terms and sample sizes.

Table S23: Heterogeneous proximity effects by partisanship

	Renewable Energy			Manufacturing		
	Visibility	Credit	Benefit	Visibility	Credit	Benefit
Q1 proximity	0.037	0.0051	0.067	0.069	-0.036	-0.037
	(0.026)	(0.0432)	(0.039)	(0.048)	(0.055)	(0.051)
Q2 proximity	0.019	0.0055	0.166***	0.029	0.039	-0.077
	(0.030)	(0.0516)	(0.038)	(0.032)	(0.045)	(0.053)
Q3 proximity	0.003	-0.035	0.13*	0.053	-0.022	-0.013
	(0.028)	(0.041)	(0.05)	(0.045)	(0.048)	(0.053)
Q4 proximity	0.0083	-0.0098	0.079	0.029	-0.034	-0.086
	(0.0304)	(0.0477)	(0.046)	(0.028)	(0.057)	(0.049)
Neither	-0.100*	-0.208***	-0.06	-0.096*	-0.259***	-0.140
	(0.046)	(0.047)	(0.07)	(0.038)	(0.059)	(0.081)
Republican	-0.033	-0.127**	-0.030	-0.0014	-0.179***	-0.200**
	(0.029)	(0.037)	(0.048)	(0.0337)	(0.042)	(0.057)
Q1 × Neither	0.020	-0.068	-0.023	0.07	0.125	-0.0014
	(0.052)	(0.059)	(0.100)	(0.06)	(0.082)	(0.1143)
Q1 × Republican	0.068	-0.018	-0.156*	-0.052	0.053	0.056
	(0.039)	(0.054)	(0.069)	(0.050)	(0.057)	(0.065)
Q2 × Neither	0.055	-0.0039	-0.071	0.011	-0.075	0.09
	(0.056)	(0.0676)	(0.084)	(0.051)	(0.079)	(0.12)
Q2 × Republican	0.042	-0.072	-0.234***	0.030	-0.035	0.044
	(0.039)	(0.069)	(0.061)	(0.045)	(0.054)	(0.087)
Q3 × Neither	0.021	0.028	-0.0063	0.017	0.079	-0.073
	(0.045)	(0.070)	(0.0879)	(0.045)	(0.072)	(0.094)
Q3 × Republican	-0.006	-0.063	-0.175*	-0.030	0.028	0.079
	(0.043)	(0.058)	(0.068)	(0.056)	(0.054)	(0.079)
Q4 × Neither	0.057	-0.025	-0.082	0.028	0.036	0.175
	(0.058)	(0.062)	(0.093)	(0.052)	(0.101)	(0.098)
Q4 × Republican	0.012	-0.053	-0.018	0.0086	-0.0061	0.095
	(0.048)	(0.060)	(0.072)	(0.0442)	(0.0777)	(0.071)
N	5026	3034	1487	5026	3034	1487
Adjusted R^2	0.075	0.067	0.187	0.074	0.068	0.180
Covariates	Yes	Yes	Yes	Yes	Yes	Yes
State Fixed Effects	Yes	Yes	Yes	Yes	Yes	Yes

Notes: Each column reports a separate linear probability model with interactions between proximity and partisanship. Unit of analysis is the individual survey respondent. Visibility = 1 if respondent reports a local green project, 0 otherwise. Credit = 1 if respondent credits the Biden Administration for local green investments. Benefit = 1 if respondent perceives a benefit from local green projects. Estimates are OLS with cluster-robust standard errors by state in parentheses. $^{*}p < 0.05$, $^{**}p < 0.01$, $^{***}p < 0.001$.

S3.6.6 Education

Fig. S10 reports the effects of proximity for respondents with and without a 4-year college degree. Table S24 contains the interaction terms. There are no consistent differences in proximity's effects by respondent education level.

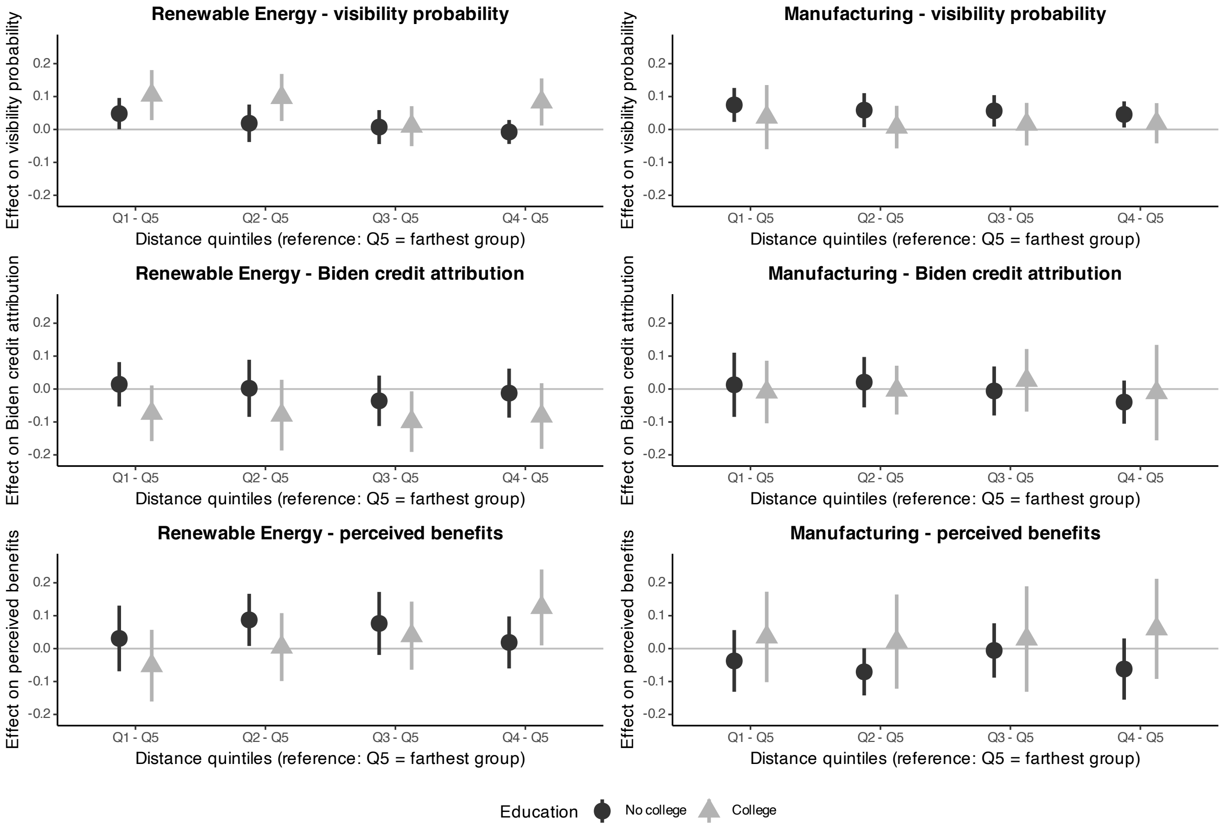

Fig. S10: Heterogeneous effects of proximity by respondent education. Plot depicts the average marginal effect of a proximity quintile (relative to Q5) across moderator values. Bars denote 95% confidence intervals with cluster-robust standard errors by state. Equation S1 describes the model specification. Table S24 reports interaction terms and sample sizes.

Table S24: Heterogeneous proximity effects by no college vs. college

	Renewable Energy			Manufacturing		
	Visibility	Credit	Benefit	Visibility	Credit	Benefit
Q1 proximity	0.048	0.014	0.031	0.075**	0.013	-0.037
	(0.024)	(0.034)	(0.051)	(0.026)	(0.050)	(0.048)
Q2 proximity	0.019	0.0022	0.087*	0.059*	0.021	-0.071
	(0.029)	(0.0443)	(0.040)	(0.026)	(0.039)	(0.037)
Q3 proximity	0.0072	-0.036	0.077	0.056*	-0.0059	-0.0056
	(0.0263)	(0.039)	(0.049)	(0.024)	(0.0380)	(0.0422)
Q4 proximity	-0.0076	-0.012	0.019	0.046*	-0.040	-0.062
	(0.0186)	(0.038)	(0.040)	(0.020)	(0.033)	(0.047)
College	0.026	0.110*	0.015	0.101***	0.046	-0.062
	(0.032)	(0.049)	(0.054)	(0.026)	(0.039)	(0.058)
Q1 × College	0.056	-0.088	-0.083	-0.037	-0.022	0.073
	(0.048)	(0.058)	(0.076)	(0.043)	(0.066)	(0.084)
Q2 × College	0.078	-0.081	-0.083	-0.051	-0.024	0.092
	(0.040)	(0.067)	(0.071)	(0.033)	(0.049)	(0.077)
Q3 × College	0.0028	-0.063	-0.037	-0.041	0.032	0.035
	(0.0407)	(0.062)	(0.072)	(0.033)	(0.045)	(0.088)
Q4 × College	0.091*	-0.070	0.106	-0.027	0.029	0.122
	(0.041)	(0.061)	(0.069)	(0.029)	(0.092)	(0.071)
N	5026	3034	1487	5026	3034	1487
Adjusted R^2	0.076	0.068	0.185	0.073	0.067	0.179
Covariates	Yes	Yes	Yes	Yes	Yes	Yes
State Fixed Effects	Yes	Yes	Yes	Yes	Yes	Yes

Notes: Each column reports a separate linear probability model with interactions between proximity and no college vs. college. Unit of analysis is the individual survey respondent. Visibility = 1 if respondent reports a local green project, 0 otherwise. Credit = 1 if respondent credits the Biden Administration for local green investments. Benefit = 1 if respondent perceives a benefit from local green projects. Estimates are OLS with cluster-robust standard errors by state in parentheses. $^{*}p < 0.05$, $^{**}p < 0.01$, $^{***}p < 0.001$.

S3.6.7 Household Income

Fig. S11 reports the average marginal effect of proximity on the outcomes depending on whether the respondent's household income is above or below the national median. Table S25 contains the interaction terms. There are no consistent differences in proximity's effect on the outcomes across income levels.

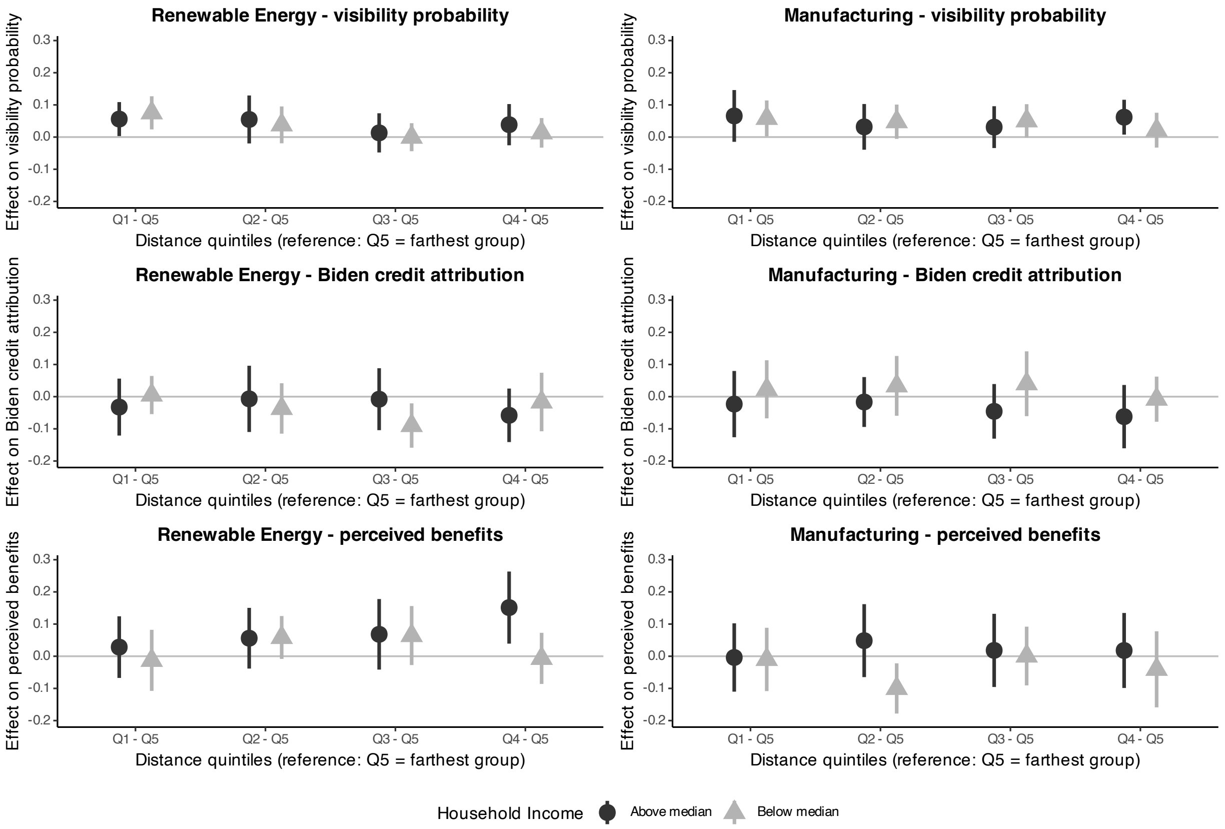

Fig. S11: Heterogeneous effects of proximity by respondent income. Plot depicts the average marginal effect of a proximity quintile (relative to Q5) across moderator values. Bars denote 95% confidence intervals with cluster-robust standard errors by state. Equation S1 describes the model specification. Table S25 reports interaction terms and sample sizes.

Table S25: Heterogeneous proximity effects by household income

	Renewable Energy			Manufacturing		
	Visibility	Credit	Benefit	Visibility	Credit	Benefit
Q1 proximity	0.056*	-0.032	0.028	0.066	-0.023	-0.0039
	(0.027)	(0.045)	(0.049)	(0.041)	(0.053)	(0.0542)
Q2 proximity	0.055	-0.0069	0.056	0.032	-0.017	0.048
	(0.038)	(0.0525)	(0.048)	(0.036)	(0.040)	(0.058)
Q3 proximity	0.013	-0.0079	0.068	0.031	-0.046	0.018
	(0.031)	(0.0491)	(0.056)	(0.033)	(0.043)	(0.058)
Q4 proximity	0.039	-0.058	0.151*	0.062*	-0.062	0.018
	(0.033)	(0.042)	(0.057)	(0.028)	(0.050)	(0.059)
Below median	-0.030	0.015	0.075	-0.036	-0.035	0.079
	(0.034)	(0.055)	(0.063)	(0.029)	(0.058)	(0.060)
Q1 × Below median	0.019	0.037	-0.041	-0.0076	0.046	-0.0061
	(0.036)	(0.053)	(0.061)	(0.0345)	(0.064)	(0.0699)
Q2 × Below median	-0.017	-0.030	0.0023	0.016	0.050	-0.149*
	(0.043)	(0.057)	(0.0559)	(0.041)	(0.065)	(0.065)
Q3 × Below median	-0.014	-0.082	-0.0038	0.020	0.086	-0.017
	(0.034)	(0.058)	(0.0704)	(0.038)	(0.065)	(0.064)
Q4 × Below median	-0.025	0.041	-0.16*	-0.040	0.054	-0.059
	(0.042)	(0.065)	(0.07)	(0.038)	(0.064)	(0.078)
N	5026	3034	1487	5026	3034	1487
Adjusted R^2	0.075	0.069	0.184	0.074	0.067	0.181
Covariates	Yes	Yes	Yes	Yes	Yes	Yes
State Fixed Effects	Yes	Yes	Yes	Yes	Yes	Yes

Notes: Each column reports a separate linear probability model with interactions between proximity and household income. Unit of analysis is the individual survey respondent. Visibility = 1 if respondent reports a local green project, 0 otherwise. Credit = 1 if respondent credits the Biden Administration for local green investments. Benefit = 1 if respondent perceives a benefit from local green projects. Estimates are OLS with cluster-robust standard errors by state in parentheses. $^{*}p < 0.05$, $^{**}p < 0.01$, $^{***}p < 0.001$.

S3.6.8 Biden Election Exit

Fig. S12 reports the average marginal effect of proximity on the outcomes depending on whether the respondent answered the survey before or after President Biden withdrew his candidacy. Questions about potential benefits weren't asked both before and after Biden dropped out. Table S26 contains the interaction terms. There are no consistent differences in proximity's effect on the outcomes before and after Biden exited the presidential race.

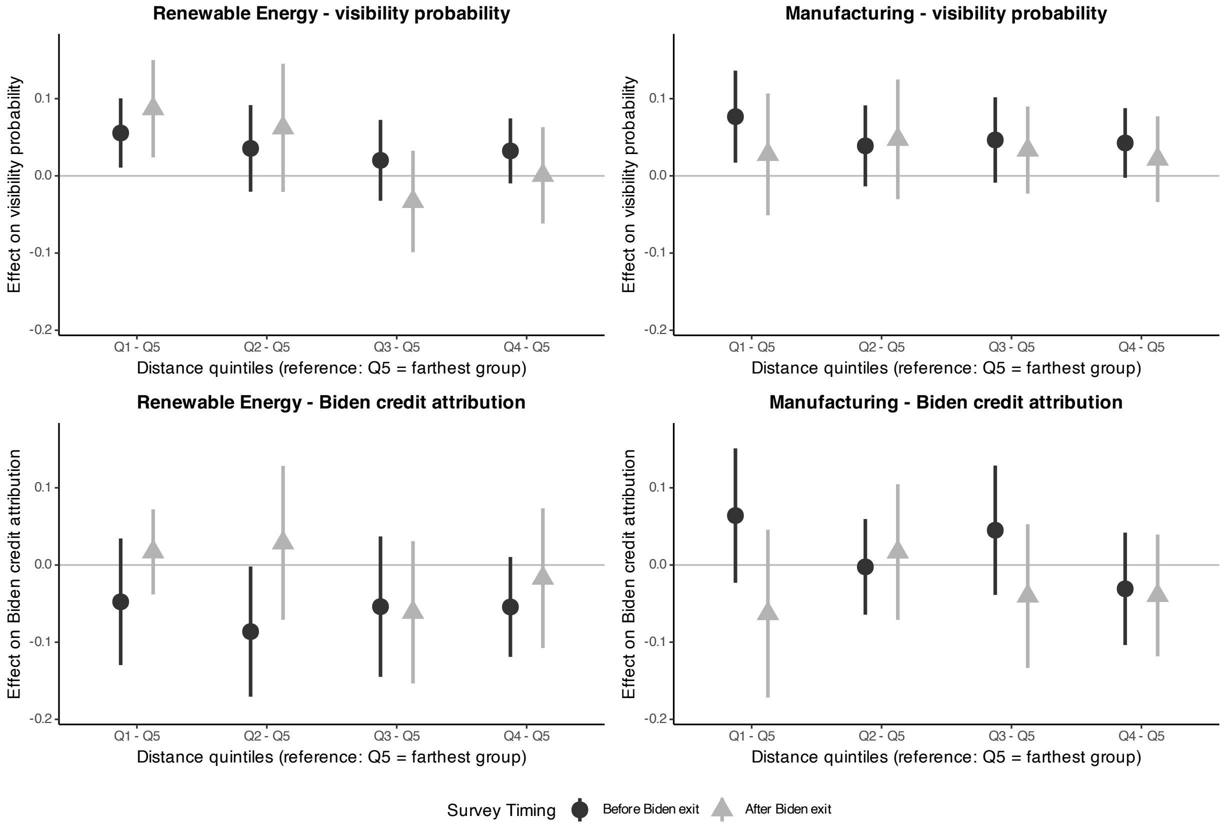

Fig. S12: Heterogeneous effects of proximity by respondent survey date relative to Biden exiting the presidential race. Plot depicts the average marginal effect of a proximity quintile (relative to Q5) across moderator values. Bars denote 95% confidence intervals with cluster-robust standard errors by state. Equation S1 describes the model specification. Table S26 reports interaction terms and sample sizes.

Table S26: Heterogeneous proximity effects by before biden exit vs. after biden exit

	Renewable Energy		Manufacturing	
	Visibility	Credit	Visibility	Credit
Q1 proximity	0.056*	-0.048	0.077*	0.064
	(0.023)	(0.042)	(0.030)	(0.044)
Q2 proximity	0.036	-0.086	0.039	-0.0024
	(0.029)	(0.043)	(0.027)	(0.0316)
Q3 proximity	0.020	-0.054	0.046	0.045
	(0.027)	(0.046)	(0.028)	(0.043)
Q4 proximity	0.032	-0.054	0.043	-0.031
	(0.021)	(0.033)	(0.023)	(0.037)
After Biden exit	0.023	-0.044	0.033	0.041
	(0.029)	(0.042)	(0.024)	(0.034)
Q1 × After Biden exit	0.031	0.065	-0.049	-0.127
	(0.038)	(0.047)	(0.035)	(0.063)
Q2 × After Biden exit	0.027	0.115	0.0084	0.019
	(0.047)	(0.063)	(0.0432)	(0.046)
Q3 × After Biden exit	-0.053	-0.0073	-0.013	-0.085
	(0.045)	(0.0716)	(0.038)	(0.054)
Q4 × After Biden exit	-0.032	0.037	-0.021	-0.0086
	(0.039)	(0.048)	(0.032)	(0.0515)
N	5026	3034	5026	3034
Adjusted R^2	0.076	0.069	0.073	0.070
Covariates	Yes	Yes	Yes	Yes
State Fixed Effects	Yes	Yes	Yes	Yes

Notes: Each column reports a separate linear probability model with interactions between proximity and before biden exit vs. after biden exit. Unit of analysis is the individual survey respondent. Visibility = 1 if respondent reports a local green project, 0 otherwise. Credit = 1 if respondent credits the Biden Administration for local green investments. Estimates are OLS with cluster-robust standard errors by state in parentheses. $^{*}p < 0.05$, $^{**}p < 0.01$, $^{***}p < 0.001$.

S4 Regression of Perceived Benefits on Covariates

Table S27 reports the results from a linear regression of the perceived benefits indicator on covariates. Model 1 includes the covariates from the primary model specification in addition to the recognition indicator. Model 2 interacts the recognition indicator with respondent partisanship. Model 3 interacts the recognition indicator with the 2020 Biden two-party vote share for the respondent's county. The intention of Models 2-3 is to see if visibility's correlation with perceived benefits varies with measures of individual partisanship and local political context.

Table S27: Linear probability model of perceived project benefits

	(1)	(2)	(3)
Intercept	0.12	0.16	0.11
	(0.21)	(0.22)	(0.21)
Age	0.00034	0.00041	0.00031
	(0.00106)	(0.00105)	(0.00107)
Female	-0.044*	-0.044*	-0.044*
	(0.020)	(0.020)	(0.020)
Black	-0.041	-0.044	-0.042
	(0.032)	(0.032)	(0.031)
Asian	-0.034	-0.029	-0.034
	(0.067)	(0.066)	(0.067)
Other race	0.061	0.061	0.060
	(0.044)	(0.046)	(0.045)
Hispanic/Latino	-0.083*	-0.086*	-0.083*
	(0.035)	(0.035)	(0.035)
College	0.0068	0.008	0.0069
	(0.0228)	(0.024)	(0.0228)
Employed	0.054	0.056	0.053
	(0.035)	(0.035)	(0.035)
Income Q2	0.030	0.029	0.029
	(0.025)	(0.026)	(0.025)
Income Q3	0.0072	0.0051	0.0058
	(0.0308)	(0.0304)	(0.0316)
Income Q4	0.043	0.042	0.041
	(0.035)	(0.034)	(0.035)
Income Q5	0.066	0.070	0.066
	(0.039)	(0.039)	(0.039)
Republican	-0.141***	-0.188***	-0.141***
	(0.032)	(0.039)	(0.032)
Neither party	-0.103**	-0.121**	-0.102**
	(0.035)	(0.043)	(0.035)
Global warming index	0.589***	0.575***	0.588***
	(0.046)	(0.049)	(0.047)
Population density	0.012	0.010	0.012
	(0.015)	(0.016)	(0.015)
County college share $(t-1)$	0.019	0.021	0.021
	(0.025)	(0.024)	(0.025)
County poverty share $(t-1)$	-0.00066	0.0026	0.00022
	(0.01627)	(0.0164)	(0.01636)
County foreign-born share $(t-1)$	-0.0018	-0.0017	-0.0019
	(0.0142)	(0.0141)	(0.0142)
Median county housing costs $(t-1)$	0.0027	0.0031	0.0027
	(0.0113)	(0.0113)	(0.0113)
Faster broadband access $(t-1)$	0.050	0.049	0.048
	(0.027)	(0.027)	(0.027)
County GDP (log) $(t-1)$	0.031	0.020	0.030
	(0.059)	(0.061)	(0.059)
Labor force (log) $(t-1)$	-0.042	-0.032	-0.042
	(0.049)	(0.052)	(0.049)
County unemployment rate $(t-1)$	0.019	0.017	0.019
	(0.011)	(0.011)	(0.011)
Highway access	-0.0095	-0.013	-0.010
	(0.0329)	(0.035)	(0.033)
County income pc $(t-1)$	-0.012	-0.0085	-0.012
	(0.020)	(0.0193)	(0.020)
State electricity price $(t-1)$	0.007	0.0059	0.0072
	(0.012)	(0.0128)	(0.0126)
State unionization rate $(t-1)$	-0.0282**	-0.0284**	-0.0279**
	(0.0087)	(0.0091)	(0.0088)
Visible green project	-0.036	-0.101**	0.025
	(0.025)	(0.033)	(0.097)
Visibility x Neither party		0.051	
		(0.075)	
Visibility x Republican		0.165*	
		(0.063)	
Visibility x 2020 county Biden share			-0.016
			(0.025)
N	1488	1488	1488
Adjusted R^2	0.193	0.197	0.193
Sample Fixed Effects	No	No	No
State Fixed Effects	No	No	No

Notes: Unit of analysis is the individual survey respondent. Dependent variable = 1 if respondent perceives benefits from local green energy projects, 0 otherwise. Estimates are OLS with cluster-robust standard errors by state. Continuous covariates are standardized: county-level variables use within-state standardization; state-level variables use full-sample z-scores; individual-level indices are standardized by construction. * $p < 0.05$, ** $p < 0.01$, *** $p < 0.001$.

S5 Regression of Credit Attribution on Covariates

Table S28: Linear probability models of credit attribution

	Credit Recipient:					
	Biden	Congress	Governor	State	Local	Markets
Intercept	0.063	0.075	0.29	0.41*	0.17	0.12
	(0.191)	(0.166)	(0.20)	(0.16)	(0.14)	(0.17)
Age	0.00068	-0.00289***	0.00036	-0.00069	-0.00104	-0.00035
	(0.00058)	(0.00054)	(0.00059)	(0.00054)	(0.00053)	(0.00051)
Female	-0.053**	-0.089***	-0.029	-0.044*	-0.013	-0.101***
	(0.018)	(0.015)	(0.023)	(0.018)	(0.021)	(0.019)
Black	0.037	0.075**	0.047	0.057	0.027	0.060*
	(0.028)	(0.028)	(0.032)	(0.030)	(0.031)	(0.026)
Asian	0.024	0.053	0.012	0.052	-0.075	-0.080*
	(0.044)	(0.049)	(0.043)	(0.055)	(0.049)	(0.035)
Other race	-0.031	0.027	0.041	0.020	0.069*	-0.016
	(0.029)	(0.039)	(0.035)	(0.029)	(0.033)	(0.030)
Hispanic/Latino	-0.0029	-0.0064	-0.014	-0.042*	-0.0042	0.0083
	(0.0180)	(0.0212)	(0.025)	(0.019)	(0.0188)	(0.0203)
College	0.038	0.035	-0.0081	-0.0067	-0.0011	0.058***
	(0.021)	(0.022)	(0.0272)	(0.0156)	(0.0166)	(0.016)
Employed	0.029	0.043	0.021	0.041*	0.031	0.054*
	(0.018)	(0.022)	(0.021)	(0.020)	(0.021)	(0.025)
Income Q2	-0.015	-0.021	-0.010	-0.031	0.032	-0.0051
	(0.026)	(0.030)	(0.023)	(0.024)	(0.023)	(0.0298)
Income Q3	-0.045	-0.0072	0.024	0.044	0.032	-0.023
	(0.027)	(0.0277)	(0.023)	(0.024)	(0.022)	(0.029)
Income Q4	-0.028	-0.0056	0.037	0.012	0.040	-0.027
	(0.028)	(0.0287)	(0.030)	(0.021)	(0.028)	(0.031)
Income Q5	-0.040	-0.015	0.123***	0.084**	0.098*	0.040
	(0.033)	(0.034)	(0.032)	(0.027)	(0.039)	(0.038)
Republican	-0.181***	-0.0071	-0.13***	-0.084**	-0.088***	0.014
	(0.025)	(0.0284)	(0.03)	(0.026)	(0.023)	(0.034)
Neither party	-0.213***	-0.106**	-0.181***	-0.139***	-0.123***	-0.026
	(0.026)	(0.036)	(0.042)	(0.036)	(0.033)	(0.043)
Global warming index	0.054	0.077	0.130***	0.094*	0.148***	0.051
	(0.037)	(0.039)	(0.033)	(0.042)	(0.039)	(0.038)
Population density	0.0117	0.0158	-0.010	-0.0015	0.006	0.0228**
	(0.0068)	(0.0088)	(0.011)	(0.0138)	(0.013)	(0.0078)
County college share $(t-1)$	-0.016	-0.02	0.003	0.017	0.014	0.00034
	(0.019)	(0.02)	(0.019)	(0.024)	(0.017)	(0.01962)
County poverty share $(t-1)$	-0.0042	0.0055	-0.010	0.011	0.0088	0.01
	(0.0122)	(0.0098)	(0.015)	(0.011)	(0.0103)	(0.01)
County foreign-born share $(t-1)$	-0.0045	0.0179	0.019	0.0199*	0.013	-0.0055
	(0.0115)	(0.0094)	(0.012)	(0.0083)	(0.011)	(0.0120)
Median county housing costs $(t-1)$	0.017	0.0062	-0.0091	-0.021	-0.00079	-0.012
	(0.012)	(0.0148)	(0.0186)	(0.013)	(0.01853)	(0.013)
Faster broadband access $(t-1)$	-0.039	-0.042	-0.020	0.033	-0.0023	0.0086
	(0.020)	(0.025)	(0.022)	(0.031)	(0.0232)	(0.0205)
County GDP (log) $(t-1)$	0.054	0.131**	0.027	-0.022	0.023	-0.0077
	(0.040)	(0.046)	(0.052)	(0.037)	(0.048)	(0.0513)
Labor force (log) $(t-1)$	-0.048	-0.126**	-0.033	-0.00041	-0.031	0.013
	(0.038)	(0.045)	(0.045)	(0.03403)	(0.048)	(0.045)
County unemployment rate $(t-1)$	0.0167*	0.0050	0.0170*	0.0102	0.0053	0.0137
	(0.0073)	(0.0076)	(0.0075)	(0.0083)	(0.0087)	(0.0083)
Highway access	0.069	0.0036	-0.010	-0.0095	0.039	0.015
	(0.041)	(0.0316)	(0.037)	(0.0324)	(0.035)	(0.030)
County income pc $(t-1)$	-0.0091	-0.016	0.0082	0.031	-0.0021	0.024
	(0.0132)	(0.014)	(0.0172)	(0.025)	(0.0153)	(0.019)
Republican Governor	0.044	0.0071	-0.166***	-0.065*	0.0043	0.061
	(0.022)	(0.0241)	(0.031)	(0.028)	(0.0232)	(0.031)
State electricity price $(t-1)$	-0.018*	-0.021*	-0.011	0.0019	-0.010	0.017
	(0.008)	(0.010)	(0.015)	(0.0117)	(0.014)	(0.012)
State unionization rate $(t-1)$	-0.0025	0.0038	0.016	0.023*	0.013	-0.014
	(0.0078)	(0.0092)	(0.012)	(0.010)	(0.011)	(0.011)
GOP Gov. × Republican	0.029	0.025	0.244***	0.138***	0.081*	-0.0098
	(0.030)	(0.034)	(0.041)	(0.036)	(0.035)	(0.0456)
GOP Gov. × Neither party	0.0016	0.06	0.166**	0.089	0.057	-0.040
	(0.0484)	(0.05)	(0.057)	(0.046)	(0.047)	(0.049)
Visible green project	0.13***	0.149***	0.13***	0.135***	0.184***	0.174***
	(0.02)	(0.019)	(0.02)	(0.023)	(0.017)	(0.015)
N	3034	3034	3034	3034	3034	3034
Adjusted R^2	0.084	0.098	0.066	0.066	0.072	0.073
Sample Fixed Effects	Yes	Yes	Yes	Yes	Yes	Yes

Notes: Each column reports a separate linear probability model. Unit of analysis is the individual survey respondent. Dependent variable = 1 if respondent credits the column header actor for local green investments, 0 otherwise. Continuous covariates are standardized: county-level variables use within-state standardization; state-level variables use full-sample z-scores; individual-level indices are standardized by construction. Estimates are OLS with cluster-robust standard errors by state in parentheses. * $p < 0.05$, ** $p < 0.01$, *** $p < 0.001$.

Table S29: Linear probability models of within-subject credit attribution

	Credit Biden but not the...	
	Governor	State
Intercept	0.12	0.16
	(0.12)	(0.15)
Age	0.00081	0.00084
	(0.00059)	(0.00061)
Female	-0.012	-0.017
	(0.015)	(0.011)
Black	-0.0046	-0.010
	(0.0195)	(0.023)
Asian	0.0061	-0.030
	(0.0201)	(0.022)
Other race	-0.011	-0.016
	(0.019)	(0.020)
Hispanic/Latino	-0.0038	0.0066
	(0.0186)	(0.0163)
College	0.013	0.018
	(0.014)	(0.010)
Employed	-0.00032	-0.0049
	(0.01293)	(0.0163)
Income Q2	0.0076	-0.0026
	(0.0187)	(0.0243)
Income Q3	-0.005	-0.033
	(0.016)	(0.020)
Income Q4	-0.024	-0.021
	(0.025)	(0.022)
Income Q5	-0.080**	-0.081**
	(0.027)	(0.027)
Republican	-0.053***	-0.077***
	(0.013)	(0.019)
Neither party	-0.048**	-0.076**
	(0.018)	(0.023)
Global warming index	-0.018	-0.010
	(0.023)	(0.034)
Population density	0.0111	0.0027
	(0.0077)	(0.0098)
County college share $(t-1)$	-0.0069	-0.014
	(0.0112)	(0.012)
County poverty share $(t-1)$	-0.0027	-0.0083
	(0.0113)	(0.0101)
County foreign-born share $(t-1)$	-0.013	-0.0079
	(0.012)	(0.0076)
Median county housing costs $(t-1)$	0.013	0.023*
	(0.011)	(0.011)
Faster broadband access $(t-1)$	-0.0032	-0.026
	(0.0156)	(0.017)
County GDP (log) $(t-1)$	-0.021	-0.0025
	(0.030)	(0.0281)
Labor force (log) $(t-1)$	0.021	0.015
	(0.028)	(0.024)
County unemployment rate $(t-1)$	0.0027	0.0025
	(0.0057)	(0.0064)
Highway access	0.044	0.043
	(0.023)	(0.025)
County income pc $(t-1)$	-0.0025	-0.020
	(0.0103)	(0.013)
Republican Governor	0.148***	0.063**
	(0.021)	(0.021)
State electricity price	0.00082	-0.0023
	(0.00159)	(0.0013)
State unionization rate	-0.16	-0.55*
	(0.22)	(0.24)
GOP Gov. × Republican	-0.055*	-0.024
	(0.024)	(0.027)
GOP Gov. × Neither party	-0.095**	-0.049
	(0.030)	(0.035)
Visible green project	0.0065	-0.0013
	(0.0149)	(0.0194)
N	3034	3034
Adjusted R^2	0.035	0.024
Sample Fixed Effects	Yes	Yes

Notes: Each column reports a separate linear probability model. Unit of analysis is the individual survey respondent. Dependent variable = 1 if the respondent credits the column header actor but does not credit President Biden, 0 otherwise. Continuous covariates are standardized: county-level variables use within-state standardization; state-level variables use full-sample z-scores. Estimates are OLS with cluster-robust standard errors by state in parentheses. * $p < 0.05$, ** $p < 0.01$, *** $p < 0.001$.

S6 Statement Analyses

S6.1 Statement Type Description

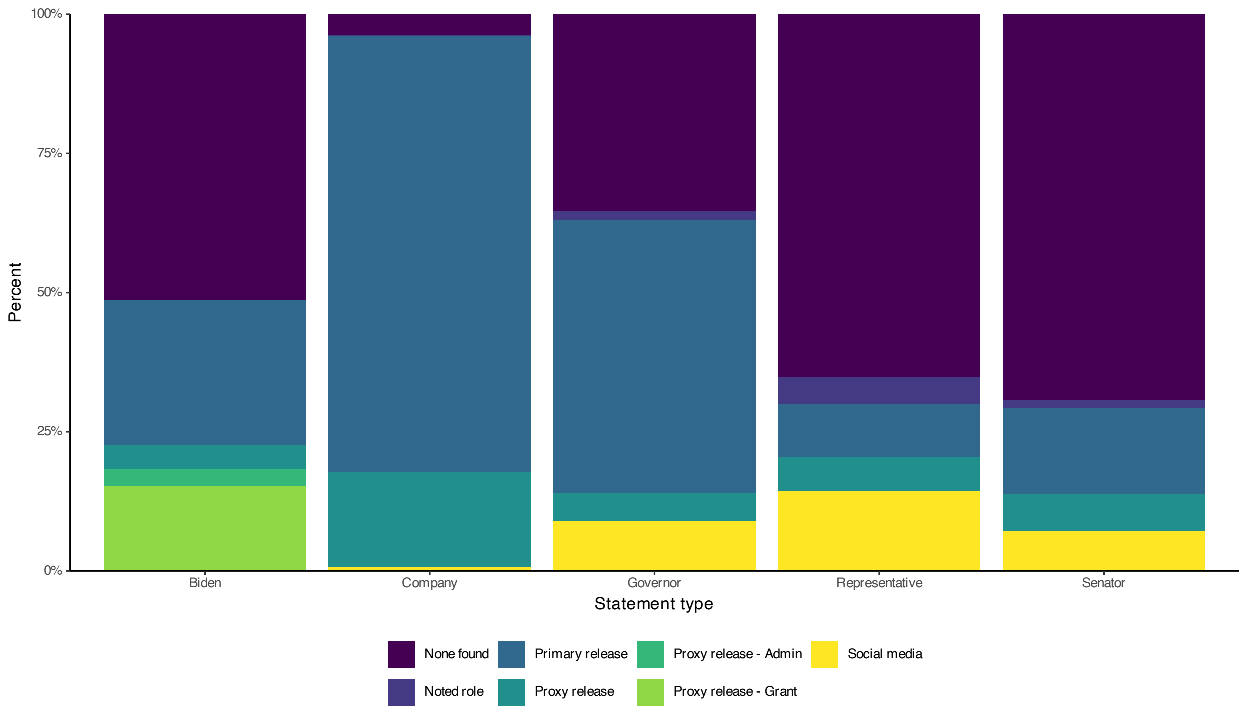

Fig. S13: Types of statements by actor. Categories are mutually exclusive and indicate the highest-quality source found for each actor–project pair. *None found*: No statement located. *Primary release*: Official communication (press release, newsletter, transcript, or report) issued on the actor's website. *Social media*: Posts on X, Facebook, Instagram, or LinkedIn, used only if no primary release exists. *Proxy release*: Statements about the project appearing only in a news article or another actor's release. For Biden, proxy releases are subdivided into *Grant* (project appears in a grant-specific administration release) and *Admin* (statement by a senior official speaking for the administration). *Noted role*: No direct statement found, but actor involvement is documented (e.g., groundbreaking attendance, executive meeting, or mention in a grant application).

S6.2 Summary Statistics

Table S30: Summary statistics for statement analysis covariates

	Mean	SD	Min	Max	Missing
Statement character count	3668	4547	85	38189	0
Missing location (FIPS)	0.055	0.228	0	1	0
Sector: EVs	0.193	0.394	0	1	0
Sector: Wind	0.086	0.28	0	1	0
Sector: Solar	0.235	0.424	0	1	0
Sector: Batteries	0.486	0.5	0	1	0
Target jobs specified	0.737	0.44	0	1	0
Capital investment	0.755	0.43	0	1	0
Status: Cancelled/Closed/Paused/Sold/Rumored	0.165	0.371	0	1	0
Status: Pilot/Planned/Construction	0.599	0.49	0	1	0
Status: Operating	0.235	0.424	0	1	0
Manufacturing project	0.804	0.397	0	1	0
Highway access	0.835	0.371	0	1	108
Broadband 100+ Mbps	0.485	0.5	0	1	108
Swing state	0.242	0.428	0	1	0
Competitive district	0.075	0.263	0	1	120
College education (z)	0.005	0.965	-1.973	2.612	108
Poverty rate (z)	0.014	0.96	-1.908	3.254	108
GDP log (z)	0.125	1.037	-2.547	2.419	108
Unemployment rate (z)	0.05	0.978	-1.729	6.64	108
Labor force log (z)	0.114	1.043	-3.139	2.335	108
Income per capita (z)	0	0.909	-1.228	9.086	108
Democratic vote share (z)	0.07	0.973	-2.245	2.798	108
Foreign-born population (z)	0.093	1.03	-1.024	3.77	108
Housing costs (z)	0.043	0.962	-2.064	3.745	108
Electricity price (z)	-0.042	0.921	-1.004	4.378	0
Union membership (z)	-0.02	1.026	-1.244	2.764	0
Party: Democrat	0.426	0.495	0	1	56
Party: Republican	0.574	0.495	0	1	56
U.S. Rep. Party: Democrat	0.349	0.477	0	1	20
U.S. Rep. Party: Republican	0.651	0.477	0	1	20
Governor Party: Democrat	0.429	0.496	0	1	12
Governor Party: Republican	0.571	0.496	0	1	12

Notes: Summary statistics for covariates used in statement regression models. Statement character count is calculated only for observations with statements. Continuous and dummy variables show mean, standard deviation, minimum, maximum, and missing values. Categorical variables are split into dummy variables (0/1) for each category. Standardized variables (z) are standardized across unique counties (each county weighted equally), so means in this table may differ from zero due to counties with more statements receiving greater weight. Party variables are calculated on applicable subsets: speaker party excludes companies and President; representative party includes only U.S. Rep. observations; governor party includes only governor observations. Full sample $N = 1962$; elected officials (excl. Company/Biden) $N = 1308$; representatives $N = 327$; governors $N = 327$.

S6.3 LLM Annotation

S6.3.1 Stage 1 Prompt

You are a binary classifier. Output `YES` or `NO` only.

Answer `YES` only if the statement **explicitly** indicates that *the Inflation Reduction Act / the Bipartisan Infrastructure Law*:

1. Directly funded or financed the specific project being discussed, **OR**
2. Directly enabled or made possible the specific project through incentives, **OR**
3. Is explicitly cited as contributing to the investment decision, including when:
   - The law's economic impact is cited as a factor in the decision
   - The law's industry growth effects influenced the choice
   - The law's broader benefits are linked to this specific investment

The statement must show a **causal link** between the law and **this specific project**.

Do *not* answer `YES` if:

- The law is only mentioned as a goal or target
- The project helps meet the law's goals
- The law is mentioned only as background without influencing decisions
- The statement only discusses eligibility without confirming use
- The speaker only mentions helping to write the law

S6.3.2 Stage 2 Prompt

Credit Attribution Codebook

1. First Check: Is there any credit claim? (`gives_credit`)
Decision tree for `gives_credit`:

1. Explicit credit:
   a) Causal verbs (enabled, secured, funded), OR
   b) Attribution of decision-making ("contributes to our decision", "influenced by"), OR
   c) Economic environment claims ("thanks to", "because of", "due to")
   → If YES to any, set `gives_credit=1` and continue to Step 2
   → If NO, continue to Question 2
2. Implicit credit (check all):
   - Actor attends/hosts ceremony for project
   - Actor announces project and frames it as achievement
   - Actor publicly associates with project success

   → If YES to any, set `gives_credit=1` and continue to Step 2
   → If NO, continue to Question 3
3. Merely descriptive/informative (check all):

- Technical specifications or equipment lists
- Routine business updates
- Factual job numbers without attribution
- Boilerplate text

→ If YES to any, set all variables to 0 and STOP

Key distinction: Credit includes both direct causation ("funded by") *and* attribution of influence ("contributed to our decision").
Examples of NO credit:

- "New factory will create 500 jobs" (just reporting)
- "Company X announced plans to expand" (passive description)
- "The IRA sets ambitious goals" (mere mention)

Examples of YES credit:

- "Our state attracted this investment" (active role)
- "Thanks to our business climate..." (explicit attribution)
- "The IRA's impact on industrial growth contributed to our decision" (policy impact attribution)

2. Who Gets Credit? (if `gives_credit=1`)
Social media rules:

- Credit if @mention in success/achievement context
- Credit for "partnership with @Actor", "working with @Actor", "thanks to @Actor"
- No credit for cc's, FYIs, requests, or complaints

Federal actors:

- `credit_biden=1` if President/White House named or tagged with credit
- `credit_senate=1` if specific U.S. Senator credited
- `credit_us_rep=1` if specific U.S. Representative credited

State & local actors:

- `credit_governor=1` if Governor named/quoted with credit
- `credit_local=1` if local government credited (support, recruitment, incentives)

Party & laws:

- `credit_dem/credit_gop=1` if explicit partisan attribution
- `credit_ira/credit_bil=1` if laws explicitly cited as enabling or influencing project

3. Credit Attribution Language Guide

- *Direct causation:* enable, secure, fund, finance, deliver
- *Decision influence:* contributes to, influenced by, thanks to, because of
- *Partnership:* partnership with, working with, collaboration
- *Ceremonies:* announce, unveil, celebrate, ribbon-cut, host

4. Calibration Examples

1. “Thanks to President Biden’s leadership, we secured two billion dollars...” → `gives_credit=1`, `credit_biden=1`
2. “This project meets IRA ten percent bonus criteria.” → all zeros
3. “Our city council worked for years to land this plant.” → `gives_credit=1`, `credit_local=1`

5. Metadata Usage
Metadata keys:

- `speaker`, `role`, `state`, `district`, `city`
- `release_type`, `ira_funding`, `bil_funding`

Rules: self-credit if role matches speaker + first person; proxy releases only count quoted text; laws require explicit funding language unless metadata = YES.

6. Output Format

```
{
 "gives_credit":0,
 "credit_biden":0,
 "credit_senate":0,
 "credit_us_rep":0,
 "credit_governor":0,
 "credit_local":0,
 "credit_dem":0,
 "credit_gop":0,
 "credit_ira":0,
 "credit_bil":0
}
```

S6.4 Robustness to Alternative Codebook

The LLM annotation prompt in SI Appendix, S6.3, adopts a strict definition of credit in stage 1, saying that the IRA or BIL can only be credited if there's an explicit connection between these policies and the project. A possible concern is that these criteria under-count the frequency with which the IRA is credited.

As a robustness check, Fig. S14 reports results for an updated codebook that allows credit to be given in the following cases: the law is only mentioned as a goal or target, the project helps meet the law's goal, the law is mentioned only as background, the statement only discusses eligibility without confirming use, or the speaker only mentions helping to write the law. The results are qualitatively consistent.

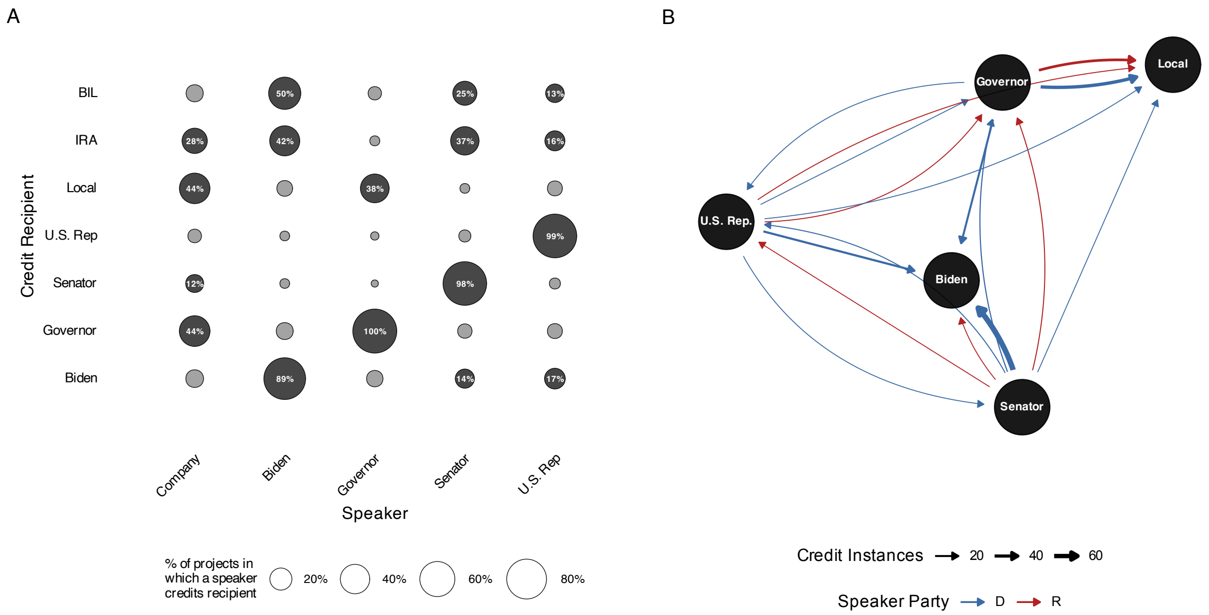

Fig. S14: Robustness to alternative codebook that counts any mention of the IRA, even if the speaker doesn't make a causal claim. Plot shows credit giving for clean energy manufacturing projects after the IRA (327 projects; Aug. 16, 2022–Dec. 30, 2024). **(A)** Share of projects in which each speaker credited each recipient. **(B)** Project-level credit network by speaker partisanship. Edge thickness denotes the share of projects. Blue lines denote Democratic speakers and red Republicans.

S6.5 Regression Models of Statement Giving

Table S31: Linear probability models of statement giving, by speaker

	Company	Governor	Senator	Rep	President
Intercept	0.995***	0.56**	0.28	0.12	0.63**
	(0.055)	(0.17)	(0.14)	(0.17)	(0.18)
Sector: EVs	-0.019	-0.018	0.0013	0.095	0.062
	(0.028)	(0.086)	(0.0748)	(0.084)	(0.084)
Sector: Solar	-0.043	-0.078	-0.0062	-0.128	0.079
	(0.033)	(0.064)	(0.0725)	(0.074)	(0.098)
Sector: Wind	-0.031	-0.213*	-0.067	-0.0044	0.11
	(0.030)	(0.089)	(0.069)	(0.0961)	(0.12)
Investment amount specified	0.018	0.185	0.218***	0.181*	0.156*
	(0.029)	(0.094)	(0.056)	(0.069)	(0.064)
Target jobs specified	-0.018	0.089	-0.047	-0.045	0.056
	(0.023)	(0.060)	(0.069)	(0.064)	(0.067)
Manufacturing investment	-0.0069	0.153*	0.069	0.119	0.0045
	(0.0198)	(0.074)	(0.076)	(0.078)	(0.0768)
Status: Operating	0.041	-0.044	-0.022	-0.07	-0.099
	(0.043)	(0.098)	(0.084)	(0.11)	(0.099)
Status: Pilot/Planned/Construction	0.037	0.118	0.076	0.043	-0.030
	(0.035)	(0.097)	(0.080)	(0.093)	(0.093)
County college share $(t-1)$	-0.0007	-0.065	-0.020	-0.054	-0.104*
	(0.0229)	(0.051)	(0.034)	(0.060)	(0.049)
County poverty share $(t-1)$	0.032**	0.047	-0.047	-0.024	-0.095**
	(0.011)	(0.038)	(0.029)	(0.047)	(0.034)
County foreign-born share $(t-1)$	-0.0098	-0.059	0.035	0.059	0.029
	(0.0147)	(0.053)	(0.034)	(0.046)	(0.041)
Median county housing costs $(t-1)$	0.022	0.080	-0.049	-0.086	-0.022
	(0.018)	(0.067)	(0.051)	(0.077)	(0.062)
Faster broadband access $(t-1)$	0.045	-0.054	0.030	0.115*	-0.148*
	(0.032)	(0.063)	(0.048)	(0.052)	(0.069)
County GDP (log) $(t-1)$	-0.014	0.126	0.14	0.061	0.13
	(0.041)	(0.094)	(0.10)	(0.151)	(0.12)
Labor force (log) $(t-1)$	-0.013	-0.210*	-0.20	-0.08	-0.16
	(0.035)	(0.096)	(0.11)	(0.15)	(0.12)
County unemployment rate $(t-1)$	-0.0051	-0.037	0.023	0.050	-0.013
	(0.0107)	(0.031)	(0.028)	(0.037)	(0.039)
Highway access	0.038	0.028	0.046	0.042	0.11
	(0.028)	(0.067)	(0.068)	(0.075)	(0.11)
County income pc $(t-1)$	0.014	0.024	-0.026	-0.009	-0.077
	(0.015)	(0.035)	(0.031)	(0.038)	(0.041)
Republican speaker		-0.290***	-0.416***	-0.188*	
		(0.071)	(0.069)	(0.082)	
County 2020 Biden vote share	-0.0034	0.112	0.0097	0.039	0.105**
	(0.0074)	(0.055)	(0.0450)	(0.065)	(0.035)
Republican Representative	-0.032	0.071	0.019		0.082
	(0.018)	(0.070)	(0.043)		(0.053)
Republican Governor	-0.041		0.0014	0.036	-0.184**
	(0.027)		(0.0661)	(0.074)	(0.066)
Swing state	0.0075	0.036	-0.077	0.020	0.151*
	(0.0220)	(0.067)	(0.064)	(0.084)	(0.059)
Competitive congressional district	-0.021	0.013	0.153*	0.15	-0.100
	(0.059)	(0.089)	(0.075)	(0.12)	(0.096)
State electricity price $(t-1)$	-0.0099	-0.111**	-0.040	-0.028	-0.034
	(0.0098)	(0.035)	(0.042)	(0.047)	(0.032)
State unionization rate $(t-1)$	-0.0037	-0.009	-0.026	-0.012	-0.069
	(0.0065)	(0.024)	(0.026)	(0.037)	(0.039)
2023	-0.040	-0.109	-0.016	0.083	-0.267***
	(0.022)	(0.065)	(0.082)	(0.063)	(0.066)
2024	-0.069**	-0.168*	-0.052	-0.016	-0.474***
	(0.020)	(0.074)	(0.077)	(0.082)	(0.052)
N	306	306	612	306	306
Adjusted R^2	-0.006	0.271	0.193	0.093	0.265

Notes: Each column reports a separate linear probability model for a speaker. The dependent variable equals 1 if the speaker issued a public project statement, 0 otherwise. Unit of analysis is the project–actor pair. Senators have higher observation counts (two per state). Some covariates are missing for projects without announced locations. Estimates are OLS with cluster-robust standard errors by state. Continuous covariates are county-standardized. Coefficients are percentage-point changes. "Republican speaker" indicates the party of the actor in each column header; "Republican Governor" and "Republican Representative" are contextual covariates capturing the party of these actors regardless of the speaker. * $p < 0.05$, ** $p < 0.01$, *** $p < 0.001$.

S6.6 Regression Models of Statement Credit Attribution

Table S32: Linear probability models of Biden/IRA credit, by speaker

	Outcome: Credited Biden/IRA (=1)				
	Company	Governor	Senator	Rep	President
Intercept	0.22	-0.064	0.80**	0.27	0.79***
	(0.12)	(0.112)	(0.23)	(0.15)	(0.22)
Sector: EVs	-0.054	0.076	0.091	-0.074	0.131
	(0.057)	(0.048)	(0.057)	(0.097)	(0.095)
Sector: Solar	0.349***	0.128	-0.075	0.085	0.076
	(0.088)	(0.064)	(0.099)	(0.190)	(0.126)
Sector: Wind	0.19	0.058	0.173	0.32	-0.22
	(0.11)	(0.107)	(0.095)	(0.19)	(0.15)
Investment amount specified	-0.035	-0.074	0.092	-0.14	0.06
	(0.069)	(0.069)	(0.109)	(0.14)	(0.16)
Target jobs specified	0.037	0.048	0.040	-0.04	-0.048
	(0.058)	(0.047)	(0.084)	(0.10)	(0.094)
Manufacturing investment	-0.098	-0.089	-0.19	0.044	-0.037
	(0.062)	(0.075)	(0.10)	(0.069)	(0.110)
Status: Operating	0.152	0.147	-0.14	0.065	-0.25
	(0.082)	(0.073)	(0.16)	(0.098)	(0.16)
Status: Pilot/Planned/Construction	0.116	0.087	0.087	0.051	-0.105
	(0.067)	(0.051)	(0.124)	(0.107)	(0.099)
County college share $(t-1)$	0.021	0.0085	-0.194**	0.123	-0.035
	(0.053)	(0.0517)	(0.057)	(0.077)	(0.079)
County poverty share $(t-1)$	-0.0056	-0.011	0.072	-0.011	-0.154**
	(0.0430)	(0.035)	(0.048)	(0.037)	(0.055)
County foreign-born share $(t-1)$	0.023	-0.076*	-0.177***	-0.015	0.076
	(0.045)	(0.036)	(0.038)	(0.054)	(0.052)
Median county housing costs $(t-1)$	0.098	-0.042	0.21	-0.032	-0.074
	(0.061)	(0.039)	(0.11)	(0.079)	(0.105)
Faster broadband access $(t-1)$	0.093	-0.019	0.099	-0.053	0.013
	(0.062)	(0.031)	(0.099)	(0.099)	(0.077)
County GDP (log) $(t-1)$	-0.066	-0.175	0.045	-0.12	0.19
	(0.097)	(0.088)	(0.138)	(0.17)	(0.20)
Labor force (log) $(t-1)$	-0.023	0.187*	0.075	0.088	-0.26
	(0.093)	(0.078)	(0.112)	(0.185)	(0.18)
County unemployment rate $(t-1)$	-0.065	0.046	-0.043	0.029	-0.077
	(0.034)	(0.033)	(0.029)	(0.035)	(0.055)
Highway access	-0.062	0.070	-0.067	0.093	-0.14
	(0.077)	(0.047)	(0.083)	(0.093)	(0.11)
County income pc $(t-1)$	-0.080*	0.052	-0.041	-0.17	-0.075
	(0.035)	(0.110)	(0.126)	(0.09)	(0.131)
Republican speaker		-0.12	-0.70***	-0.38**	
		(0.06)	(0.12)	(0.13)	
County 2020 Biden vote share	0.0058	0.019	-0.031	-0.018	0.043
	(0.0465)	(0.035)	(0.065)	(0.064)	(0.067)
Republican Representative	0.060	0.061	-0.249**		0.10
	(0.059)	(0.068)	(0.084)		(0.12)
Republican Governor	-0.026		0.096	0.19	-0.11
	(0.073)		(0.087)	(0.10)	(0.11)
Swing state	0.038	0.089*	-0.073	-0.090	-0.037
	(0.050)	(0.042)	(0.083)	(0.074)	(0.095)
Competitive congressional district	0.041	0.207*	-0.088	-0.091	0.21
	(0.101)	(0.099)	(0.092)	(0.139)	(0.16)
State electricity price $(t-1)$	-0.025	0.032	0.104	0.043	-0.135*
	(0.037)	(0.043)	(0.067)	(0.060)	(0.055)
State unionization rate $(t-1)$	0.021	0.042	-0.073*	0.061	0.028
	(0.032)	(0.023)	(0.033)	(0.044)	(0.050)
2023	-0.180*	0.044	-0.036	0.053	0.031
	(0.087)	(0.041)	(0.068)	(0.069)	(0.109)
2024	-0.144	0.12	-0.013	-0.037	0.27
	(0.082)	(0.10)	(0.093)	(0.084)	(0.14)
N	296	212	191	112	156
Adjusted R^2	0.131	0.195	0.398	0.266	0.073
Total statements	316	214	192	112	158

Notes: Each column reports a separate linear probability model for a speaker, conditional on the speaker making a project-related statement. The dependent variable equals 1 if the speaker credited the Biden Administration or IRA, 0 otherwise. Unit of analysis is the project–actor pair. N reflects observations with non-missing covariates; Total statements shows all statements by actor. Estimates are OLS with cluster-robust standard errors by state. Continuous covariates are county-standardized. Coefficients are percentage-point changes. * $p < 0.05$, ** $p < 0.01$, *** $p < 0.001$.

Table S33: Linear probability models of Governor credit, by speaker

	Outcome: Credited Governor (=1)				
	Company	Governor	Senator	Rep	President
Intercept	-0.028	0.87***	-0.15	0.017	-0.0095
	(0.160)	(0.09)	(0.12)	(0.124)	(0.1560)
Sector: EVs	0.0026	0.11**	0.128	0.019	-0.0073
	(0.0717)	(0.04)	(0.065)	(0.062)	(0.0564)
Sector: Solar	0.040	0.065	0.140*	0.067	0.0063
	(0.082)	(0.046)	(0.062)	(0.068)	(0.0714)
Sector: Wind	-0.13	-0.0021	-0.065	-0.045	-0.024
	(0.14)	(0.1097)	(0.042)	(0.054)	(0.127)
Investment amount specified	0.076	0.113	0.0011	-0.045	-0.043
	(0.070)	(0.084)	(0.0397)	(0.090)	(0.110)
Target jobs specified	0.101	0.115	0.085*	-0.059	0.020
	(0.064)	(0.079)	(0.037)	(0.058)	(0.064)
Manufacturing investment	0.173*	-0.016	0.012	-0.0082	0.093
	(0.073)	(0.057)	(0.035)	(0.0427)	(0.069)
Status: Operating	0.077	-0.058	0.0081	-0.031	0.14
	(0.086)	(0.042)	(0.0441)	(0.040)	(0.11)
Status: Pilot/Planned/Construction	0.287***	-0.119*	-0.017	0.081	0.078
	(0.074)	(0.052)	(0.049)	(0.057)	(0.115)
County college share $(t-1)$	-0.030	0.036	-0.042	-0.051	0.12
	(0.054)	(0.037)	(0.039)	(0.071)	(0.07)
County poverty share $(t-1)$	-0.062	-0.041	-0.0055	-0.022	0.152**
	(0.047)	(0.038)	(0.0211)	(0.037)	(0.052)
County foreign-born share $(t-1)$	-0.025	-0.078	-0.059*	0.022	-0.063*
	(0.040)	(0.046)	(0.025)	(0.026)	(0.028)
Median county housing costs $(t-1)$	-0.062	-0.027	-0.022	-0.006	0.047
	(0.076)	(0.067)	(0.043)	(0.060)	(0.069)
Faster broadband access $(t-1)$	-0.060	-0.019	0.010	0.016	-0.043
	(0.074)	(0.069)	(0.046)	(0.046)	(0.064)
County GDP (log) $(t-1)$	-0.11	1.9e-05	-0.22	-0.048	-0.24
	(0.14)	(7.6e-02)	(0.11)	(0.168)	(0.14)
Labor force (log) $(t-1)$	0.064	-0.030	0.132	0.0099	0.17
	(0.133)	(0.067)	(0.086)	(0.1859)	(0.11)
County unemployment rate $(t-1)$	-0.018	0.015	0.041	-0.034	0.040
	(0.041)	(0.030)	(0.025)	(0.025)	(0.034)
Highway access	-0.10	-0.034	0.088	0.081	0.124
	(0.07)	(0.054)	(0.055)	(0.059)	(0.082)
County income pc $(t-1)$	-0.021	-0.053	0.147*	0.043	0.11
	(0.035)	(0.067)	(0.068)	(0.099)	(0.11)
Republican speaker		-0.023	0.0074	0.068	
		(0.064)	(0.0467)	(0.043)	
County 2020 Biden vote share	0.133*	0.093	0.118	0.079	-0.074
	(0.059)	(0.052)	(0.062)	(0.061)	(0.056)
Republican Representative	0.071	-0.037	0.093		0.066
	(0.073)	(0.049)	(0.073)		(0.086)
Republican Governor	-0.033		-0.054	0.0043	-0.226**
	(0.064)		(0.050)	(0.0391)	(0.066)
Swing state	-0.030	0.035	-0.022	-0.0035	-0.042
	(0.076)	(0.052)	(0.052)	(0.0496)	(0.065)
Competitive congressional district	0.1	0.042	-0.066	-0.039	-0.048
	(0.1)	(0.037)	(0.057)	(0.049)	(0.077)
State electricity price $(t-1)$	0.013	0.050	-0.063	0.037	0.019
	(0.044)	(0.043)	(0.036)	(0.065)	(0.060)
State unionization rate $(t-1)$	-0.073*	-0.037	0.010	0.012	-0.036
	(0.030)	(0.045)	(0.023)	(0.022)	(0.036)
2023	0.035	-0.022	0.0066	-0.044	0.045
	(0.095)	(0.051)	(0.0692)	(0.077)	(0.076)
2024	-0.061	-0.0043	-0.071	-0.123	-0.045
	(0.109)	(0.0674)	(0.057)	(0.081)	(0.063)
N	296	212	191	112	156
Adjusted R^2	0.156	0.087	0.098	-0.086	0.061
Total statements	316	214	192	112	158

Notes: Each column reports a separate linear probability model for a speaker, conditional on the speaker making a project-related statement. The dependent variable equals 1 if the speaker credited the Governor, 0 otherwise. Unit of analysis is the project–actor pair. N reflects observations with non-missing covariates; Total statements shows all statements by actor. Estimates are OLS with cluster-robust standard errors by state. Continuous covariates are county-standardized. Coefficients are percentage-point changes. * $p < 0.05$, ** $p < 0.01$, *** $p < 0.001$.

Table S34: Linear probability models of Senator credit, by speaker

	Outcome: Credited Senator (=1)				
	Company	Governor	Senator	Rep	President
Intercept	-0.21*	0.00014	0.84**	0.011	-0.076
	(0.10)	(0.03142)	(0.24)	(0.039)	(0.057)
Sector: EVs	-0.092	5.7e-05	0.085	0.0015	0.00016
	(0.053)	(1.3e-02)	(0.070)	(0.0128)	(0.02049)
Sector: Solar	0.041	0.021	0.29***	-0.025	0.054
	(0.054)	(0.025)	(0.08)	(0.024)	(0.047)
Sector: Wind	0.0053	0.023	0.269**	0.092	0.085
	(0.0898)	(0.023)	(0.093)	(0.088)	(0.071)
Investment amount specified	0.012	0.017	0.08	0.0062	0.044
	(0.051)	(0.020)	(0.11)	(0.0234)	(0.036)
Target jobs specified	0.027	0.018	0.016	0.012	0.0012
	(0.030)	(0.015)	(0.090)	(0.014)	(0.0183)
Manufacturing investment	0.080	-0.013	0.0074	-0.018	0.023
	(0.047)	(0.017)	(0.1177)	(0.018)	(0.030)
Status: Operating	0.100*	-0.0028	0.18	0.041	-0.019
	(0.048)	(0.0295)	(0.13)	(0.043)	(0.046)
Status: Pilot/Planned/Construction	0.117**	-0.024	0.075	-0.0085	-0.024
	(0.039)	(0.016)	(0.118)	(0.0199)	(0.049)
County college share $(t-1)$	-0.019	0.0011	-0.165**	0.0053	0.034
	(0.029)	(0.0155)	(0.048)	(0.0154)	(0.035)
County poverty share $(t-1)$	-0.066	-0.030	0.030	0.017	0.018
	(0.033)	(0.017)	(0.039)	(0.017)	(0.021)
County foreign-born share $(t-1)$	0.033	0.0014	-0.062	0.0056	-0.012
	(0.021)	(0.0105)	(0.035)	(0.0092)	(0.018)
Median county housing costs $(t-1)$	-0.071	-0.054*	-0.046	-0.019	0.051
	(0.039)	(0.021)	(0.065)	(0.021)	(0.033)
Faster broadband access $(t-1)$	0.0025	0.0019	-0.042	-0.0064	-0.0041
	(0.0367)	(0.0199)	(0.068)	(0.0146)	(0.0170)
County GDP (log) $(t-1)$	-0.117	-0.036	-0.24	-0.099	-0.016
	(0.064)	(0.027)	(0.14)	(0.086)	(0.044)
Labor force (log) $(t-1)$	0.113	0.052	0.26	0.100	0.0021
	(0.059)	(0.038)	(0.14)	(0.086)	(0.0278)
County unemployment rate $(t-1)$	0.011	0.052	-0.016	-0.00031	0.0069
	(0.029)	(0.028)	(0.021)	(0.00451)	(0.0115)
Highway access	0.016	-0.015	-0.096	0.011	0.038
	(0.062)	(0.043)	(0.069)	(0.017)	(0.028)
County income pc $(t-1)$	0.006	0.034	0.25**	0.0064	-0.044
	(0.025)	(0.023)	(0.08)	(0.0199)	(0.048)
Republican speaker		-0.026	-0.38***	-0.027	
		(0.018)	(0.10)	(0.027)	
County 2020 Biden vote share	0.044	0.0036	0.075	-0.022	-0.036
	(0.033)	(0.0137)	(0.061)	(0.024)	(0.023)
Republican Representative	0.112**	0.015	-0.050		0.0003
	(0.036)	(0.025)	(0.062)		(0.0306)
Republican Governor	-0.058		0.068	-0.0082	-0.019
	(0.051)		(0.079)	(0.0206)	(0.025)
Swing state	-0.036	0.031**	-0.056	-0.031	0.011
	(0.038)	(0.010)	(0.051)	(0.027)	(0.015)
Competitive congressional district	-0.012	0.057	-0.117	0.090	0.027
	(0.089)	(0.054)	(0.065)	(0.068)	(0.087)
State electricity price $(t-1)$	0.013	0.0068	-0.0096	0.0071	0.001
	(0.027)	(0.0113)	(0.0396)	(0.0093)	(0.024)
State unionization rate $(t-1)$	-0.011	-0.0262**	0.054	-0.009	-0.0060
	(0.016)	(0.0087)	(0.035)	(0.014)	(0.0096)
2023	0.088	0.011	-0.083	0.017	0.022
	(0.044)	(0.035)	(0.090)	(0.020)	(0.017)
2024	0.076	0.015	-0.039	0.0018	0.013
	(0.053)	(0.040)	(0.101)	(0.0194)	(0.020)
N	296	212	191	112	156
Adjusted R^2	0.034	0.101	0.262	0.093	-0.060
Total statements	316	214	192	112	158

Notes: Each column reports a separate linear probability model for a speaker, conditional on the speaker making a project-related statement. The dependent variable equals 1 if the speaker credited the U.S. Senator, 0 otherwise. Unit of analysis is the project–actor pair. N reflects observations with non-missing covariates; Total statements shows all statements by actor. Estimates are OLS with cluster-robust standard errors by state. Continuous covariates are county-standardized. Coefficients are percentage-point changes. * $p < 0.05$, ** $p < 0.01$, *** $p < 0.001$.

Table S35: Linear probability models of Representative credit, by speaker

	Outcome: Credited Representative (=1)				
	Company	Governor	Senator	Rep	President
Intercept	-0.0056	0.013	0.029	0.44	0.021
	(0.0459)	(0.016)	(0.065)	(0.36)	(0.074)
Sector: EVs	-0.00017	-0.0077	0.059	-0.029	0.034
	(0.03502)	(0.0104)	(0.048)	(0.099)	(0.047)
Sector: Solar	0.0009	0.019	0.0099	0.238*	-0.010
	(0.0319)	(0.029)	(0.0299)	(0.094)	(0.021)
Sector: Wind	0.131	0.0038	0.15	0.27	0.046
	(0.086)	(0.0135)	(0.13)	(0.14)	(0.065)
Investment amount specified	-0.020	0.011	-0.010	-0.17	0.020
	(0.047)	(0.019)	(0.037)	(0.15)	(0.033)
Target jobs specified	0.0084	0.014	0.012	0.138	0.0094
	(0.0245)	(0.011)	(0.027)	(0.097)	(0.0233)
Manufacturing investment	0.021	0.0081	-0.010	-0.21	0.0046
	(0.034)	(0.0080)	(0.025)	(0.15)	(0.0341)
Status: Operating	0.055	0.024	-0.0047	0.39**	0.017
	(0.034)	(0.026)	(0.0262)	(0.11)	(0.034)
Status: Pilot/Planned/Construction	0.073*	-0.00011	0.015	0.303**	-0.016
	(0.027)	(0.00724)	(0.030)	(0.093)	(0.021)
County college share ($t-1$)	0.00076	-0.00056	-0.024	0.11	-0.012
	(0.02438)	(0.01285)	(0.029)	(0.11)	(0.016)
County poverty share ($t-1$)	0.023	-0.0098	0.015	0.207**	0.004
	(0.023)	(0.0145)	(0.017)	(0.069)	(0.019)
County foreign-born share ($t-1$)	-0.038	-0.0107	-0.039*	-0.173*	0.0013
	(0.022)	(0.0086)	(0.019)	(0.065)	(0.0087)
Median county housing costs ($t-1$)	0.058*	-0.027	0.031	0.47***	-0.0064
	(0.027)	(0.018)	(0.050)	(0.11)	(0.0228)
Faster broadband access ($t-1$)	0.0088	0.014	0.015	-0.27	-0.016
	(0.0239)	(0.017)	(0.016)	(0.15)	(0.019)
County GDP (log) ($t-1$)	-0.012	-0.0037	-0.038	0.53	-0.018
	(0.046)	(0.0117)	(0.053)	(0.27)	(0.041)
Labor force (log) ($t-1$)	0.0049	0.0039	0.024	-0.49	0.010
	(0.0423)	(0.0099)	(0.037)	(0.30)	(0.025)
County unemployment rate ($t-1$)	0.020	0.0098	0.035**	0.019	-0.006
	(0.017)	(0.0100)	(0.010)	(0.046)	(0.007)
Highway access	-0.04	-0.023	0.029	0.043	0.026
	(0.06)	(0.041)	(0.036)	(0.162)	(0.024)
County income pc ($t-1$)	0.0081	0.020	0.035	-0.30*	0.016
	(0.0134)	(0.015)	(0.041)	(0.11)	(0.019)
Republican speaker		-0.030	-0.017	-0.26*	
		(0.016)	(0.033)	(0.11)	
County 2020 Biden vote share	0.0092	0.0040	0.032	-0.14	-0.013
	(0.0269)	(0.0099)	(0.040)	(0.10)	(0.018)
Republican Representative	0.071	-0.0025	0.029		-0.054
	(0.045)	(0.0192)	(0.041)		(0.043)
Republican Governor	-0.110*		0.0081	0.131	-0.019
	(0.042)		(0.0322)	(0.093)	(0.017)
Swing state	-0.021	0.0172*	-0.082*	-0.029	-0.029
	(0.021)	(0.0064)	(0.031)	(0.099)	(0.027)
Competitive congressional district	-0.047	0.049	-0.0049	0.11	0.065
	(0.049)	(0.054)	(0.0587)	(0.11)	(0.064)
State electricity price ($t-1$)	-0.010	0.0026	0.155***	0.116	0.018
	(0.015)	(0.0068)	(0.028)	(0.061)	(0.040)
State unionization rate ($t-1$)	-0.032*	-0.0103	-0.038*	0.025	-0.0036
	(0.016)	(0.0064)	(0.017)	(0.047)	(0.0105)
2023	0.0077	-0.025	-0.056*	0.218*	-0.0083
	(0.0225)	(0.019)	(0.026)	(0.088)	(0.0223)
2024	0.065	-0.013	0.0025	0.30*	0.027
	(0.041)	(0.028)	(0.0312)	(0.13)	(0.019)
N	296	212	191	112	156
Adjusted R^2	0.052	-0.027	0.320	0.296	-0.004
Total statements	316	214	192	112	158

Notes: Each column reports a separate linear probability model for a speaker, conditional on the speaker making a project-related statement. The dependent variable equals 1 if the speaker credited the U.S. Representative, 0 otherwise. Unit of analysis is the project–actor pair. N reflects observations with non-missing covariates; Total statements shows all statements by actor. Estimates are OLS with cluster-robust standard errors by state. Continuous covariates are county-standardized. Coefficients are percentage-point changes. * $p < 0.05$, ** $p < 0.01$, *** $p < 0.001$.

S6.7 By Target Jobs

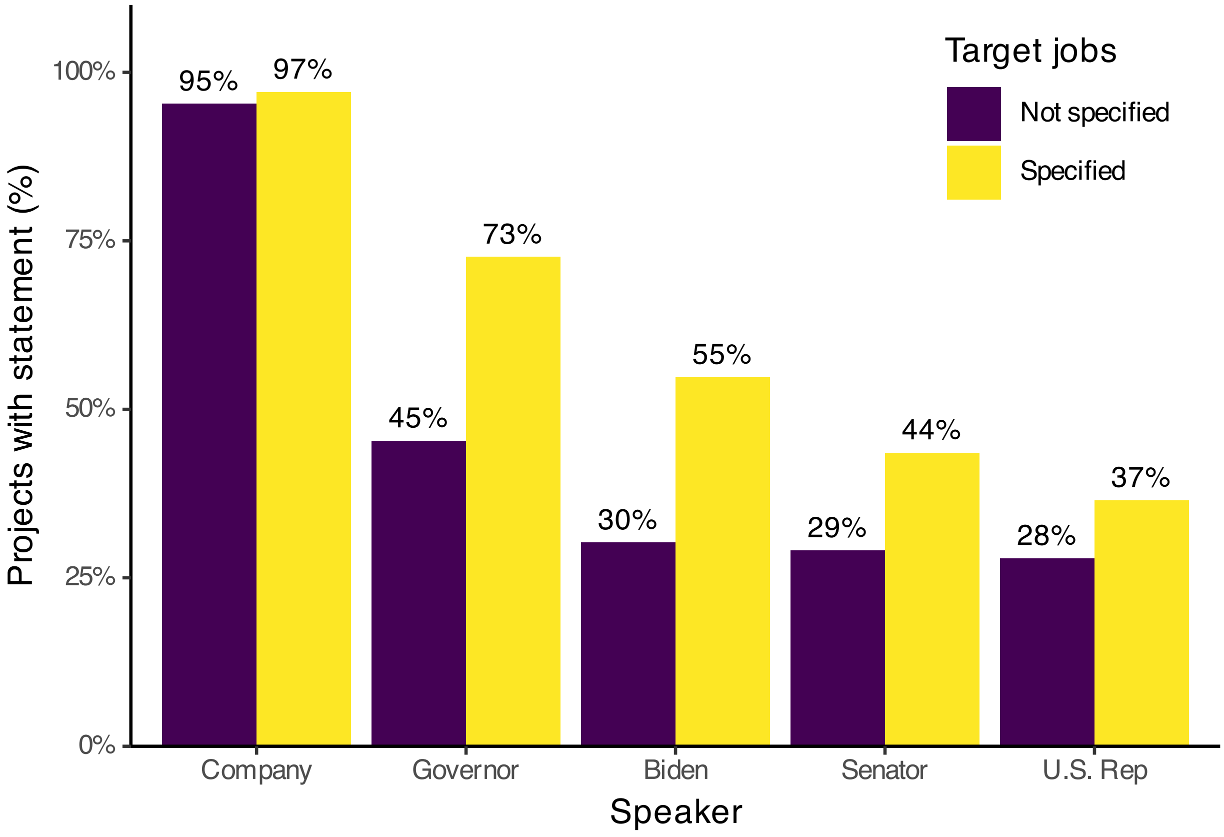

Fig. S15: Share of clean energy manufacturing projects with at least one public statement by companies and elected officials after the IRA's passage, stratified by job-creation targets (327 projects; Aug. 16, 2022–Dec. 30, 2024).

S6.8 Annotation Quality Analysis and Robustness

Table S36: Bias-adjusted linear probability models of Biden credit, by speaker (DSL)

	Outcome: Credited Biden (=1)				
	Company	Governor	Senator	Rep	President
Intercept	0.303*	-0.118	0.427*	0.213	0.920***
	(0.152)	(0.098)	(0.166)	(0.234)	(0.178)
Sector: EVs	0.045	0.072	-0.045	-0.165	-0.061
	(0.046)	(0.051)	(0.062)	(0.090)	(0.080)
Sector: Solar	0.276***	0.105	0.057	0.081	-0.163
	(0.067)	(0.065)	(0.076)	(0.123)	(0.103)
Sector: Wind	0.281*	-0.022	0.065	0.513**	-0.134
	(0.117)	(0.087)	(0.126)	(0.180)	(0.208)
Target jobs specified	0.097	0.030	0.055	-0.001	0.064
	(0.050)	(0.052)	(0.079)	(0.112)	(0.092)
Investment amount specified	0.036	0.002	0.127	-0.155	-0.087
	(0.064)	(0.064)	(0.104)	(0.135)	(0.096)
Status: Operating	-0.147	0.045	-0.030	0.053	-0.114
	(0.106)	(0.067)	(0.133)	(0.156)	(0.123)
Status: Pilot/Planned/Construction	-0.138	0.068	0.025	-0.011	0.025
	(0.099)	(0.065)	(0.130)	(0.150)	(0.070)
Manufacturing investment	-0.214***	-0.059	-0.236*	-0.023	0.024
	(0.060)	(0.048)	(0.095)	(0.101)	(0.091)
Highway access	-0.061	0.063	0.008	0.136	-0.112
	(0.059)	(0.050)	(0.088)	(0.137)	(0.103)
County college share $(t-1)$	-0.057	-0.009	-0.106	0.185**	0.029
	(0.039)	(0.049)	(0.067)	(0.065)	(0.068)
County poverty share $(t-1)$	-0.051	-0.012	-0.015	0.004	-0.012
	(0.034)	(0.034)	(0.041)	(0.069)	(0.057)
Faster broadband access $(t-1)$	0.098	-0.023	0.054	-0.008	-0.060
	(0.057)	(0.051)	(0.060)	(0.095)	(0.072)
County GDP (log) $(t-1)$	0.023	-0.128	0.059	-0.072	0.176
	(0.096)	(0.079)	(0.132)	(0.248)	(0.109)
County unemployment rate $(t-1)$	-0.057*	0.052	0.040	0.017	0.000
	(0.027)	(0.031)	(0.031)	(0.049)	(0.048)
Labor force (log) $(t-1)$	-0.109	0.157*	0.075	-0.072	-0.148
	(0.091)	(0.068)	(0.121)	(0.237)	(0.103)
County income pc $(t-1)$	-0.074**	0.063	-0.004	-0.101	-0.049
	(0.027)	(0.102)	(0.101)	(0.087)	(0.108)
County 2020 Biden vote share	0.029	0.034	-0.033	0.086	0.060
	(0.039)	(0.028)	(0.054)	(0.052)	(0.060)
Republican Representative	-0.026	0.095	-0.309**		0.120
	(0.051)	(0.057)	(0.099)		(0.090)
County foreign-born share $(t-1)$	0.042	-0.073**	-0.148***	-0.020	0.002
	(0.039)	(0.027)	(0.040)	(0.061)	(0.042)
Median county housing costs $(t-1)$	0.077	-0.024	0.050	-0.091	0.067
	(0.053)	(0.047)	(0.077)	(0.117)	(0.086)
Republican Governor	0.029		-0.120	0.279**	-0.130
	(0.053)		(0.084)	(0.094)	(0.084)
State electricity price $(t-1)$	-0.026	0.017	0.165***	0.062	-0.121
	(0.032)	(0.035)	(0.042)	(0.051)	(0.091)
State unionization rate $(t-1)$	0.026	0.051	-0.060*	-0.011	-0.023
	(0.027)	(0.027)	(0.028)	(0.051)	(0.042)
Swing state	0.021	0.071	-0.112	-0.084	0.042
	(0.050)	(0.042)	(0.067)	(0.100)	(0.073)
Competitive congressional district	0.044	0.164	-0.152	-0.237	0.254*
	(0.083)	(0.102)	(0.091)	(0.150)	(0.125)
2023	-0.116*	0.042	0.039	0.061	0.084
	(0.058)	(0.044)	(0.056)	(0.136)	(0.086)
2024	-0.073	0.126*	0.109	0.051	0.217*
	(0.064)	(0.061)	(0.059)	(0.132)	(0.097)
Republican speaker		-0.123**	-0.093	-0.403***	
		(0.044)	(0.088)	(0.106)	
N	28	28	29	28	28

Notes: Bias-adjusted regression estimates using DSL (Egami et al., 2024). Unit of analysis is the project–actor pair. Standard errors in parentheses. * $p < 0.05$, ** $p < 0.01$, *** $p < 0.001$.

Table S37: Bias-adjusted linear probability models of Senator credit, by speaker (DSL)

	Outcome: Credited Senator (=1)				
	Company	Governor	Senator	Rep	President
Intercept	-0.316*	-0.010	0.996***	-0.054	-0.126
	(0.139)	(0.027)	(0.018)	(0.053)	(0.090)
Sector: EVs	-0.159*	-0.000	0.018	-0.029	-0.007
	(0.062)	(0.016)	(0.017)	(0.022)	(0.022)
Sector: Solar	0.045	0.020	0.031	-0.069	0.063
	(0.077)	(0.032)	(0.027)	(0.038)	(0.057)
Sector: Wind	0.042	0.027	0.023	0.171	0.175
	(0.125)	(0.022)	(0.024)	(0.120)	(0.115)
Target jobs specified	-0.006	0.026	0.008	-0.001	0.004
	(0.054)	(0.018)	(0.010)	(0.028)	(0.024)
Investment amount specified	0.025	0.016	0.002	-0.029	0.072
	(0.083)	(0.020)	(0.012)	(0.034)	(0.057)
Status: Operating	0.197**	0.003	-0.004	0.081	-0.020
	(0.067)	(0.029)	(0.009)	(0.044)	(0.068)
Status: Pilot/Planned/Construction	0.189***	-0.019	-0.010	0.017	-0.022
	(0.053)	(0.019)	(0.011)	(0.035)	(0.063)
Manufacturing investment	0.126*	0.003	-0.007	-0.024	0.041
	(0.061)	(0.021)	(0.010)	(0.036)	(0.033)
Highway access	0.031	-0.027	-0.020	0.071	0.058
	(0.069)	(0.045)	(0.020)	(0.039)	(0.035)
County college share $(t-1)$	-0.024	0.000	-0.010	-0.007	0.053
	(0.041)	(0.012)	(0.009)	(0.022)	(0.043)
County poverty share $(t-1)$	-0.085*	-0.034	-0.003	-0.003	0.029
	(0.041)	(0.018)	(0.005)	(0.018)	(0.029)
Faster broadband access $(t-1)$	0.007	0.012	-0.001	-0.035	-0.005
	(0.060)	(0.021)	(0.004)	(0.033)	(0.022)
County GDP (log) $(t-1)$	-0.138	0.004	-0.000	-0.004	-0.019
	(0.089)	(0.027)	(0.017)	(0.143)	(0.057)
County unemployment rate $(t-1)$	0.009	0.053	0.004	0.011	0.017
	(0.038)	(0.029)	(0.005)	(0.012)	(0.028)
Labor force (log) $(t-1)$	0.137	0.012	-0.010	0.005	0.006
	(0.082)	(0.033)	(0.024)	(0.139)	(0.049)
County income pc $(t-1)$	0.001	0.017	0.011	-0.006	-0.062
	(0.029)	(0.020)	(0.008)	(0.049)	(0.074)
County 2020 Biden vote share	0.059	0.005	0.027	0.006	-0.058
	(0.046)	(0.012)	(0.023)	(0.021)	(0.035)
Republican Representative	0.159**	0.020	0.007		0.001
	(0.057)	(0.024)	(0.009)		(0.065)
County foreign-born share $(t-1)$	0.043	0.002	-0.005	0.015	-0.016
	(0.035)	(0.019)	(0.005)	(0.018)	(0.020)
Median county housing costs $(t-1)$	-0.102*	-0.052	0.005	-0.043	0.083
	(0.051)	(0.028)	(0.009)	(0.050)	(0.055)
Republican Governor	-0.082		0.036	-0.028	-0.019
	(0.071)		(0.032)	(0.025)	(0.048)
State electricity price $(t-1)$	0.031	0.004	-0.012	0.030	0.031
	(0.035)	(0.011)	(0.011)	(0.021)	(0.095)
State unionization rate $(t-1)$	-0.011	-0.030*	0.008	-0.045	-0.018
	(0.035)	(0.015)	(0.008)	(0.029)	(0.022)
Swing state	-0.041	0.029	0.001	-0.007	0.008
	(0.048)	(0.018)	(0.005)	(0.021)	(0.045)
Competitive congressional district	-0.077	0.051	-0.002	0.168	-0.011
	(0.104)	(0.051)	(0.006)	(0.088)	(0.095)
2023	0.121*	0.004	-0.019	0.045	0.029
	(0.053)	(0.034)	(0.018)	(0.030)	(0.046)
2024	0.106	0.018	-0.003	0.031	0.024
	(0.061)	(0.036)	(0.008)	(0.033)	(0.036)
Republican speaker		-0.042	-0.037	0.015	
		(0.031)	(0.033)	(0.022)	
N	28	28	29	28	28

Notes: Bias-adjusted regression estimates using DSL (Egami et al., 2024). Unit of analysis is the project–actor pair. Standard errors in parentheses. * $p < 0.05$, ** $p < 0.01$, *** $p < 0.001$.

Table S38: Bias-adjusted linear probability models of Governor credit, by speaker (DSL)

	Outcome: Credited Governor (=1)				
	Company	Governor	Senator	Rep	President
Intercept	0.118	1.000***	-0.155	-0.169	-0.085
	(0.221)	(0.000)	(0.128)	(0.200)	(0.141)
Sector: EVs	-0.040	-0.000	0.144*	0.080	0.043
	(0.097)	(0.000)	(0.057)	(0.091)	(0.064)
Sector: Solar	-0.015	-0.000	0.129*	0.116	0.064
	(0.100)	(0.000)	(0.062)	(0.120)	(0.082)
Sector: Wind	-0.149	-0.000	-0.057	0.076	0.238
	(0.129)	(0.000)	(0.054)	(0.089)	(0.134)
Target jobs specified	0.044	0.000	0.097*	-0.166	0.039
	(0.083)	(0.000)	(0.039)	(0.104)	(0.057)
Investment amount specified	0.141	-0.000***	-0.001	-0.050	0.084
	(0.100)	(0.000)	(0.044)	(0.097)	(0.112)
Status: Operating	0.029	-0.000	-0.009	0.082	0.130
	(0.160)	(0.000)	(0.051)	(0.094)	(0.114)
Status: Pilot/Planned/Construction	0.220	0.000	-0.036	0.288*	0.038
	(0.152)	(0.000)	(0.054)	(0.126)	(0.081)
Manufacturing investment	0.254**	-0.000	0.025	0.003	0.023
	(0.084)	(0.000)	(0.047)	(0.066)	(0.063)
Highway access	-0.072	-0.000***	0.086	0.147	0.222**
	(0.094)	(0.000)	(0.061)	(0.112)	(0.071)
County college share $(t-1)$	-0.033	-0.000	-0.026	-0.130	-0.011
	(0.067)	(0.000)	(0.048)	(0.099)	(0.078)
County poverty share $(t-1)$	-0.068	0.000	0.000	-0.118	0.115*
	(0.058)	(0.000)	(0.025)	(0.073)	(0.048)
Faster broadband access $(t-1)$	0.024	-0.000	0.022	0.023	-0.077
	(0.088)	(0.000)	(0.053)	(0.082)	(0.048)
County GDP (log) $(t-1)$	-0.034	-0.000	-0.146	-0.009	-0.334**
	(0.136)	(0.000)	(0.113)	(0.342)	(0.114)
County unemployment rate $(t-1)$	-0.051	0.000	0.047	-0.043	0.099*
	(0.053)	(0.000)	(0.028)	(0.060)	(0.048)
Labor force (log) $(t-1)$	0.003	-0.000	0.061	-0.020	0.320**
	(0.130)	(0.000)	(0.091)	(0.368)	(0.109)
County income pc $(t-1)$	-0.049	-0.000	0.113	0.095	0.267*
	(0.044)	(0.000)	(0.079)	(0.183)	(0.120)
County 2020 Biden vote share	0.127*	-0.000	0.112	0.153	-0.105
	(0.058)	(0.000)	(0.060)	(0.105)	(0.058)
Republican Representative	0.049	-0.000***	0.106*		-0.045
	(0.094)	(0.000)	(0.054)		(0.121)
County foreign-born share $(t-1)$	-0.020	-0.000	-0.058*	0.024	-0.074*
	(0.051)	(0.000)	(0.025)	(0.044)	(0.033)
Median county housing costs $(t-1)$	-0.143	-0.000	0.004	-0.076	0.050
	(0.077)	(0.000)	(0.047)	(0.100)	(0.072)
Republican Governor	-0.100		-0.072	0.029	-0.205*
	(0.097)		(0.056)	(0.070)	(0.088)
State electricity price $(t-1)$	0.065	-0.000	-0.067	0.036	0.126
	(0.058)	(0.000)	(0.035)	(0.063)	(0.111)
State unionization rate $(t-1)$	-0.089*	-0.000	-0.003	0.022	-0.103**
	(0.042)	(0.000)	(0.020)	(0.041)	(0.034)
Swing state	-0.067	0.000	0.014	0.015	-0.130
	(0.081)	(0.000)	(0.050)	(0.049)	(0.071)
Competitive congressional district	0.071	0.000***	-0.052	-0.005	-0.247*
	(0.131)	(0.000)	(0.055)	(0.076)	(0.123)
2023	0.006	0.000	-0.023	-0.036	0.153*
	(0.099)	(0.000)	(0.068)	(0.137)	(0.077)
2024	-0.111	-0.000	-0.087	-0.213	0.028
	(0.105)	(0.000)	(0.070)	(0.152)	(0.079)
Republican speaker		0.000	0.052	0.112	
		(0.000)	(0.055)	(0.076)	
N	28	28	29	28	28

Notes: Bias-adjusted regression estimates using DSL (Egami et al., 2024). Unit of analysis is the project–actor pair. Standard errors in parentheses. * $p < 0.05$, ** $p < 0.01$, *** $p < 0.001$.

Table S39: LLM Annotation Quality: Comparison to Human Ground Truth

Variable	N	Agreement	Cohen's κ	Precision	Recall	F1 Score
Gives Credit	100	88.0%	0.639	0.901	0.948	0.924
Biden Credit	100	98.0%	0.945	0.920	1.000	0.958
Governor Credit	100	88.0%	0.663	0.654	0.850	0.739

Notes: Quality metrics comparing LLM annotations (GPT-4) to human ground truth annotations for $N = 100$ randomly sampled statements. Agreement is raw percent agreement. Cohen's κ accounts for chance agreement; values > 0.6 indicate substantial agreement. Precision is P(Human=1 | LLM=1); Recall is P(LLM=1 | Human=1). F1 is the harmonic mean of Precision and Recall. For Governor Credit, McNemar's test for asymmetric errors yields $p = 0.149$, with the LLM over-attributing credit by 6.0 percentage points (95% bootstrap CI: [-1.0, 13.0]).

S7 Investment Timing

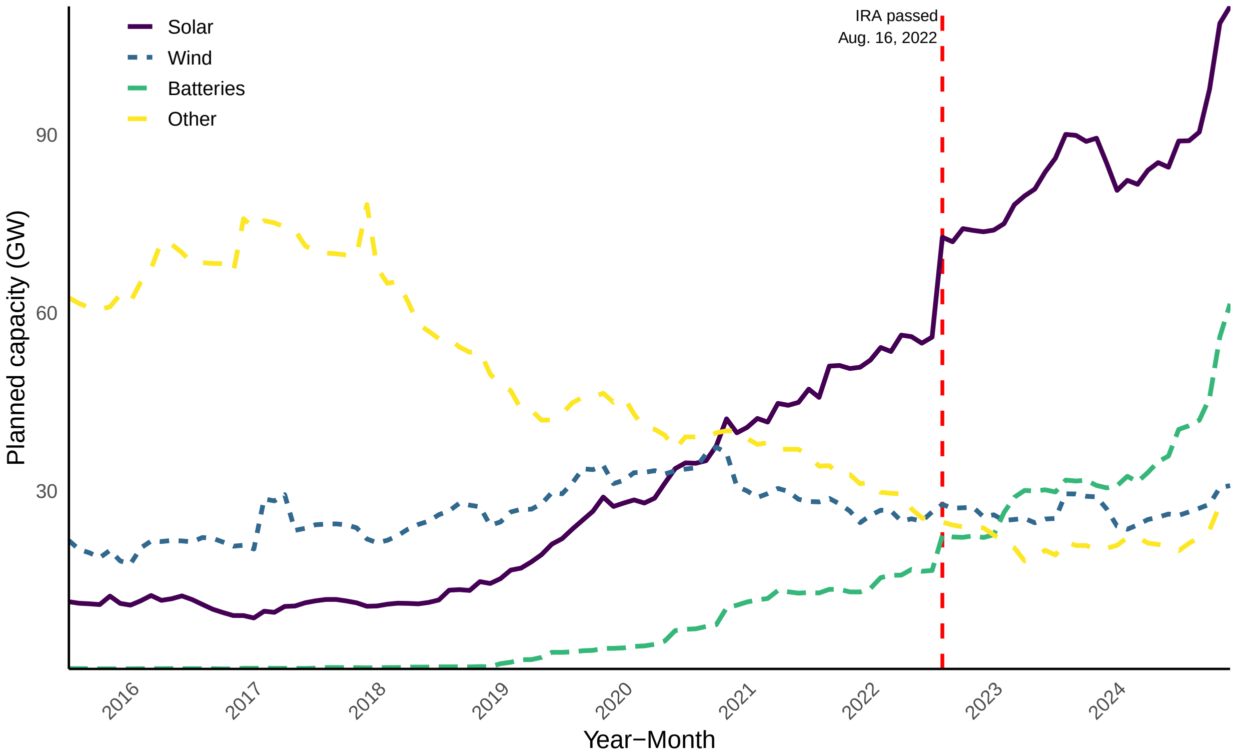

Fig. S16: Monthly planned power grid additions by technology, July 2015–December 2024. Data compiled from the EIA-860M reports.

S7.1 Clean Electricity Incentives

The IRA's clean-electricity tax reforms modified a long-running federal framework for investment and production credits rather than creating an entirely new system. In August 2022, the IRA established §48E (Clean Electricity Investment Credit) and §45Y (Clean Electricity Production Credit) as technology-neutral successors to the legacy ITC (§48) and PTC (§45), which phase out for projects placed in service after 2024. Although §48E and §45Y generally apply to facilities placed in service after December 31, 2024, long development and construction lead times meant that developers and financiers had incentives to begin advancing wind and solar projects soon after IRA enactment in anticipation of eligibility under the post-2024 regime. Treasury and IRS issued proposed regulations for §48E and §45Y in June 2024, with final rules announced in January 2025 and published shortly thereafter. Because these credits built on a familiar tax architecture, uncertainty was more limited than for entirely novel IRA provisions, even as important implementation questions—particularly around bonus credits and monetization—were resolved gradually through 2023 guidance on energy communities, domestic content, and credit transferability. As a result, it is plausible that expectations about these clean-electricity credits influenced project investment decisions during 2024, despite the lag in final regulatory guidance.

These investment responses also unfolded alongside the BIL, enacted in November 2021, which began appropriating funds and standing up programs in 2022–2023 that plausibly amplified the effect of IRA tax incentives by the time developers were making project decisions in 2023–2024. While the

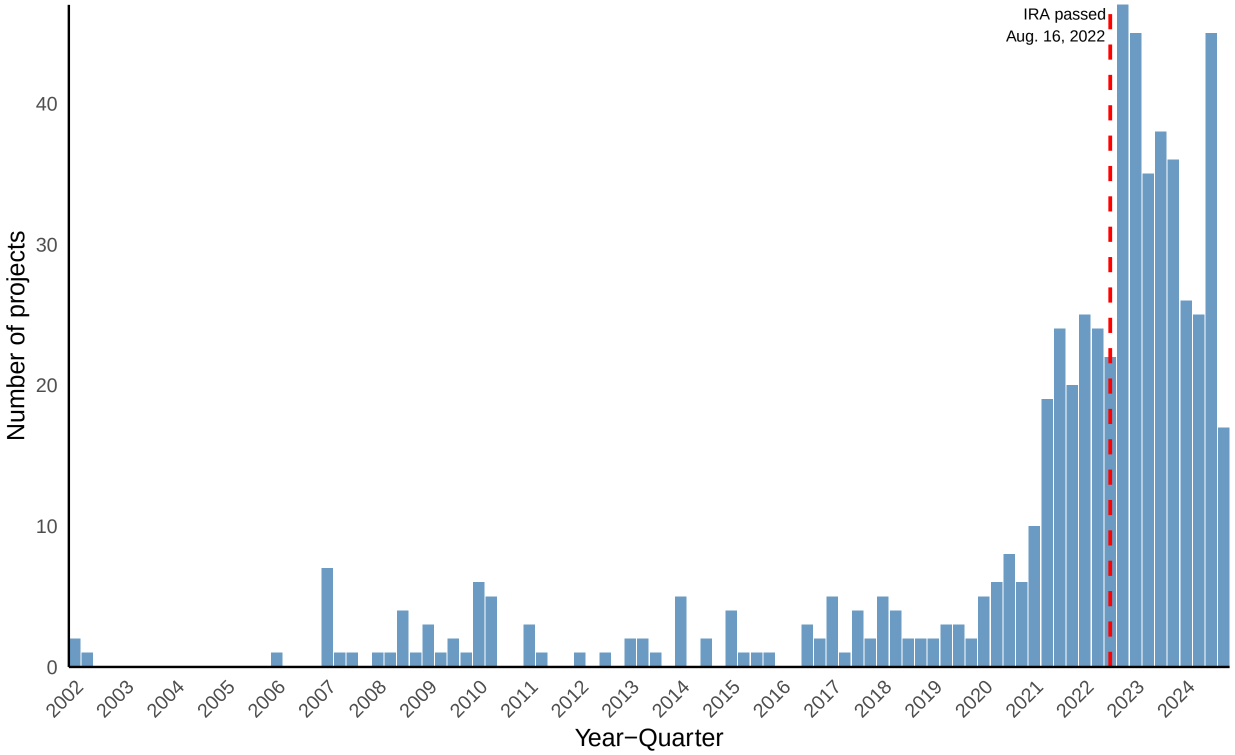

Fig. S17: Green manufacturing announcements in the United States, Q1-2002–Q4-2024. Data compiled from Jay Turner's Big Green Machine database and cover all project types.

BIL did not create core clean-electricity tax credits, it directed substantial early funding toward grid modernization, transmission expansion, and interregional capacity—most notably through DOE's Grid Deployment Office, the Grid Resilience and Innovation Partnerships (GRIP) program, and transmission facilitation authorities. As these programs moved from authorization to implementation during 2022–2024, they may have begun to reduce infrastructure and interconnection constraints.

S7.2 Clean Manufacturing Incentives

The primary clean manufacturing incentive is the IRA's §45X Advanced Manufacturing Production Credit. The IRA created §45X on August 16, 2022 as a per-unit production credit for eligible components, e.g. solar, batteries, critical minerals, and it went into effect for components produced and sold after December 31, 2022. The subsidy was active for 2023–2024 production and pricing decisions even before Treasury finalized the regulations. Treasury/IRS published proposed §45X regulations on December 15, 2023, which reduced uncertainty about definitions (e.g., what counts as an eligible component) during the 2024 investment window. Final §45X regulations were published October 28, 2024 (with technical corrections on November 26, 2024). The final rules were largely the same as the proposed regulation.[1] It's reasonable to assume that companies could justify capital expenditures in 2024 based on the statute, proposed rules, and reasonable reliance positions.

The IRA also contained revisions to §30D, the Clean Vehicle Credit, originally created in 2008 to give original equipment manufacturers up to $7,500 for plug-in electric drive motor vehicles. The

[1] https://www.pwc.com/us/en/services/tax/library/pwc-final-regulations-under-section-45x-address-eligible-components.html

IRA rewrote §30D in August 2022 to condition EV eligibility on (i) North American final assembly and (ii) increasing critical minerals and battery components sourcing thresholds. Treasury/IRS issued the proposed regulations on April 17, 2023. Treasury/IRS then issued proposed guidance on "excluded entities" on December 4, 2023, which mattered for supply-chain planning. By 2024, the basic sourcing regime was operating, and DOE's §30D explainer summarized the approaching foreign entity of concern-related constraints.

The IRA expanded major Title 17 authorities, creating the §1706 Energy Infrastructure Reinvestment (EIR) category and appropriating credit subsidy to support very large volumes of loan guarantees. DOE's Loan Programs Office (LPO) states that the IRA placed a total cap of up to $250 billion in §1706 loan guarantee authority and appropriated $5 billion in credit subsidy, with the authority expiring in FY2026. The LPO was operational in 2023–2024. By LPO's accounting, the Biden Administration period included 25 closed loans/loan guarantees and 28 active conditional commitments, and LPO reports that it closed 14 loans and loan guarantees in 2024 alone. [2]

BIL §40207 also created large grant programs for battery materials processing/manufacturing/recycling, and DOE implemented this in waves. The first tranche of awards arrived in October 2022, amounting to $2.8B to projects across multiple states, providing an early signal to private investors that federal industrial policy money was real and being deployed. The second prominent tranche came in September 2024, with $3B for 25 projects to strengthen the full battery supply chain. There were likely matched private capital, supplier contracting, and siting decisions made in anticipation of these awards and EV-credit sourcing rules.

[2] https://www.energy.gov/lpo/articles/lpo-year-review-2024

References for SI Appendix

Cinelli, Carlos and Chad Hazlett (2020). “Making Sense of Sensitivity: Extending Omitted Variable Bias.” *Journal of the Royal Statistical Society Series B* 82(1): 39–67.

Egami, Naoki et al. (2024). *Using Imperfect Surrogates for Downstream Inference: Design-based Supervised Learning for Social Science Applications of Large Language Models*. arXiv: 2306.04746 [stat].

Hirsch, Barry T. and David A. MacPherson (2003). “Union Membership and Coverage Database from the Current Population Survey: Note.” *ILR Review* 56(2): 349–354.
